# Molecular Biological Comparison of Dental Pulp- and Apical Papilla-Derived Stem Cells

**DOI:** 10.3390/ijms23052615

**Published:** 2022-02-27

**Authors:** Martyna Smeda, Kerstin M. Galler, Melanie Woelflick, Andreas Rosendahl, Christoph Moehle, Beate Lenhardt, Wolfgang Buchalla, Matthias Widbiller

**Affiliations:** 1 Department of Conservative Dentistry and Periodontology, University Hospital Regensburg, 93053 Regensburg, Germany; martyna.smeda@ukr.de (M.S.); melanie.woelflick@ukr.de (M.W.); andreas.rosendahl@ukr.de (A.R.); beate.lenhardt@web.de (B.L.); wolfgang.buchalla@ukr.de (W.B.); 2 Department of Operative Dentistry and Periodontology, Friedrich-Alexander-University Erlangen-Nürnberg, 91054 Erlangen, Germany; kerstin.galler@uk-erlangen.de; 3 Center of Excellence for Fluorescent Bioanalytics (KFB), University of Regensburg, 93053 Regensburg, Germany; christoph.moehle@exfor.uni-regensburg.de

**Keywords:** dental pulp stem cells, stem cells of the apical papilla, mesenchymal stem cells, regenerative endodontics, transcriptome

## Abstract

Both the dental pulp and the apical papilla represent a promising source of mesenchymal stem cells for regenerative endodontic protocols. The aim of this study was to outline molecular biological conformities and differences between dental pulp stem cells (DPSC) and stem cells from the apical papilla (SCAP). Thus, cells were isolated from the pulp and the apical papilla of an extracted molar and analyzed for mesenchymal stem cell markers as well as multi-lineage differentiation. During induced osteogenic differentiation, viability, proliferation, and wound healing assays were performed, and secreted signaling molecules were quantified by enzyme-linked immunosorbent assays (ELISA). Transcriptome-wide gene expression was profiled by microarrays and validated by quantitative reverse transcription PCR (qRT-PCR). Gene regulation was evaluated in the context of culture parameters and functionality. Both cell types expressed mesenchymal stem cell markers and were able to enter various lineages. DPSC and SCAP showed no significant differences in cell viability, proliferation, or migration; however, variations were observed in the profile of secreted molecules. Transcriptome analysis revealed the most significant gene regulation during the differentiation period, and 13 biomarkers were identified whose regulation was essential for both cell types. DPSC and SCAP share many features and their differentiation follows similar patterns. From a molecular biological perspective, both seem to be equally suitable for dental pulp tissue engineering.

## 1. Introduction

Regenerative endodontic procedures aim to replace an irreversibly inflamed or necrotic dental pulp. In order to generate new pulp-like tissue, researchers have successfully made use of stem cells, which is one of the three pillars in tissue engineering next to scaffold materials and signaling molecules [1,2,3,4]. Currently, two tissue engineering concepts for pulp regeneration can be differentiated, the first being based on cell transplantation and the second on cell-homing [5]. For the transplantation approach, stem cells and growth factors are inserted into a suitable scaffold and injected directly into the root canal. This requires storage and laboratory processing of stem cells beforehand, which is afflicted with high costs. However, a primarily cell-free approach based on cell-homing seems to be more practical for use in dental offices. In this case, no cells have to be transplanted but local stem cells are attracted from periapical tissues by recombinant signaling molecules or endogenous, dentin-derived growth factors and migrate into the root canal. Moreover, cell-homing can not only be used to restore the whole pulp but also parts of the tissue that are lost due to local inflammatory or necrotic processes [3,6,7].

Among various types of stem cells associated with dental tissues [8], especially dental pulp stem cells (DPSC) [9] and stem cells from the apical papilla (SCAP) [10] appear to be suitable sources for cell-homing as they are located in the root canal or in the apical papilla at the root tip and can thus give rise to new tissue. Interestingly, they share a common developmental origin as derivates of the pluripotent cranial neural crest cells that migrate to the first branchial arch and form the dental mesenchyme or ectomesenchyme [11,12,13]. During tooth development, interactions between the ectomesenchyme and the primitive oral epithelium result in the formation of a tooth bud. Subsequently, ectomesenchymal cells start to condense beneath and around the bud which leads to the formation of the dental papilla and the dental follicle. As the enamel organ continues to grow, forming first a cap and later a bell shape, the epithelial cervical loops enclose the cells of the dental papilla, initiating their transformation into the dental pulp. As soon as crown development is near completion, root formation starts with the apical proliferation of the cervical loops which now form a two-layered structure called Hertwig’s epithelial root sheath (HERS). HERS determines the shape of the later tooth root(s) and harbors mesenchymal cells, but has only limited growth potential. During root development, the dental papilla gradually transforms into radicular pulp tissue, whereas the follicle turns into periodontium [13,14,15]. Thus, the remaining dental papilla, which is termed apical papilla, can be found at the root end of immature teeth until root formation is completed. Histologically, it appears as a densified connected tissue separated from the pulpal tissue by a cell-rich zone.

Since both tissues can provide cells for pulp regeneration, the question arises whether the originating stem cells are equally suitable for this purpose. In addition to general qualities such as migration and proliferation, the ability to differentiate into a mineralizing odontoblast-like phenotype plays a particularly important role. Currently, there is still much to find out about genetic regulation during the differentiation of DPSC and SCAP, as only a modest number of studies directly compare the two stem cell types [10,16,17].

Therefore, the aim of this study was to outline parallels as well as differences between DPSC and SCAP isolated from the same donor regarding stemness, proliferation and viability, migration, and production of signaling molecules. The main focus was placed on the gene expression profiling of both cell types to identify genome-wide regulatory genes during induced differentiation.

## 2. Results

### 2.1. Stem Cell Characterization

**Figure 1:**
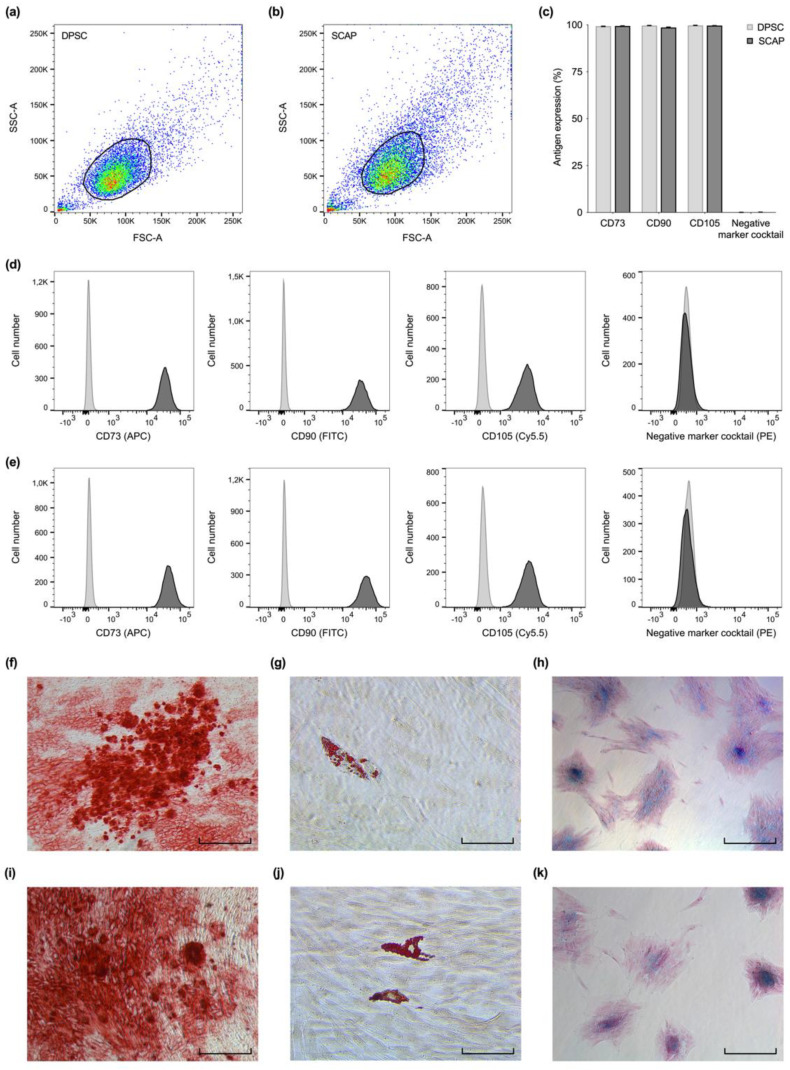
Stem cell characterization. Flow cytometric analysis (**a**–**e**) revealed similarities in size and granularity for dental pulp stem cells (DPSC) (**a**) and stem cells from the apical papilla (SCAP) (**b**). (**c**) DPSC and SCAP both expressed characteristic mesenchymal stem cell markers (CD73, CD90, and CD105), however, the markers CD34, CD45, CD11b, CD19, and HLA-DR were not detected. Exemplary overlay histograms of DPSC (**d**) and SCAP (**e**) show the control populations (light grey) and the specifically stained cells (dark grey). DPSC (**f**–**h**) and SCAP (**i**–**k**) both successfully entered the osteogenic (**f**,**i**), adipogenic (**g**,**j**), and chondrogenic lineage (**h**,**k**). Scale bars: 80 µm.

Overall, forward- and side-scatter signals revealed that both cell types were similar in size and granularity from a cytomorphological perspective (Figure 1a,b). A high proportion of DPSC and SCAP expressed mesenchymal stem cell markers, however, the markers of different origin (CD34, CD45, CD11b, CD19, and HLA-DR) were consistently undetected (Figure 1c–e). Cell culture experiments showed that both SCAP (Figure 1f–h) and DPSC (Figure 1i–k) were able to enter osteogenic, chondrogenic, and adipogenic lineage. Generally, Alizarin Red S stained large, widespread areas of mineralization whereas Oil Red O staining showed primarily scattered deposits of neutral lipids inside the cultured cells. The Alcian Blue 8GX dye revealed clusters of glycosaminoglycans as part of the recently formed cartilaginous matrix.

### 2.2. Cell Viability and Proliferation

**Figure 2:**
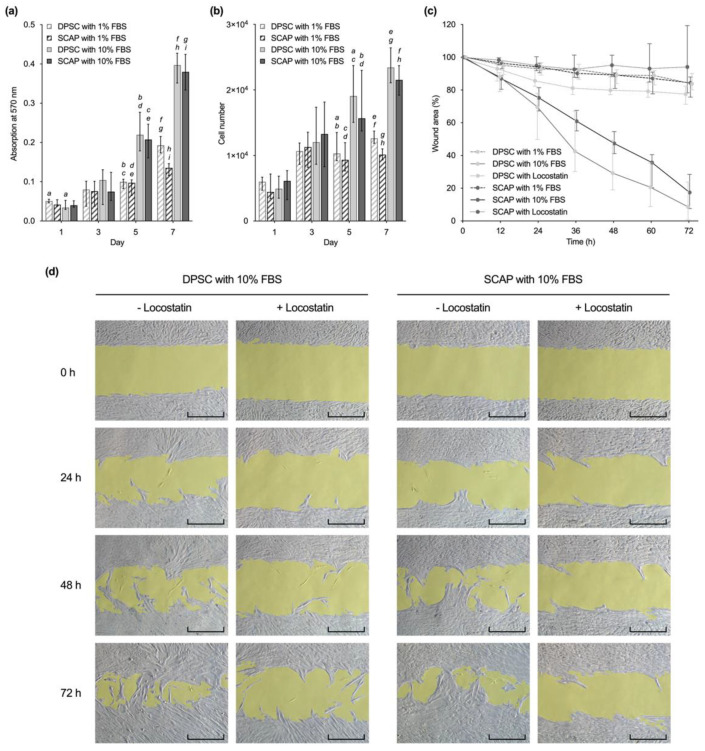
Comparison of (**a**) viability, (**b**) proliferation, and (**c**,**d**) migration of dental pulp stem cells (DPSC) and stem cells from the apical papilla (SCAP). (**a**,**b**) Viability and proliferation of DPSC and SCAP at different serum concentrations. Median values and 25–75% percentiles are based on three independent experiments performed in triplicates (*n* = 9). For each time point, the same lower-case letters indicate bars with statistically significant difference (*p* ≤ 0.05). (**c**) Cell migration of both cell types was determined over 72 h in triplicates and repeated two times (*n* = 12). Medians and 25–75% percentiles were normalized to the initial wound area, which was set to 100%. (**d**) Microscopic images of gap closure (gap area highlighted in yellow) by DPSC and SCAP cultured in alpha minimum essential medium (αMEM) with 10% fetal bovine serum (FBS) with and without Locostatin over 72 h. Scale bars: 400 µm.

Cell viability and proliferation assays showed similar patterns for SCAP and DPSC (Figure 2a,b). Viability and cell number increased until day 7 for both cell types and fetal bovine serum (FBS) concentrations. From day 5 on, DPSC and SCAP cultivated with 10% FBS showed a significantly higher viability and cell number compared to the ones cultivated with 1% FBS (*p* ≤ 0.003). However, cell number and viability of DPSC and SCAP showed no significant differences at the same culture conditions (*p* ≥ 0.1657).

### 2.3. Cell Migration

In the course of 72 h, wound healing was observed in all groups without the addition of inhibitors, whereby a clear influence of the serum concentration was observed (Figure 2c). At all times, cells cultivated with 10% FBS showed significantly higher migration rates than the ones cultivated with 1% FBS (*p* ≤ 0.0212). However, there were no statistically significant differences between DPSC with 10% FBS and SCAP with 10% FBS during 72 h (*p* > 0.9999). The addition of the migration inhibitor Locostatin to 10% FBS decreased migration rates of both DPSC and SCAP after 24 h with statistical significance (*p* ≤ 0.0002). The microscopic images show continuous sheath migration over 72 h for SCAP as well as DPSC cultures (Figure 2d). Locostatin largely suppressed this without evidence of cytotoxic effects.

### 2.4. Release of Signaling Molecules

**Figure 3:**
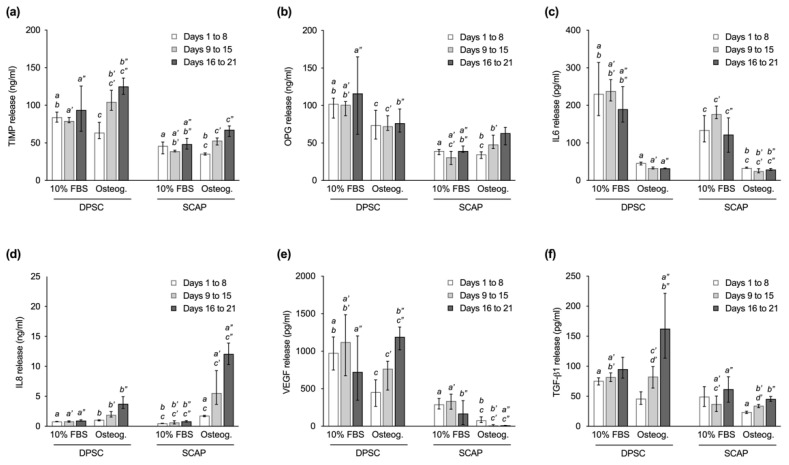
Release of signaling molecules by dental pulp stem cells (DPSC) and stem cells from the apical papilla (SCAP) in the initial phase (days 1 to 8), intermediate phase (days 9 to 15), and late phase (days 16 to 21) of culture. Graphs show the secretion of (**a**) tissue inhibitor of metalloproteinase (TIMP), (**b**) osteoprotegerin (OPG), (**c**) interleukin 6 (IL-6), (**d**) interleukin 8 (IL-8), (**e**) vascular endothelial growth factor (VEGF), and (**f**) transforming growth factor beta 1 (TGF-β1). Median values and 25–75% percentiles are based on three independent experiments performed in triplicates (*n* = 9). For each culture phase, equal lower-case letters indicate pairs that were found significantly different (*p* ≤ 0.05). One or two apostrophes were added for the intermediate or late phase for a better overview.

In general, DPSC released more osteoprotegerin (OPG), tissue inhibitor of metalloproteinase (TIMP), vascular endothelial growth factor (VEGF), and transforming growth factor beta 1 (TGF-β1) than SCAP (Figure 3). While the amount of interleukin 6 (IL-6) was similarly reduced in DPSC and SCAP during osteogenic differentiation (*p* ≤ 0.0056), significantly more interleukin 8 (IL-8) was secreted in the SCAP cultured with StemPro^®^ compared to medium with 10% FBS (*p* < 0.0001). Regarding the culture conditions, less IL-6 was released from both cell types during induced differentiation (*p* ≤ 0.0056). At the same time, more IL-8 was secreted in osteogenic cultures with statistical significance for SCAP (*p* < 0.0001). While DPSC showed an increasing VEGF release during osteogenic differentiation, SCAP showed no such tendencies and VEGF secretion was even significantly lower compared to DPSC in osteogenic culture (*p* ≤ 0.0082). Similarly, the release of TIMP was higher for DPSC compared to SCAP in the respective culture conditions (*p* ≤ 0.0331). The release of TGF-β1 was significantly higher during induced differentiation of DPSC compared to SCAP for the intermediate and late phase (*p* ≤ 0.0038).

With regard to the time course, cells cultured with StemPro^®^ showed a continuous increase in the release of TIMP, IL-8, and TGF-β1 from day 1 to 21, which was statistically significant for all three signaling molecules when comparing the initial and the late phase (*p* < 0.0001). In addition, during osteogenic differentiation, an increasing production of VEGF and OPG was observed in DPSC and SCAP, respectively, with statistical significance between the initial and the late phase (*p* ≤ 0.0001). No relevant changes of expression levels were observed for IL-6 for both cell types.

### 2.5. Gene Expression Profiling

**Figure 4:**
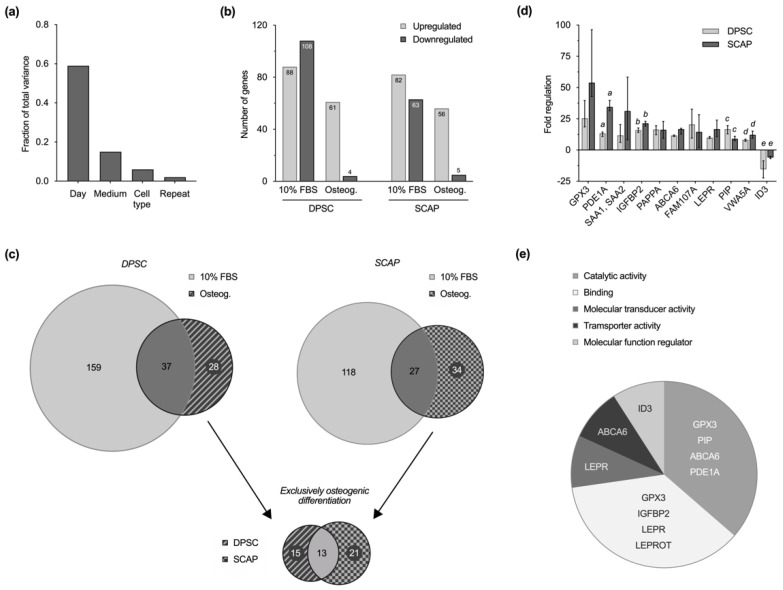
Transcriptome analysis. (**a**) Fraction of total variance highlights the impact of culture time on gene regulation among other culture parameters. (**b**) Number of up- and downregulated genes within the investigated groups on day 14 compared to day 1. Similarities between dental pulp stem cells (DPSC) and stem cells from the apical papilla (SCAP) are visible in standard cell culture and osteogenic differentiation. Fewer genes were regulated during differentiation and most of them were upregulated on day 14 of cell culture. (**c**) VENN diagrams for each cell type reveal subsets of genes that are specifically regulated in standard culture or osteogenic differentiation. Comparison of exclusively regulated genes during osteogenic differentiation of DPSC (28) and SCAP (34) enables the identification of 13 key genes that play a key role in osteogenesis of both cell types. (**d**) Microarray validation by quantitative reverse transcription PCR (qRT-PCR). Regulation of selected genes in DPSC and SCAP during induced differentiation at day 14 as median values and 25–75% percentiles. Regulations of all genes were statistically significant compared to baseline (*p* ≤ 0.0236), whereas equal lower-case letters for each gene indicate pairs (DPSC and SCAP) with significant differences (*p* ≤ 0.05). (**e**) PANTHER classification of 13 key genes according to molecular function. Genes with unknown or unapproved function are not shown (*SAA2*/*SAA2-SAA4*/*SAA4*, *SAA1*, *GPM6B*, *FAM107A*, *PAPPA*, *VWA5A*).

The transcriptome analysis showed that, with regard to the culture parameters, the culture time in particular has an impact on gene regulation, whereas the culture medium as well as the cell type were only secondary variables. The comparison of the repeats demonstrates the high reproducibility of the experiments (Figure 4a). The observation of up- and downregulation of genes over a culture period of 14 days revealed for both cell types that fewer genes were regulated during osteogenic differentiation compared to the control with 10% FBS (Figure 4b). The majority of genes that were differentially expressed during induced osteogenesis were upregulated on day 14, only a few were downregulated.

Of all the regulated genes, those that are differentially regulated exclusively in the course of osteogenic differentiation were identified for both cell types; these were 28 in DPSC and 34 in SCAP (Supplementary File 1). Of particular interest is the intersection of 13 regulated genes that were reproducibly regulated in the course of induced osteogenesis in both DPSC and SCAP (Figure 4c). Thus, 13 genes were identified that were exclusively regulated in both cell types during osteogenic differentiation, 12 of which were over-expressed and 1 of which was diminished (Table 1). The results of the microarray analysis were validated and confirmed by quantitative reverse transcription PCR (qRT-PCR) of selected genes. Figure 4d shows the regulation of gene expression in DPSC and SCAP during induced differentiation at day 14 compared to day 1.

With the help of the PANTHER classification system, the respective genes were attributed to their respective molecular functions (Figure 4e). Several genes were assorted to the categories of catalytic activity (*GPX3*, *PIP*, *ABCA6*, *PDE1A*), binding (*GPX3*, *IGFBP2*, *LEPR*/*LEPROT*), molecular transducer activity (*LEPR*), transporter activity (*ABCA6*), and molecular function regulator (*ID3*); however, the molecular functions of some remain unknown (*SAA2*/*SAA2-SAA4*/*SAA4*, *SAA1*, *GPM6B*, *FAM107A*, *PAPPA*, *VWA5A*).

## 3. Discussion

Based on these results, both DPSC and SCAP appear to be suitable cells for regenerative endodontic approaches. It is important to consider, however, that their availability also depends on the stage of inflammation or necrosis in the dental pulp. When inflammation spreads, e.g., during carious decay, it is initially located adjacent to the area of bacterial invasion, whereas the rest of the pulp is initially not affected [7,18]. In this scenario, the irreversibly damaged tissue can be removed selectively, leaving behind a healthy pulp rich in mesenchymal stem cells and capable to regenerate the lost tissue [3,5]. Once the whole pulp is irreversibly inflamed or necrotic, this source disappears, leaving the apical papilla at the root tip of juvenile patients [19]. Residing stem cells in the apical papilla reportedly survive pulpal necrosis [20] and thus remain available for endodontic regeneration. They can either be actively brought into the canal via induced bleeding as part of revitalization procedures or migrate in the course of cell-homing approaches to form pulp-like tissue.

### 3.1. Stem Cell Characterization

Obviously, both mesenchymal stem cell types examined in this study originate from two evolutionary and anatomically closely linked tissues and reside in niches accessible for cell homing strategies for pulpal regeneration. While SCAP can be found in the apical papilla at the tips of immature roots, DPSC are located perivascularly inside the dental pulp and express vasculature-specific antigens such as α-smooth muscle actin, CD146, and the pericyte marker 3G5 [10,21]. Though they reflect different developmental stages of the former dental papilla, DPSC and SCAP have common features, e.g., the expression of the mesenchymal progenitor marker STRO-1 [10].

In accordance with previous studies, our analysis revealed both cell types to be positive for the mesenchymal stem cell markers CD73, CD90, and CD105, as well as a multi-lineage differentiation potential [10,22,23,24], both of which characterize mesenchymal stem cells according to Dominici et al. [25]. As reported previously, a pronounced osteogenic but rather weak adipogenic differentiation potential was observed for both DPSC and SCAP after Alizarin Red S and Oil Red O staining [17,26,27]. Thus, especially when comparing the two tissues of one donor, both the apical papilla and the dental pulp seem to represent a comparably suitable reservoir for multipotent stem cells. In regard to the pulp tissue’s functionality, it is particularly their capability to transform into a mineralizing phenotype that is welcome in regenerative endodontic approaches.

### 3.2. Cell Viability, Proliferation, and Migration

Likewise, cell viability and proliferation as well as the ability for migration are important cell properties for endodontic regeneration. The goal is to achieve cell migration from their niches into a three-dimensional scaffold and transformation into a pulpal tissue [5].

In this regard, no significant differences were observed between DPSC and SCAP under the same culture conditions. These findings coincide with results from a recent study by Park et al. [17], which also reported a similar proliferation and colony-forming potential for SCAP and DPSC. Moreover, higher serum concentrations in cell culture media (10% FBS) had an impact on viability, proliferation, and migration of both cell types, as was already seen for SCAP [28]. The addition of Locostatin, a migration inhibitor targeting Raf kinase inhibitor protein (RKIP), significantly decreased the migration of DPSC and SCAP in a similar manner. In this context, it was shown that Locostatin inhibits not only the migration of terminally differentiated cells, but also of dental stem cells [29].

Nevertheless, controversial statements are made about the performance of DPSC and SCAP in the literature as well. Previous studies reported higher viability, proliferation, and migration rates for SCAP compared to DPSC [10,16,30]. Sonoyama et al. [10] defined SCAP as early progenitors from a developing tissue that are more suitable for the use in regenerative procedures due to higher expression levels of survivin, an inhibitor of apoptosis, and a higher telomerase activity, both relevant in terms of cell proliferation. However, these studies did not generally use cells from the same donor in the experiments and also differed in the culture conditions, e.g., serum content or passage. It also has to be mentioned that cell cultures, especially the ones from developing tissues such as SCAP, might contain several types of undifferentiated cells, resulting in different cell proliferation and differentiation potential [31].

The findings of this study suggest that DPSC and SCAP have similar viability and capacity for cell proliferation and migration, therefore they both fulfil the requirements for tissue engineering techniques. Nevertheless, it should also be mentioned here that although the results are based on cells derived from one donor, it cannot be ruled out that donor-dependent differences may also occur.

### 3.3. Release of Signaling Molecules

In addition to the culture behavior of the DPSC and SCAP, their signaling characteristics play an important role in the course of tissue formation. A variety of signaling molecules are known to be secreted by mesenchymal cells with different functions in terms of migration, differentiation, and inflammation, however, stem cell cultures from the dental pulp or apical papilla from a single donor have rarely been investigated in comparison [32]. Thus, a selection of relevant proteins was specifically quantified during induced osteogenic differentiation in order to reveal the signaling potential of DPSC and SCAP in terms of pulp tissue formation (TIMP, OPG, IL-6, IL-8, VEGF, and TGF-β1). In general, relevant differences regarding the cell type, the time of culture, and/or the culture conditions were observed for all signal molecules investigated, which shall be discussed in more detail subsequently.

TIMP, a multifunctional cytokine, was secreted by both DPSC and SCAP during standard cell culture with slightly higher levels in DPSC cultures. Despite the type of medium having no considerable impact, a time-dependent increase of TIMP was observed during osteogenic differentiation. Assuming that TIMP not only influences processes such as cell growth, apoptosis and angiogenesis, but also controls the activity of matrix metalloproteinases and therefore plays a key role in the remodeling of extracellular matrix, it can be suggested that DPSC have a slightly higher differentiation potential than SCAP [33]. As SCAP, which originate from a developing tissue, probably provide a less differentiated phenotype according to Sonoyama et al. [10], they probably release less TIMP at the beginning, which increases during osteogenic differentiation.

A similar observation was made for OPG, showing lower secretion levels in SCAP cultures and an increase during induced osteogenic differentiation. According to literature, OPG is supposed to be a pro-osteogenic factor that has the ability to prime undifferentiated mesenchymal stem cells towards mineralization [34]. Furthermore, it counteracts osteoclastogenesis and is released constitutively to limit the differentiation of osteoclasts and thus controls bone remodeling processes [35].

TGF-β1, a promotor of odontoblast formation and key protein in dentin mineralization [36], was found in a greater extent in DPSC cultures. Furthermore, increasing secretion was evident in the course of differentiation of both DPSC and SCAP. Interestingly, previous studies also reported higher concentrations of neurotrophins and growth factors (NT-3, BMP-4, and TGF-β3) in DPSC [32], leading to the suggestion that they give more pronounced paracrine signals related to odontoblast differentiation compared to SCAP. Since TGFs and BMPs play an important role in dentin secretion and are also embedded within it [37], it is conceivable that they are prevalent in terminally differentiated DPSC.

Considering that TGF-β1 is also a known promotor of angiogenesis by stimulating the production of VEGF mRNA [38,39], it is not surprising that higher levels of VEGF were detected in DPSC cultures. However, it was unexpected that SCAP, unlike DPSC, secreted moderate amounts of VEGF in the standard medium but almost none in the course of induced differentiation. A previous study on osteogenesis of mesenchymal stem cells reported that VEGF does not only stimulate mineralization but is also secreted in a differentiation dependent manner [40]. A possible conclusion would be that SCAP are isolated in a less differentiated state and accordingly lag behind in culture.

IL-6, a pleiotropic cytokine, was secreted in high levels by both DPSC and SCAP under standard culture conditions. It is not only involved in regeneration, inflammation, and the activation of immune cells, but also an important factor to maintain homeostasis [41]. Studies showed that regenerative and anti-inflammatory properties are mediated by a classic signaling pathway where the IL-6 receptor is membrane-bound on target cells. In contrast, binding to the soluble IL-6 receptor leads to the activation of pro-inflammatory activities through the trans-signaling pathway [42,43]. Both pathways seem to be used by DPSC and SCAP, showing the versatility needed for tissue engineering and the maintenance of a healthy dental pulp [44,45].

In contrast, IL-8 was released only in low levels by DPSC as well as SCAP under standard conditions; however, a considerable increase was observed during osteogenic differentiation, especially in SCAP. Interestingly, a recent study also measured high levels of proinflammatory cytokines such as IL-8 released by SCAP exposed to different anaerobic oral bacteria [46]. In general, IL-8 promotes osteoclastogenesis and is associated with bone resorption [47]. Moreover, it is supposed to have a chemotactic effect on mesenchymal stem cells [48]. These findings align with the results of this study, showing IL-8 as a key factor during osteogenic differentiation.

Overall, DPSC and SCAP appear to have many similarities in terms of migration, proliferation, and differentiation. In view of observations from animal studies or case reports, this leads to the assumption that both cell types have a similar potential to migrate into the root canal and form mineralizing tissue during regenerative procedures. Interestingly, the profile of secreted signaling molecules suggests that SCAP are in a less differentiated state than DPSC and thus may be more versatile.

### 3.4. Gene Expression Profiling

This study was one of the first to comprehensively compare gene expression of DPSC and SCAP isolated from the same donor and thus eliminating donor and culture-specific variables such as age, developmental state, isolation technique, or cell passage. Deeper insights in regulatory mechanisms were gained by comparison of stem cells cultivated under standard culture osteogenic conditions, where cells undergoing induced osteogenic differentiation represent the processes that are expected in the context of regenerative endodontic applications. Mesenchymal progenitor cells must form a mineralizing phenotype in the course of cell homing, i.e., differentiate osteogenically or odontogenically, as it is often termed in the endodontic context. The comprehensive transcriptome analysis allowed to define genome-wide regulatory genes that play a key role during the differentiation processes of odontogenic stem cells. Strict analysis settings were established to focus on relevant genes that play a central role in the course of differentiation, regardless of cell type. The aim was to gain more insight into the regulation of cell differentiation in order to possibly control or optimize this process clinically. Comparisons concerning differential gene expression were made between day 1 and day 14 as the culture parameter “time” appeared to be the most influential one. In both DPSC and SCAP, fewer genes were significantly regulated during the differentiation process than during standard cell culture. Thus, unspecifically regulated genes of the control cultures were deliberately excluded in the course of further analysis to identify exactly those genes that were exclusively up- or downregulated during induced osteogenic differentiation. Particularly, the final comparison of both cell types allowed to narrow this search down to only 13 genes that play a key role in induced differentiation of both DPSC and SCAP. Finally, all identified genes were categorized according to their molecular function by the PANTHER system: catalytic activity (*GPX3*, *PIP*, *ABCA6*, *PDE1A*), binding (*GPX3*, *IGFBP2*, *LEPR*/*LEPROT*), molecular transducer activity (*LEPR*), transporter activity (*ABCA6*), molecular function regulator (*ID3*) and unknown molecular function (*SAA2*/*SAA2-SAA4*/*SAA4*, *SAA1*, *GPM6B*, *FAM107A*, *PAPPA*, *VWA5A*).

The upregulated genes that are associated with catalytic activity usually code for macromolecules with enzyme function that can catalyze biochemical reactions. Accordingly, glutathione peroxidase 3 (*GPX3*) was the most highly expressed gene detected with microarray as well as PCR analysis. In analogy to previous reports on osteogenically differentiated human mesenchymal stem cells, it was upregulated in both DPSC and SCAP on day 14 [49]. Glutathione peroxidase 3 protects cells from oxidative damage by reduction of hydrogen peroxide and is suspected to be a key factor during osteogenic differentiation of mesenchymal stem cells [50].

Furthermore, the prolactin-induced protein (*PIP*) is a secreted glycoprotein with endonuclease activity that is involved in proteolysis and immunological processes. Though little is known about its exact physiological function, studies observed that prolactin-induced protein is able to bind to CD4 receptors of T lymphocytes or macrophages and the Fc fragment of immunoglobulin G, and therefore supposedly has immunomodulatory capabilities [51,52]. Its high expression on day 14 of osteogenic differentiation is in line with the findings of Li et al. [53], where *PIP* upregulations were observed during the osteogenic induction of periodontal ligament stem cells (PDLSC). They concluded that the upregulation seems to be caused by dexamethasone, a glucocorticoid which is also part the StemPro^®^ Osteogenesis Differentiation Kit. Dexamethasone supposedly stimulates *PIP* expression by a glucocorticoid receptor-dependent transcriptional activation. Moreover, they observed that a knockdown of *PIP* and its fibronectin-degrading properties even enhanced mineralization of PDLSC [53].

The ATP-binding cassette sub-family A member 6 belongs to the ABC transporter family and *ABCA6* was significantly upregulated on day 14. So far, its function is not described sufficiently; however, it is supposed to be involved in the lipid transport and homeostasis of macrophages due to its cholesterol-responsive regulation [54]. Another member of the ABC transporter family, ATP-binding cassette sub-family A member 1, has been described as a mediator of cortisol and dexamethasone transport [55]. This physiological ability is also conceivable for ATP-binding cassette sub-family A member 6 considering the presence of dexamethasone in the differentiation medium.

*PDE1A*, short for calcium/calmodulin-dependent 3′,5′-cyclic nucleotide phosphodiesterase 1A, was also upregulated [56]. According to literature, it is responsible for signal transduction and ion and calmodulin binding. Calmodulin, a multifunctional calcium-binding messenger protein, takes part in the differentiation of osteoblasts by regulating bone morphogenetic protein-2 (BMP-2) signaling, BMP-2 being a known osteogenic differentiation factor [57]. In a previous study, *PDE1A* was upregulated in human PDLSCs cultivated in osteogenic differentiation medium during matrix maturation, therefore concluding that genes related to calcium binding might be vital for the differentiation of stem cells into osteoblasts [58].

If binding functions are attributed to proteins by PANTHER, these can interact specifically with other molecules or selectively occupy binding sites. This is the case, for example, with the insulin-like growth factor-binding protein 2, which was highly expressed in this study. A publication on mesenchymal stromal cells revealed that the expression of insulin-like growth factor 2 (*IGF2*), insulin-like growth factor-binding protein 2 (*IGFBP2*), and integrin alpha5 (*ITGA5*) was upregulated during induced osteogenic differentiation [59]. The hypothesis of Hamidouche et al. [59] was that dexamethasone from the differentiation medium induced *ITGA5* expression, leading to an upregulated production of insulin-like growth factor 2 and insulin-like growth factor-binding protein 2 and finally triggering osteoblast gene expression, increasing mRNA levels of *RUNX2*, *ALP*, and *COL1A1*. Because this coincides with the findings of this study where a higher expression of the gene *IGFBP2* was seen on day 14, it is possible that a similar crosstalk between the mentioned molecules can be observed in dental stem cell cultures.

The leptin receptor (*LEPR*) and the leptin receptor overlapping transcript (*LEPROT*) were also upregulated on day 14. While information on leptin receptors in the context of osteogenic differentiation of dental stem cells is rare, it was shown that mesenchymal stromal cells that express leptin receptors not only give rise to most of the new formed bone in adult bone marrow but are also the ones responsible for regeneration after a trauma [60]. Therefore, it allows the assumption that there is a link between the expression of leptin receptors in both DPSC and SCAP and the formation of mineralized matrix during induced osteogenic differentiation.

Interestingly, *ID3*, short for inhibitor of DNA-binding 3, was the only key gene found downregulated on day 14 of the microarray analysis. These findings align with the study of Peng et al. [61] which reported that at an early stage, bone morphogenetic proteins induce an overexpression of ID helix-loop-helix proteins needed for the proliferation of osteoblast progenitor cells. This is followed by an obligatory downregulation of ID proteins, a process that is crucial for the terminal differentiation of cells committed to the osteoblast lineage.

The serum amyloid A (SAA) proteins are a family of lipophilic molecules that are relevant during acute phase response, e.g., during infectious attack, and also responsible for the transport of high-density lipoproteins and cholesterol. Furthermore, they are suspected to influence tissue remodeling through their interaction with metalloproteinases. After synthesis in the liver, they circulate in the blood serum [62]. In this experiment, *SAA1*, *SAA2*, and *SAA4* were upregulated on day 14 during osteogenic differentiation. Ebert et al. [63] reported a similar observation, stating that SAA proteins hold the potential to induce mineralization in mesenchymal stem cells via Toll-like receptor 4 activation. They also promote the expression of proinflammatory cytokines such as IL-6, IL-8, interleukin 1 beta (IL-1β), C-X-C motif chemokine ligand 1 (CXCL1), and C-X-C motif chemokine ligand 2 (CXCL2).

*GPM6B*, a gene encoding for the membrane glycoprotein M6-b, showed high expression levels on day 14 of induced osteogenic differentiation. Its involvement in osteoblast differentiation and bone formation can be explained by its influence on the activity of alkaline phosphatase, whereby a reduced activity leads to a weaker mineralization of the extracellular matrix. The cytoskeleton organization, respectively the distribution of actin filaments and focal adhesions, seem to be influenced by *GPM6B* expression as well [64].

The genes *FAM107A* (actin-associated protein FAM107A), *PAPPA* (pappalysin-1), and *VWA5A* (von Willebrand factor A domain-containing protein 5A) that were also significantly upregulated on day 14 have not been described in the context of osteogenic differentiation yet. Further research is required to define their exact function during the mineralization process. So far, actin-associated protein FAM107A with its nuclear localization and coiled-coil domain is suspected to be a gene transcription and cell cycle regulator and a tumor suppressor gene [65]. Pappalysin-1 is known as a metalloproteinase, mainly investigated as a marker of acute coronary syndromes [66] or pathological birth disorders [67]. Interestingly, a reported function of pappalysin-1 is also the cleavage of the complex between insulin-like growth factor and insulin like growth factor binding protein [68]. *VWA5A*, also known as breast cancer suppressor candidate-1 (BCSC-1), is investigated as a tumor suppressor gene [69].

Overall, the gene expression profiling revealed extended insight into the regulation during induced osteogenic differentiation. The analysis of the transcriptome of DPSC and SCAP cultures showed many parallels, once again highlighting their shared evolutionary origin, leading to comparable gene expression patterns. *GPX3*, *PIP*, *IGFBP2*, *SAA2*/*SAA2-SAA4*/*SAA4*, *SAA1*, *GPM6B*, *FAM107A*, *LEPR*/*LEPROT*, *PAPPA*, *ABCA6*, *VWA5A*, *PDE1A*, and *ID3* appear to be crucial genes during mineralization processes. While some of them have already been described in the context of osteogenic differentiation for other stem cell types, further research is needed to explore the exact molecular functions of some of the genes.

## 4. Materials and Methods

### 4.1. Cell Isolation

Cells were isolated from both pulp tissue and the apical papilla of an extracted third molar of an 18-year-old patient with informed consent and according to a previously described protocol approved by an appropriate review board at the University of Regensburg [70]. The tooth was removed due to lack of space, was not impacted, and did not show carious decay or other pathological alterations. The primary cells were cultured in αMEM supplemented with 10% FBS, 50 μg/mL L-ascorbic acid 2-phosphate, 100 U/mL penicillin, and 100 μg/mL streptomycin at 37 °C with 5% CO_2_. All cell culture reagents were purchased from Gibco™ (Thermo Fisher Scientific, Waltham, MA, USA). The cells were used in the experiments with the same passages and at most in passage 3.

### 4.2. Stem Cell Characterization

Cells obtained from the pulp and apical papilla were analyzed for the expression of characteristic mesenchymal stem cell markers (CD73, CD90, and CD105) as suggested by the International Society for Cellular Therapy [25]. At the same time, cells with the expression of markers of different origin (CD34, CD45, CD11b, CD19, and HLA-DR) were excluded. Therefore, flow cytometric analysis was performed using a Human MSC Analysis Kit (BD Stemflow™, BD Bioscience, San Jose, CA, USA). Fluorescence was determined by FACSCanto™ (BD Bioscience, San Jose, CA, USA) on basis of at least 2 × 10^4^ events for each sample. Data from three independent experiments were collected (*n* = 3) and analyzed by FlowJo™ (BD Bioscience, San Jose, CA, USA).

Furthermore, the cells’ potential for multi-lineage differentiation was investigated. Pulp- and papilla-derived cells were incubated with adipogenic, chondrogenic, and osteogenic culture media (StemPro^®^ Adipogenesis, Osteogenesis and Chondrogenesis Differentiation Kit, Invitrogen Corporation, Carlsbad, CA, USA). Chondrogenic differentiation was evaluated by staining with Alcian Blue 8GX (Sigma-Aldrich, St. Louis, MO, USA) after 10 days, adipogenic differentiation with Oil Red O (Sigma-Aldrich, St. Louis, MO, USA), and osteogenic differentiation with Alizarin Red S (Carl Roth, Karlsruhe, Germany) after 21 days.

### 4.3. Cell Viability and Proliferation

DPSC and SCAP were cultured either with αMEM and 1% FBS or αMEM and 10% FBS in 96-well plates (4000 cells/well) and both cell viability and cell proliferation were determined after 1, 3, 5, and 7 days.

For the MTT assay, cells were then incubated with 100 µL/well of a 0.5 mg/mL MTT solution (Thiazolyl Blue Tetrazolium Bromide, Sigma-Aldrich, St. Louis, MO, USA) for 60 min at 37 °C and 5% CO_2_. Subsequently, the dye was dissolved in 200 µL/well of dimethyl sulfoxide (DMSO, Merck Millipore, Billerica) and optical density was measured on a microplate reader at λ = 570 nm (Infinite^®^ 200, Tecan, Männedorf, Switzerland). Cell number of DPSC and SCAP was determined by CyQUANT™ Cell Proliferation Assay (Life Technologies, Carlsbad, CA, USA) as described previously [37].

Median and 25–75% percentiles were calculated on basis of three independent experiments performed in triplicate (*n* = 9).

### 4.4. Cell Migration

To describe migration activity, a wound healing assay was performed. First, 70 µL of cell suspension (7 × 10^5^ cells/mL) was applied into each chamber of a 2-well silicone insert (Culture-Insert 2 Well in µ-Dish 35 mm, Ibidi, Gräfelfing, Germany) and incubated at 37 °C and 5% CO_2_ for 24 h. After removal of the silicone insert with sterile tweezers, the cell layer was covered with 2 mL of medium and cultivated for 72 h. 

The following groups were established for each cell type: (1) αMEM with 1% FBS, (2) αMEM with 10% FBS and (3) αMEM with 10% FBS and 20 μM Locostatin (Santa Cruz Biotechnology, CA, USA). Then, 8 µL of migration inhibitor Locostatin was added twice a day. 

The gap closure by migrating cell sheets was monitored by imaging of the same site of the standardized gaps in 12 h-intervals with a Zeiss Axio Lab.A1 microscope at 10× magnification (Zeiss, Jena, Germany). Images were edited with Fiji (National Institutes of Health, Bethesda, MD, USA) and the gap area was quantified. The wound healing assay was performed in triplicate and repeated twice (*n* = 12). Median values and 25–75% percentiles were normalized to the initial wound area, which was set to 100%.

### 4.5. Release of Signaling Molecules

Levels of biologically active proteins secreted by DPSC and SCAP during standard cell culture (αMEM and 10% FBS) and induced osteogenic differentiation (StemPro^®^ Osteogenesis Differentiation Kit, Invitrogen Corporation, Carlsbad, CA, USA) were quantified. Both cell types were cultured in 12-well plates (19,000 cells/well) for 3 weeks. Media were replaced regularly and stored frozen at −20 °C. The collected supernatants were pooled in the initial phase (days 1 to 8), the intermediate phase (days 9 to 15) and the late phase (days 16 to 21) of culture. Finally, the concentrations of TIMP, OPG, IL-6, IL-8, VEGF and TGF-β1 were determined by enzyme-linked immunosorbent assays (Quantikine^®^ ELISA Kit, R&D Systems, Minneapolis, MN, USA). Medians with 25–75% percentiles were calculated from three experiments performed in triplicates (*n* = 9).

### 4.6. Gene Expression Profiling

Transcriptome-wide gene expression of DPSC and SCAP during induced osteogenic differentiation was profiled by Clariom™ S Human Arrays (Applied Biosystems™ by Thermo Fisher Scientific Waltham, MA, USA), which covers over 20,000 well-annotated genes. 

Therefore, DPSC and SCAP were cultured in 12-well plates (19,000 cells/well) with either αMEM and 10% FBS or StemPro^®^ Osteogenesis Differentiation Kit (Invitrogen Corporation, Carlsbad, CA, USA). RNA was isolated (RNeasy^®^ Mini Kit, Qiagen, Hilden, Germany) on days 1 and 14 and quantified spectrophotometrically (NanoDrop™ 2000, Thermo Fisher Scientific, Waltham, MA, USA). Further sample processing was carried out by the Genomics Core Unit based at the University of Regensburg according to the Affymetrix GeneChip WT PLUS Reagent Kit instructions (Affymetrix, Santa Clara, CA, USA). To confirm the obtained results and increase data quality, the microarray experiment was performed in replicates and reproduced independently (*n* = 2). Raw data of all experiments can be accessed in the Supplementary File 2.

The Transcriptome Analysis Console (TAC) 4.0 Software (Applied Biosystems™ by Thermo Fisher Scientific Waltham, MA, USA) was used for comprehensive analysis of the microarray data and genes were ranked by the empirical Bayes method. Results of all replicates and repeats were analyzed collectively with the TAC software and the *p*-value and false discovery rate (FDR) were calculated according to the given algorithms. After data import, gene regulation (fold-change > 10; *p*-value ≤ 0.01) was evaluated in the context of various parameters such as timepoint, type of medium, and cell type. Group selections were made to identify similarities and differences. Finally, genes were submitted to PANTHER (version 16.0, released 2020/12/01), an organized database located in the Gene Ontology Consortium [71].

To validate the genetic profiling by the microarray, qRT-PCR was performed for selected genes using the TaqMan Fast Advanced Master Mix (Applied Biosystems, Thermo Fisher Scientific, Waltham, USA) and probes for following genes: *GPX3* (Hs00173566_m1), *PDE1A* (Hs00897273_m1), *SAA1*, *SAA2* (Hs00761940_s1), *IGFBP2* (Hs01040718_m1), *PAPPA* (Hs01029908_m1), *ABCA6* (Hs00979431_mH), *FAM107A* (Hs01100593_g1), *LEPR* (Hs00900252_g1), *PIP* (Hs00160082_m1), *VWA5A* (Hs00938346_g1), *ID3* (Hs00171409_m1), and the housekeeping genes *RPS18* (Hs99999901_s1), *ACTB* (Hs01060665_g1), *GAPDH* (Hs02786624_g1). Finally, results were normalized to the arithmetic mean of all housekeeping genes and related to day 1 by the comparative CT method (2^−ΔΔCT^) [72] to compute medians with 25–75% percentiles (*n* = 4).

### 4.7. Statistical Analysis

Data were not normally distributed and, therefore, analyzed nonparametrically at a significance level of α = 0.05. For situations with two unpaired groups, Mann–Whitney U-tests were performed and *p*-values were adjusted for multiple comparisons by the Holm-Šídák method (α = 0.05). In case of three or more unpaired groups, the Kruskal–Wallis test followed by Dunn’s multiple comparison test was applied. Statistically significant differences between the groups were indicated by equal lower-case letters in the respective figures. All statistical analyses were computed with GraphPad Prism 9 (GraphPad Software, La Jolla, CA, USA) and detailed record of all comparisons can be found in in Supplementary File 3.

## 5. Conclusions

Excluding parameters such as donor, age, passage, or culture conditions, this study shows that DPSC and SCAP not only share a common evolutionary origin, but also behave similarly in terms of viability, proliferation, migration, and regulation of gene expression. Based on the results of cytokine secretion, it can be assumed that SCAP can be isolated in a less differentiated state than DPSC. One possible explanation is their origin from the apical papilla, a tissue found only in immature teeth. However, both dental stem cell types seem equally suitable as reservoirs for regenerative procedures based on cell homing, which relies on migration into the root canal and differentiation into a mineralizing phenotype. Future studies on the identified regulatory genes may help to understand the exact molecular processes during osteogenic differentiation of dental stem cells.

## Figures and Tables

**Table 1:** Genes that were exclusively regulated in both cell types during induced osteogenic differentiation.

**Gene**	**Description**	**Fold Change**	***p*** **-Value**	**FDR**
*GPX3*	glutathione peroxidase 3	48.34	3.33 × 10^−8^	8.93 × 10^−5^
*PIP*	prolactin-induced protein	29.21	1.02 × 10^−5^	1.40 × 10^−3^
*IGFBP2*	insulin like growth factor binding protein 2	23.67	1.97 × 10^−7^	2.00 × 10^−4^
*SAA2*; *SAA2-SAA4*; *SAA4*	serum amyloid A2; SAA2-SAA4 readthrough; serum amyloid A4, constitutive	21.85	2.06 × 10^−7^	2.00 × 10^−4^
*SAA1*	serum amyloid A1	20.62	1.66 × 10^−6^	5.00 × 10^−4^
*GPM6B*	glycoprotein M6B	19.01	3.08 × 10^−6^	7.00 × 10^−5^
*FAM107A*	family with sequence similarity 107, member A	15.8	2.88 × 10^−6^	7.00 × 10^−4^
*LEPR*; *LEPROT*	leptin receptor; leptin receptor overlapping transcript	14.87	3.35 × 10^−5^	2.60 × 10^−3^
*PAPPA*	pregnancy-associated plasma protein A, pappalysin 1	14.22	5.79 × 10^−6^	1.00 × 10^−3^
*ABCA6*	ATP binding cassette subfamily A member 6	13.67	3.75 × 10^−5^	2.70 × 10^−3^
*VWA5A*	von Willebrand factor A domain containing 5A	12.06	9.66 × 10^−6^	1.40 × 10^−3^
*PDE1A*	phosphodiesterase 1A, calmodulin-dependent	10.26	6.00 × 10^−7^	3.00 × 10^−4^
*ID3*	inhibitor of DNA binding 3, dominant negative helix-loop-helix protein	−46.66	1.54 × 10^−6^	5.00 × 10^−4^

## Data Availability

The data presented in this study are available on request from the corresponding author.

## Supplementary Materials

The following supporting information can be downloaded at: https://www.mdpi.com/article/10.3390/ijms23052615/s1.
