# Supplementary material for: Molecular Biological Comparison of Dental Pulp- and Apical Papilla-Derived Stem Cells
Source: Int J Mol Sci. 2022 Feb 27;23(5):2615. doi: 10.3390/ijms23052615 (PMC8910327; doi:10.3390/ijms23052615)
Supplement: Supplementary file 1 [file ijms-23-02615-s001.zip › Supplementary File 1.pdf]

Supplementary File 1

Table S1 Genes specifically regulated during osteogenic differentiation in DPSC cultures.

Gene	Description	Fold change	*P*-Value	FDR
APOD	apolipoprotein D	152.11	3.11×10^{-7}	2.00×10^{-4}
IGFBP5	insulin like growth factor binding protein 5	62.44	4.64×10^{-8}	1.00×10^{-4}
GPX3	glutathione peroxidase 3	48.34	3.33×10^{-8}	8.93×10^{-5}
ADH1B	alcohol dehydrogenase 1B (class I), beta polypeptide	42.83	2.45×10^{-8}	7.51×10^{-5}
PIP	prolactin-induced protein	29.21	1.02×10^{-5}	1.40×10^{-3}
PPARGC1A	peroxisome proliferator-activated receptor gamma, coactivator 1 alpha	24.84	7.91×10^{-8}	1.00×10^{-4}
IGFBP2	insulin like growth factor binding protein 2	23.67	1.97×10^{-7}	2.00×10^{-4}
SAA2; SAA2-SAA4; SAA4	serum amyloid A2; SAA2-SAA4 readthrough; serum amyloid A4, constitutive	21.85	2.06×10^{-7}	2.00×10^{-4}
SAA1	serum amyloid A1	20.62	1.66×10^{-6}	5.00×10^{-4}
GPM6B	glycoprotein M6B	19.01	3.08×10^{-6}	7.00×10^{-4}
SLC14A1	solute carrier family 14 (urea transporter), member 1 (Kidd blood group)	16.22	1.74×10^{-5}	1.90×10^{-3}
SERPINA3	serpin peptidase inhibitor, clade A (alpha-1 antiproteinase, antitrypsin), member 3	15.91	1.00×10^{-4}	5.80×10^{-3}
SNED1	sushi, nidogen and EGF-like domains 1	15.86	1.22×10^{-5}	1.50×10^{-3}
FAM107A	family with sequence similarity 107, member A	15.8	2.88×10^{-6}	7.00×10^{-4}
CRABP2	cellular retinoic acid binding protein 2	15.3	3.29×10^{-5}	2.60×10^{-3}
NTN1	netrin 1	14.91	5.27×10^{-8}	1.00×10^{-4}

LEPR; LEPROT	leptin receptor; leptin receptor overlapping transcript	14.87	3.35×10^{-5}	2.60×10^{-3}
PAPPA	pregnancy-associated plasma protein A, pappalysin 1	14.22	5.79×10^{-6}	1.00×10^{-3}
BOC	BOC cell adhesion associated, oncogene regulated	14	7.29×10^{-7}	3.00×10^{-4}
ABCA6	ATP binding cassette subfamily A member 6	13.67	3.75×10^{-5}	2.70×10^{-3}
SCG2	secretogranin II	13.32	1.20×10^{-3}	2.21×10^{-2}
CHI3L2	chitinase 3-like 2	12.44	6.63×10^{-7}	3.00×10^{-4}
VWA5A	von Willebrand factor A domain containing 5A	12.06	9.66×10^{-6}	1.40×10^{-3}
CXCL12	chemokine (C-X-C motif) ligand 12	11.45	7.68×10^{-6}	1.20×10^{-3}
PDE1A	phosphodiesterase 1A, calmodulin-dependent	10.26	6.00×10^{-7}	3.00×10^{-4}
ASS1	argininosuccinate synthase 1	10.25	7.62×10^{-8}	1.00×10^{-4}
TXNIP	thioredoxin interacting protein	10.25	2.65×10^{-5}	2.40×10^{-3}
ID3	inhibitor of DNA binding 3, dominant negative helix-loop-helix protein	-46.66	1.54×10^{-6}	5.00×10^{-4}

Table S2 Genes specifically regulated during osteogenic differentiation in SCAP cultures.

Gene	Description	Fold change	*P*-Value	FDR
A2M	alpha-2-macroglobulin	107.36	1.91×10^{-7}	4.00×10^{-4}
DIO2	deiodinase, iodothyronine, type II	93.23	1.00×10^{-4}	9.10×10^{-3}
GPX3	glutathione peroxidase 3	72.61	1.33×10^{-8}	2.00×10^{-4}
C3	complement component 3	56.73	1.15×10^{-7}	4.00×10^{-4}
SAA2; SAA2-SAA4; SAA4	serum amyloid A2; SAA2-SAA4 readthrough; serum amyloid A4, constitutive	31.54	4.02×10^{-5}	5.30×10^{-3}
FMO1	flavin containing monooxygenase 1	30.97	3.85×10^{-8}	2.00×10^{-4}
SAA1	serum amyloid A1	29.91	3.25×10^{-5}	4.60×10^{-3}
FAM107A	family with sequence similarity 107, member A	24.85	1.36×10^{-5}	2.90×10^{-3}
CFH	complement factor H	22.75	5.35×10^{-6}	1.90×10^{-3}
IGFBP2	insulin like growth factor binding protein 2	22.54	1.41×10^{-6}	1.10×10^{-3}
LEPR; LEPROT	leptin receptor; leptin receptor overlapping transcript	20.63	1.87×10^{-6}	1.30×10^{-3}
ST8SIA1	ST8 alpha-N-acetyl-neuraminide alpha-2,8-sialyltransferase 1	17.33	3.25×10^{-6}	1.60×10^{-3}
SLC2A12	solute carrier family 2 (facilitated glucose transporter), member 12	17.01	6.79×10^{-6}	2.10×10^{-3}
SPRY1	sprouty RTK signaling antagonist 1	15.95	1.94×10^{-5}	3.50×10^{-3}
GPM6B	glycoprotein M6B	15.71	1.96×10^{-6}	1.30×10^{-3}
ABCA6	ATP binding cassette subfamily A member 6	15.5	2.48×10^{-5}	4.10×10^{-3}
VWA5A	von Willebrand factor A domain containing 5A	15.09	1.23×10^{-5}	2.80×10^{-3}
SERPING1	serpin peptidase inhibitor, clade G (C1 inhibitor), member 1	14.72	1.00×10^{-4}	1.01×10^{-2}

PAPPA	pregnancy-associated plasma protein A, pappalysin 1	14.35	4.25×10^{-6}	1.80×10^{-3}
GMNC	geminin coiled-coil domain containing	14.3	4.00×10^{-4}	1.88×10^{-2}
PTK2B	protein tyrosine kinase 2 beta	13.87	1.66×10^{-6}	1.20×10^{-3}
PDE1A	phosphodiesterase 1A, calmodulin-dependent	12.64	9.09×10^{-6}	2.40×10^{-3}
CD14	CD14 molecule	12.34	7.44×10^{-7}	8.00×10^{-4}
RGS2	regulator of G-protein signaling 2	12.28	3.05×10^{-3}	5.98×10^{-2}
PIP	prolactin-induced protein	12.24	5.84×10^{-6}	2.00×10^{-3}
FMO2	flavin containing monooxygenase 2	12.23	2.81×10^{-6}	1.60×10^{-3}
PDGFD	platelet derived growth factor D	12.08	3.81×10^{-7}	6.00×10^{-4}
MAOA	monoamine oxidase A	11.77	4.00×10^{-6}	1.80×10^{-3}
DPT	dermatopontin	10.91	9.32×10^{-5}	8.80×10^{-3}
SAMHD1	SAM domain and HD domain 1	10.8	3.15×10^{-6}	1.60×10^{-3}
PZP	pregnancy-zone protein	10.73	1.98×10^{-5}	3.50×10^{-3}
BRINP1	bone morphogenetic protein/retinoic acid inducible neural-specific 1	10.58	1.97×10^{-5}	3.50×10^{-3}
JAM2	junctional adhesion molecule 2	10.43	1.00×10^{-4}	1.01×10^{-2}
ID3	inhibitor of DNA binding 3, dominant negative helix-loop-helix protein	-20.51	1.63×10^{-5}	3.10×10^{-3}
