# Supplementary material for: Molecular Biological Comparison of Dental Pulp- and Apical Papilla-Derived Stem Cells
Source: Int J Mol Sci. 2022 Feb 27;23(5):2615. doi: 10.3390/ijms23052615 (PMC8910327; doi:10.3390/ijms23052615)
Supplement: Supplementary file 1 [file ijms-23-02615-s001.zip › Supplementary File 2/206/QC/P206_QC Report.PDF]

Signal Histogram - SST-RMA-GENE-FULL - Group 1 - (2)

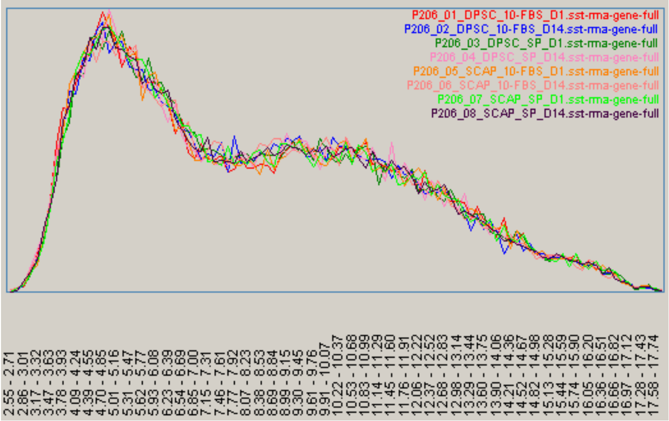

Log Probe Cell Intensity - (3)

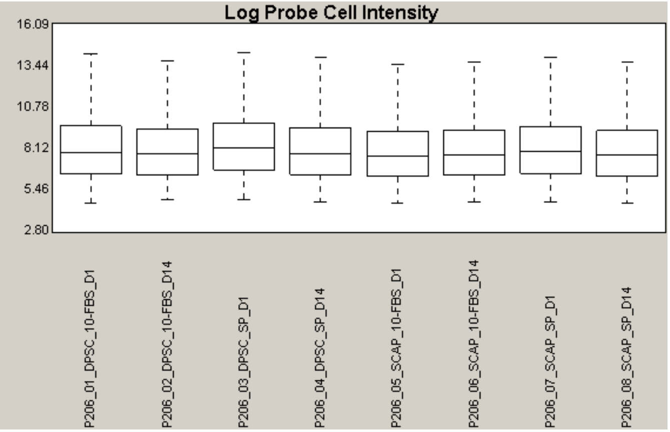

Relative Log Probe Cell Intensity - (4)

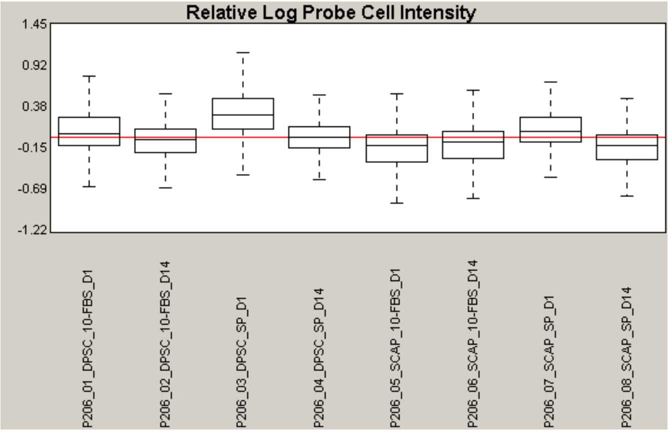

Log Expression Signal - SST-RMA-GENE-FULL - Group 1 - (5)

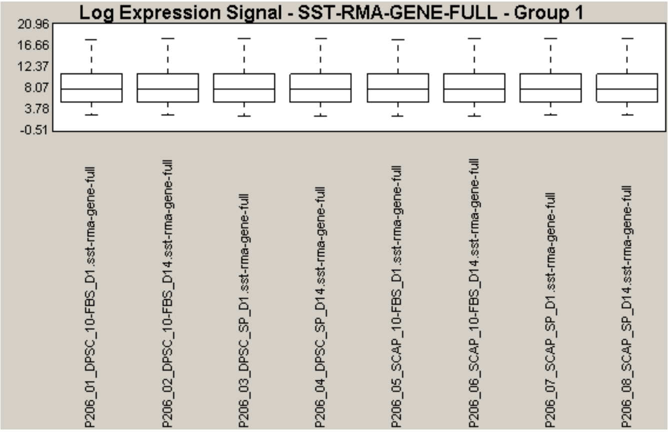

Relative Log Expression Signal - SST-RMA-GENE-FULL - Group 1 - (6)

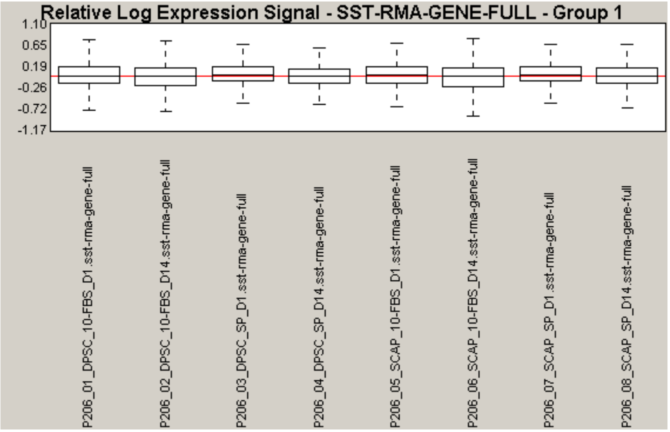

Pearson's Correlation (signal) - (7)

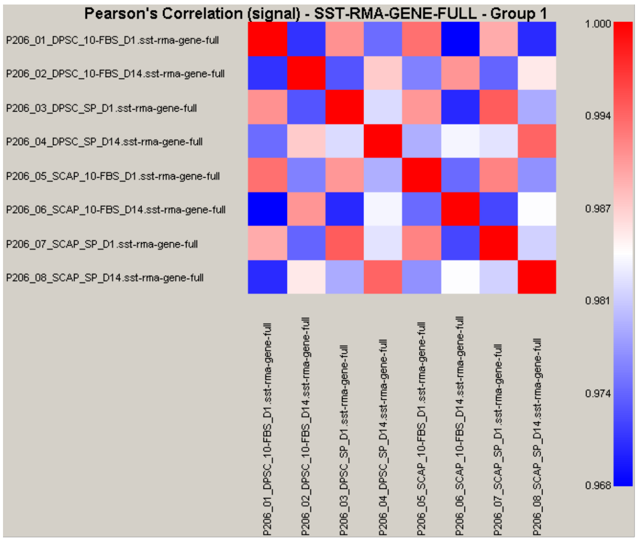

Spearman Rank Correlation (signal) - (8)

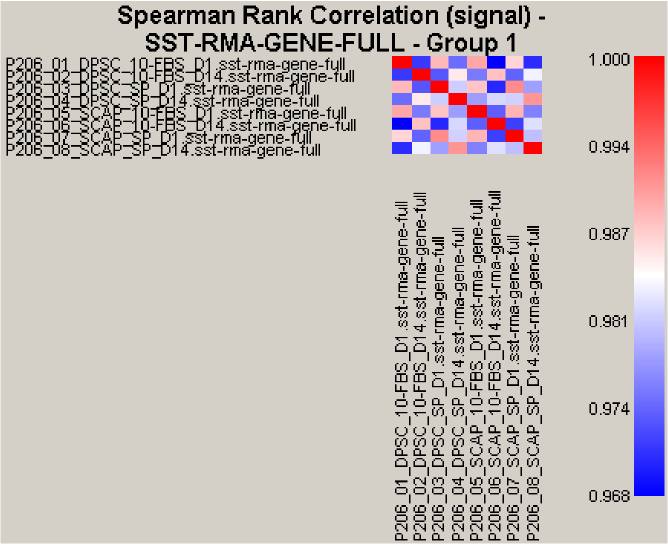

(1) =
(2) = File
(3) = Threshold Test
(4) = Scale
(5) = organism
(6) = user
(7) = KFB ID

(1)	(2)	(3)	(4)	(5)	(6)	(7)
	P206_01_DPSC_10-FBS_D1.CEL			human	Jutta	1
	P206_02_DPSC_10-FBS_D14.CEL			human	Jutta	2
	P206_03_DPSC_SP_D1.CEL			human	Jutta	3
	P206_04_DPSC_SP_D14.CEL			human	Jutta	4
	P206_05_SCAP_10-FBS_D1.CEL			human	Jutta	5
	P206_06_SCAP_10-FBS_D14.CEL			human	Jutta	6
	P206_07_SCAP_SP_D1.CEL			human	Jutta	7
	P206_08_SCAP_SP_D14.CEL			human	Jutta	8
	P206_01_DPSC_10-FBS_D1.sst-rma-gene-full.chp	Within Bounds	log2	human	Jutta	1
	P206_02_DPSC_10-FBS_D14.sst-rma-gene-full.chp	Within Bounds	log2	human	Jutta	2
	P206_03_DPSC_SP_D1.sst-rma-gene-full.chp	Within Bounds	log2	human	Jutta	3
	P206_04_DPSC_SP_D14.sst-rma-gene-full.chp	Within Bounds	log2	human	Jutta	4
	P206_05_SCAP_10-FBS_D1.sst-rma-gene-full.chp	Within Bounds	log2	human	Jutta	5
	P206_06_SCAP_10-FBS_D14.sst-rma-gene-full.chp	Within Bounds	log2	human	Jutta	6
	P206_07_SCAP_SP_D1.sst-rma-gene-full.chp	Within Bounds	log2	human	Jutta	7
	P206_08_SCAP_SP_D14.sst-rma-gene-full.chp	Within Bounds	log2	human	Jutta	8

(1) = Threshold Test
(2) = apt-free-mem
(3) = apt-opt-probe-count
(4) = apt-opt-cc-expr-chp-out-dir
(5) = apt-opt-qc-groups-file
(6) = apt-opt-temp-dir
(7) = apt-opt-use-disk
(8) = apt-opt-diskCache
(9) = apt-opt-set-analysis-name
(10) = apt-opt-do-residuals
(11) = apt-opt-do-feature-effects
(12) = apt-opt-write-sketch
(13) = apt-opt-write-profile
(14) = bgrd_mean
(15) = pm_mean
(16) = all_probeset_probesets
(17) = all_probeset_atoms
(18) = all_probeset_mean
(19) = all_probeset_stdev
(20) = all_probeset_mad_residual_mean
(21) = all_probeset_mad_residual_stdev
(22) = all_probeset_rle_mean
(23) = all_probeset_rle_stdev
(24) = control->affx->bac_spike_probesets
(25) = control->affx->bac_spike_atoms
(26) = control->affx->bac_spike_mean
(27) = control->affx->bac_spike_stdev
(28) = control->affx->bac_spike_mad_residual_mean
(29) = control->affx->bac_spike_mad_residual_stdev
(30) = control->affx->bac_spike_rle_mean
(31) = control->affx->bac_spike_rle_stdev
(32) = control->affx->ercc->step_probesets
(33) = control->affx->ercc->step_atoms
(34) = control->affx->ercc->step_mean
(35) = control->affx->ercc->step_stdev
(36) = control->affx->ercc->step_mad_residual_mean
(37) = control->affx->ercc->step_mad_residual_stdev
(38) = control->affx->ercc->step_rle_mean
(39) = control->affx->ercc->step_rle_stdev
(40) = control->affx->polya_spike_probesets
(41) = control->affx->polya_spike_atoms
(42) = control->affx->polya_spike_mean
(43) = control->affx->polya_spike_stdev
(44) = control->affx->polya_spike_mad_residual_mean
(45) = control->affx->polya_spike_mad_residual_stdev
(46) = control->affx->polya_spike_rle_mean
(47) = control->affx->polya_spike_rle_stdev
(48) = control->bgp->antigenomic_probesets
(49) = control->bgp->antigenomic_atoms
(50) = control->bgp->antigenomic_mean
(51) = control->bgp->antigenomic_stdev
(52) = control->bgp->antigenomic_mad_residual_mean
(53) = control->bgp->antigenomic_mad_residual_stdev
(54) = control->bgp->antigenomic_rle_mean
(55) = control->bgp->antigenomic_rle_stdev
(56) = neg_control_probesets
(57) = neg_control_atoms
(58) = neg_control_mean
(59) = neg_control_stdev

(60) = neg_control_mad_residual_mean
(61) = neg_control_mad_residual_stdev
(62) = neg_control_rle_mean
(63) = neg_control_rle_stdev
(64) = pos_control_probesets
(65) = pos_control_atoms
(66) = pos_control_mean
(67) = pos_control_stdev
(68) = pos_control_mad_residual_mean
(69) = pos_control_mad_residual_stdev
(70) = pos_control_rle_mean
(71) = pos_control_rle_stdev
(72) = pos_vs_neg_auc
(73) = control->affx->polya_spike-AFFX-r2-Bs-dap-5_st
(74) = control->affx->polya_spike-AFFX-r2-Bs-lys-5_st
(75) = control->affx->polya_spike-AFFX-r2-Bs-phe-5_st
(76) = control->affx->polya_spike-AFFX-r2-Bs-thr-5_s_st
(77) = control->affx->bac_spike-AFFX-r2-Ec-bioB-5_at
(78) = control->affx->bac_spike-AFFX-r2-Ec-bioC-5_at
(79) = control->affx->bac_spike-AFFX-r2-Ec-bioD-5_at
(80) = control->affx->bac_spike-AFFX-r2-P1-cre-5_at
(81) = control->affx->ercc->step-ERCCmix1step11
(82) = control->affx->ercc->step-ERCCmix1step12
(83) = control->affx->ercc->step-ERCCmix1step13
(84) = control->affx->ercc->step-ERCCmix1step14
(85) = control->affx->ercc->step-ERCCmix1step15
(86) = control->affx->ercc->step-ERCCmix1step16
(87) = control->affx->ercc->step-ERCCmix1step17
(88) = control->affx->ercc->step-ERCCmix1step18
(89) = control->affx->ercc->step-ERCCmix1step19
(90) = control->affx->ercc->step-ERCCmix1step20
(91) = control->affx->ercc->step-ERCCmix2step14
(92) = control->affx->ercc->step-ERCCmix2step15
(93) = control->affx->ercc->step-ERCCmix2step16
(94) = control->affx->ercc->step-ERCCmix2step17
(95) = control->affx->ercc->step-ERCCmix2step18
(96) = control->affx->ercc->step-ERCCmix2step19
(97) = control->affx->ercc->step-ERCCmix2step20
(98) = control->affx->ercc->step-ERCCmix2step21
(99) = control->affx->ercc->step-ERCCmix2step22
(100) = control->affx->ercc->step-ERCCmix2step23
(101) = control->affx->ercc->step-ERCCmix2step24
(102) = control->affx->ercc->step-ERCCmix2step25
(103) = control->affx->ercc->step-ERCCmix2step26
(104) = control->affx->ercc->step-ERCCmix2step27
(105) = control->affx->ercc->step-ERCCmix2step28
(106) = control->affx->ercc->step-ERCCmix2step29
(107) = control->affx->ercc->step-ERCCmix2step30
(108) = control->affx->ercc->step-ERCCmix2step31
(109) = control->affx->ercc->step-ERCCmix2step32
(110) = control->affx->ercc->step-ERCCmix2step33

	(1)	(2)	(3)	(4)
P206_01_DPSC_10-FBS_D1.sst-rma-gene-full	Within Bounds	5347650355	300304	C:\Command_Console\Data\Default [Default]\P206\45247
P206_02_DPSC_10-FBS_D14.sst-rma-gene-full	Within Bounds	5347650355	300304	C:\Command_Console\Data\Default [Default]\P206\45247
P206_03_DPSC_SP_D1.sst-rma-gene-full	Within Bounds	5347650355	300304	C:\Command_Console\Data\Default [Default]\P206\45247
P206_04_DPSC_SP_D14.sst-rma-gene-full	Within Bounds	5347650355	300304	C:\Command_Console\Data\Default [Default]\P206\45247
P206_05_SCAP_10-FBS_D1.sst-rma-gene-full	Within Bounds	5347650355	300304	C:\Command_Console\Data\Default [Default]\P206\45247

	(1)	(2)	(3)	(4)
P206_06_SCAP_10-FBS_D14.sst-rma-gene-full	Within Bounds	5347650355	300304	C:\Command_Console\Data\Default [Default]\P206\45247
P206_07_SCAP_SP_D1.sst-rma-gene-full	Within Bounds	5347650355	300304	C:\Command_Console\Data\Default [Default]\P206\45247
P206_08_SCAP_SP_D14.sst-rma-gene-full	Within Bounds	5347650355	300304	C:\Command_Console\Data\Default [Default]\P206\45247

	(5)	(6)	(7)	(8)
P206_01_DPSC_10-FBS_D1.sst-rma-gene-full	Clariom_S_Human.r1.qcc	C:/Command_Console/Data/Default [Default]/P206/temp	true	50
P206_02_DPSC_10-FBS_D14.sst-rma-gene-full	Clariom_S_Human.r1.qcc	C:/Command_Console/Data/Default [Default]/P206/temp	true	50
P206_03_DPSC_SP_D1.sst-rma-gene-full	Clariom_S_Human.r1.qcc	C:/Command_Console/Data/Default [Default]/P206/temp	true	50
P206_04_DPSC_SP_D14.sst-rma-gene-full	Clariom_S_Human.r1.qcc	C:/Command_Console/Data/Default [Default]/P206/temp	true	50
P206_05_SCAP_10-FBS_D1.sst-rma-gene-full	Clariom_S_Human.r1.qcc	C:/Command_Console/Data/Default [Default]/P206/temp	true	50
P206_06_SCAP_10-FBS_D14.sst-rma-gene-full	Clariom_S_Human.r1.qcc	C:/Command_Console/Data/Default [Default]/P206/temp	true	50
P206_07_SCAP_SP_D1.sst-rma-gene-full	Clariom_S_Human.r1.qcc	C:/Command_Console/Data/Default [Default]/P206/temp	true	50
P206_08_SCAP_SP_D14.sst-rma-gene-full	Clariom_S_Human.r1.qcc	C:/Command_Console/Data/Default [Default]/P206/temp	true	50

	(9)	(10)	(11)	(12)	(13)	(14)	(15)	(16)	(17)	(18)
P206_01_DPSC_10-FBS_D1.sst-rma-gene-full	sst-rma-gene-full	false	false	false	false	276.802094	890.120911	24351	247934	8.332061
P206_02_DPSC_10-FBS_D14.sst-rma-gene-full	sst-rma-gene-full	false	false	false	false	296.702057	765.387451	24351	247934	8.317271
P206_03_DPSC_SP_D1.sst-rma-gene-full	sst-rma-gene-full	false	false	false	false	344.940399	948.669067	24351	247934	8.335001
P206_04_DPSC_SP_D14.sst-rma-gene-full	sst-rma-gene-full	false	false	false	false	282.572144	808.505310	24351	247934	8.318723
P206_05_SCAP_10-FBS_D1.sst-rma-gene-full	sst-rma-gene-full	false	false	false	false	263.085114	726.378357	24351	247934	8.325740
P206_06_SCAP_10-FBS_D14.sst-rma-gene-full	sst-rma-gene-full	false	false	false	false	296.464142	735.219482	24351	247934	8.312253
P206_07_SCAP_SP_D1.sst-rma-gene-full	sst-rma-gene-full	false	false	false	false	303.369598	844.824829	24351	247934	8.328299
P206_08_SCAP_SP_D14.sst-rma-gene-full	sst-rma-gene-full	false	false	false	false	269.895660	734.699097	24351	247934	8.319884

	(19)	(20)	(21)	(22)	(23)	(24)	(25)	(26)	(27)	(28)
P206_01_DPSC_10-FBS_D1.sst-rma-gene-full	3.387360	0.254658	0.187807	0.293636	0.367430	4	44	11.308765	1.835421	0.078368
P206_02_DPSC_10-FBS_D14.sst-rma-gene-full	3.337362	0.267264	0.192123	0.299676	0.398257	4	44	11.612987	1.793796	0.031331
P206_03_DPSC_SP_D1.sst-rma-gene-full	3.340436	0.240878	0.177536	0.241895	0.293838	4	44	11.440630	1.800424	0.055863
P206_04_DPSC_SP_D14.sst-rma-gene-full	3.364436	0.243821	0.176845	0.242167	0.321206	4	44	11.469018	1.820179	0.040879
P206_05_SCAP_10-FBS_D1.sst-rma-gene-full	3.378384	0.275967	0.193851	0.252184	0.288731	4	44	11.510306	1.738103	0.044450
P206_06_SCAP_10-FBS_D14.sst-rma-gene-full	3.324186	0.289935	0.203680	0.327165	0.432395	4	44	11.766126	1.802399	0.059178
P206_07_SCAP_SP_D1.sst-rma-gene-full	3.354733	0.247191	0.179556	0.233637	0.272388	4	44	11.524157	1.823908	0.051636
P206_08_SCAP_SP_D14.sst-rma-gene-full	3.341706	0.262864	0.195687	0.263917	0.350542	4	44	11.584795	1.802715	0.056615

	(29)	(30)	(31)	(32)	(33)	(34)	(35)	(36)	(37)	(38)
P206_01_DPSC_10-FBS_D1.sst-rma-gene-full	0.071562	0.219615	0.046134	30	900	5.862583	0.934941	0.231893	0.065062	0.090094
P206_02_DPSC_10-FBS_D14.sst-rma-gene-full	0.022707	0.084607	0.048078	30	900	5.974239	0.934775	0.214720	0.060875	0.089427
P206_03_DPSC_SP_D1.sst-rma-gene-full	0.025256	0.087750	0.022091	30	900	6.033405	0.907093	0.247307	0.077599	0.137986
P206_04_DPSC_SP_D14.sst-rma-gene-full	0.025945	0.059362	0.061497	30	900	5.784829	0.963089	0.224591	0.075539	0.133503
P206_05_SCAP_10-FBS_D1.sst-rma-gene-full	0.014888	0.039057	0.041373	30	900	6.065913	0.886263	0.238080	0.072601	0.165751
P206_06_SCAP_10-FBS_D14.sst-rma-gene-full	0.033606	0.237746	0.037243	30	900	5.829530	0.941404	0.238910	0.069666	0.082171
P206_07_SCAP_SP_D1.sst-rma-gene-full	0.037541	0.023657	0.022873	30	900	5.941903	0.964009	0.230156	0.071568	0.074249
P206_08_SCAP_SP_D14.sst-rma-gene-full	0.031649	0.056415	0.045884	30	900	5.709468	0.980408	0.236634	0.077327	0.195785

	(39)	(40)	(41)	(42)	(43)	(44)	(45)	(46)	(47)	(48)	(49)
P206_01_DPSC_10-FBS_D1.sst-rma-gene-full	0.070194	4	44	8.818623	1.558880	0.096604	0.051028	0.113826	0.079406	3	2901
P206_02_DPSC_10-FBS_D14.sst-rma-gene-full	0.065884	4	44	9.164112	1.491663	0.101904	0.070134	0.231664	0.094108	3	2901
P206_03_DPSC_SP_D1.sst-rma-gene-full	0.078447	4	44	8.864247	1.678874	0.164038	0.109232	0.081650	0.094625	3	2901
P206_04_DPSC_SP_D14.sst-rma-gene-full	0.105468	4	44	8.997782	1.632973	0.114929	0.062539	0.085050	0.111184	3	2901
P206_05_SCAP_10-FBS_D1.sst-rma-gene-full	0.111869	4	44	8.941098	1.558218	0.099398	0.080171	0.036531	0.026221	3	2901
P206_06_SCAP_10-FBS_D14.sst-rma-gene-full	0.070555	4	44	8.954324	1.642321	0.105187	0.070056	0.063512	0.038054	3	2901
P206_07_SCAP_SP_D1.sst-rma-gene-full	0.065569	4	44	8.939342	1.435423	0.131820	0.045276	0.157976	0.128179	3	2901
P206_08_SCAP_SP_D14.sst-rma-gene-full	0.118126	4	44	8.974417	1.633012	0.177742	0.094846	0.063438	0.036161	3	2901

	(50)	(51)	(52)	(53)	(54)	(55)	(56)	(57)	(58)
P206_01_DPSC_10-FBS_D1.sst-rma-gene-full	3.410163	0.202957	0.244923	0.048878	0.007231	0.002626	612	2092	5.067997
P206_02_DPSC_10-FBS_D14.sst-rma-gene-full	3.434287	0.196129	0.244011	0.034464	0.016892	0.008811	612	2092	4.959925
P206_03_DPSC_SP_D1.sst-rma-gene-full	3.391523	0.211205	0.238796	0.067292	0.025872	0.010510	612	2092	5.078648

	(50)	(51)	(52)	(53)	(54)	(55)	(56)	(57)	(58)
P206_04_DPSC_SP_D14.sst-rma-gene-full	3.403918	0.204667	0.247251	0.046708	0.013476	0.010471	612	2092	4.958632
P206_05_SCAP_10-FBS_D1.sst-rma-gene-full	3.493670	0.252092	0.278643	0.047334	0.076276	0.047826	612	2092	5.006500
P206_06_SCAP_10-FBS_D14.sst-rma-gene-full	3.443906	0.197782	0.281003	0.068529	0.026512	0.018642	612	2092	4.912125
P206_07_SCAP_SP_D1.sst-rma-gene-full	3.409524	0.197136	0.249650	0.069561	0.007871	0.009155	612	2092	5.056756
P206_08_SCAP_SP_D14.sst-rma-gene-full	3.424639	0.210623	0.270798	0.052950	0.007244	0.008077	612	2092	5.007844

	(59)	(60)	(61)	(62)	(63)	(64)	(65)	(66)	(67)
P206_01_DPSC_10-FBS_D1.sst-rma-gene-full	1.544534	0.407198	0.453931	0.529859	0.692990	2197	15572	12.755950	2.683973
P206_02_DPSC_10-FBS_D14.sst-rma-gene-full	1.524212	0.400777	0.388778	0.493365	0.669535	2197	15572	12.444741	2.797210
P206_03_DPSC_SP_D1.sst-rma-gene-full	1.651534	0.386664	0.439881	0.442848	0.552486	2197	15572	12.683640	2.742659
P206_04_DPSC_SP_D14.sst-rma-gene-full	1.591743	0.384163	0.393427	0.458903	0.511810	2197	15572	12.516929	2.743277
P206_05_SCAP_10-FBS_D1.sst-rma-gene-full	1.639720	0.434105	0.409894	0.495560	0.535303	2197	15572	12.687367	2.704493
P206_06_SCAP_10-FBS_D14.sst-rma-gene-full	1.550525	0.417357	0.417909	0.489674	0.595812	2197	15572	12.439163	2.766625
P206_07_SCAP_SP_D1.sst-rma-gene-full	1.614886	0.387947	0.423798	0.450118	0.536029	2197	15572	12.704011	2.721913
P206_08_SCAP_SP_D14.sst-rma-gene-full	1.643995	0.443507	0.460531	0.496179	0.559247	2197	15572	12.466448	2.738301

	(68)	(69)	(70)	(71)	(72)	(73)	(74)	(75)	(76)
P206_01_DPSC_10-FBS_D1.sst-rma-gene-full	0.110104	0.130819	0.248947	0.234558	0.981803	10.717468	7.100158	8.130388	9.326476
P206_02_DPSC_10-FBS_D14.sst-rma-gene-full	0.107513	0.133399	0.229827	0.242532	0.978594	10.928107	7.473319	8.535046	9.719977
P206_03_DPSC_SP_D1.sst-rma-gene-full	0.096003	0.109034	0.186414	0.182978	0.978123	10.788437	6.906217	8.203341	9.558994
P206_04_DPSC_SP_D14.sst-rma-gene-full	0.098412	0.109314	0.167385	0.195828	0.979333	10.804324	7.084119	8.320465	9.782220
P206_05_SCAP_10-FBS_D1.sst-rma-gene-full	0.114711	0.150625	0.203776	0.211994	0.979447	10.788915	7.146946	8.344492	9.484039
P206_06_SCAP_10-FBS_D14.sst-rma-gene-full	0.124441	0.145444	0.246995	0.233558	0.979402	10.913595	7.067177	8.331325	9.505199
P206_07_SCAP_SP_D1.sst-rma-gene-full	0.104173	0.116624	0.186958	0.187722	0.978499	10.746721	7.453291	8.206521	9.350835
P206_08_SCAP_SP_D14.sst-rma-gene-full	0.108059	0.130955	0.223987	0.262250	0.977093	10.906894	7.151887	8.234587	9.604299

	(77)	(78)	(79)	(80)	(81)	(82)	(83)	(84)
P206_01_DPSC_10-FBS_D1.sst-rma-gene-full	9.223200	10.471712	12.143485	13.396662	8.447510	5.419580	5.153071	6.046692
P206_02_DPSC_10-FBS_D14.sst-rma-gene-full	9.524851	10.847191	12.476975	13.602931	8.656570	5.603733	5.378064	6.105814
P206_03_DPSC_SP_D1.sst-rma-gene-full	9.365843	10.652827	12.279476	13.464375	8.576216	5.848389	5.419839	6.217388
P206_04_DPSC_SP_D14.sst-rma-gene-full	9.434187	10.584379	12.314592	13.542912	8.521810	5.208422	5.230450	5.802951
P206_05_SCAP_10-FBS_D1.sst-rma-gene-full	9.518121	10.733151	12.322433	13.467520	8.635572	5.630301	5.429215	6.401437
P206_06_SCAP_10-FBS_D14.sst-rma-gene-full	9.690106	10.965357	12.631422	13.777618	8.582276	5.555591	5.287353	5.970335
P206_07_SCAP_SP_D1.sst-rma-gene-full	9.420395	10.736687	12.355614	13.583933	8.615266	5.650276	5.246465	5.953422
P206_08_SCAP_SP_D14.sst-rma-gene-full	9.536135	10.741799	12.455793	13.605453	8.527894	5.300807	5.078573	5.857270

	(85)	(86)	(87)	(88)	(89)	(90)	(91)	(92)	(93)
P206_01_DPSC_10-FBS_D1.sst-rma-gene-full	5.751132	4.800951	5.757454	4.894288	6.719627	6.177144	5.442252	6.345291	6.101449
P206_02_DPSC_10-FBS_D14.sst-rma-gene-full	5.835039	5.155083	5.810648	5.115579	6.572343	5.892348	5.687288	6.333701	6.220110
P206_03_DPSC_SP_D1.sst-rma-gene-full	5.877135	5.017027	5.827473	5.310887	6.812875	6.145285	5.830479	6.406339	6.005274
P206_04_DPSC_SP_D14.sst-rma-gene-full	5.718848	4.956686	5.615749	5.011110	6.582394	5.763100	5.267527	6.277171	5.929528
P206_05_SCAP_10-FBS_D1.sst-rma-gene-full	5.786949	5.252104	5.791849	5.280334	6.795302	6.182203	5.977693	6.453987	6.273556
P206_06_SCAP_10-FBS_D14.sst-rma-gene-full	5.619621	4.813310	5.546703	5.131452	6.648830	5.868117	5.654336	6.227154	5.954011
P206_07_SCAP_SP_D1.sst-rma-gene-full	5.753105	4.863986	5.700584	5.055966	6.918026	5.993687	5.601890	6.380555	5.968711
P206_08_SCAP_SP_D14.sst-rma-gene-full	5.518899	4.809300	5.676143	4.872870	6.415794	5.868699	5.616138	6.173038	5.694425

	(94)	(95)	(96)	(97)	(98)	(99)	(100)	(101)	(102)
P206_01_DPSC_10-FBS_D1.sst-rma-gene-full	6.328980	6.072894	5.886157	6.500153	5.731158	4.979941	8.683738	4.692884	5.558382
P206_02_DPSC_10-FBS_D14.sst-rma-gene-full	6.343928	6.157963	5.974844	6.744659	5.740569	5.128470	8.954343	4.815769	5.817965
P206_03_DPSC_SP_D1.sst-rma-gene-full	6.460001	6.348268	6.164457	6.735979	5.790507	5.146323	8.660979	4.878108	6.024189
P206_04_DPSC_SP_D14.sst-rma-gene-full	5.924062	6.122016	5.635464	6.534825	5.731895	4.756691	8.860284	4.595687	5.674807
P206_05_SCAP_10-FBS_D1.sst-rma-gene-full	6.315854	6.401320	5.952718	6.748601	5.956811	5.272623	8.671988	5.005928	5.850926
P206_06_SCAP_10-FBS_D14.sst-rma-gene-full	6.169883	6.092894	5.653607	6.463438	5.639451	4.996251	8.710713	4.640036	5.735556
P206_07_SCAP_SP_D1.sst-rma-gene-full	6.204881	6.247054	6.198749	6.678473	5.948012	5.207363	8.789039	4.596682	5.816576
P206_08_SCAP_SP_D14.sst-rma-gene-full	5.938272	6.209340	5.463686	6.420013	5.782020	4.887384	8.693184	4.297190	5.477300

	(103)	(104)	(105)	(106)	(107)	(108)	(109)	(110)
P206_01_DPSC_10-FBS_D1.sst-rma-gene-full	6.137732	5.393569	6.195782	6.368305	4.733111	5.635509	5.114238	4.808528

	(103)	(104)	(105)	(106)	(107)	(108)	(109)	(110)
P206_02_DPSC_10-FBS_D14.sst-rma-gene-full	6.382671	5.606780	6.370485	6.229301	4.819241	5.745101	5.085158	4.943591
P206_03_DPSC_SP_D1.sst-rma-gene-full	6.435334	5.712628	6.421732	6.463314	4.814337	5.803395	5.083390	4.764595
P206_04_DPSC_SP_D14.sst-rma-gene-full	6.151698	5.329441	6.098971	6.216095	4.808950	5.640568	4.924214	4.653469
P206_05_SCAP_10-FBS_D1.sst-rma-gene-full	6.545506	5.582422	6.585692	6.309957	5.005066	5.758889	5.059491	5.063090
P206_06_SCAP_10-FBS_D14.sst-rma-gene-full	6.366227	5.191720	6.160884	6.239542	4.673555	5.481846	4.943405	4.867799
P206_07_SCAP_SP_D1.sst-rma-gene-full	6.440632	5.515450	6.302624	6.445589	4.715251	5.717247	4.896662	4.834855
P206_08_SCAP_SP_D14.sst-rma-gene-full	6.096640	5.234310	6.043307	6.167341	4.614027	5.384181	4.648592	4.517395
