# Supplementary material for: Molecular Biological Comparison of Dental Pulp- and Apical Papilla-Derived Stem Cells
Source: Int J Mol Sci. 2022 Feb 27;23(5):2615. doi: 10.3390/ijms23052615 (PMC8910327; doi:10.3390/ijms23052615)
Supplement: Supplementary file 1 [file ijms-23-02615-s001.zip › Supplementary File 2/229/QC/P229_QC Report.PDF]

Signal Histogram - SST-RMA-GENE-FULL - Group 1 - (2)

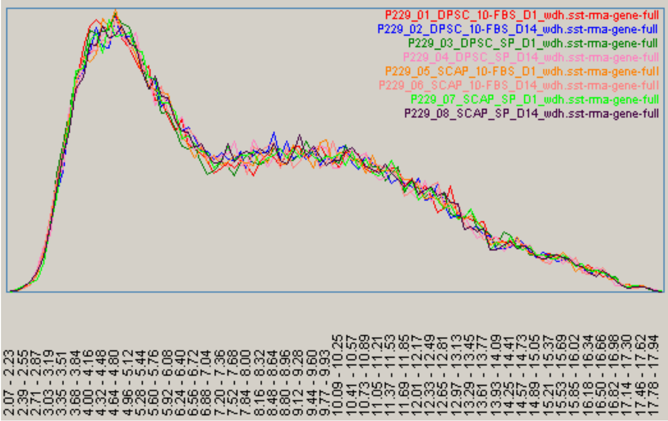

Log Probe Cell Intensity - (3)

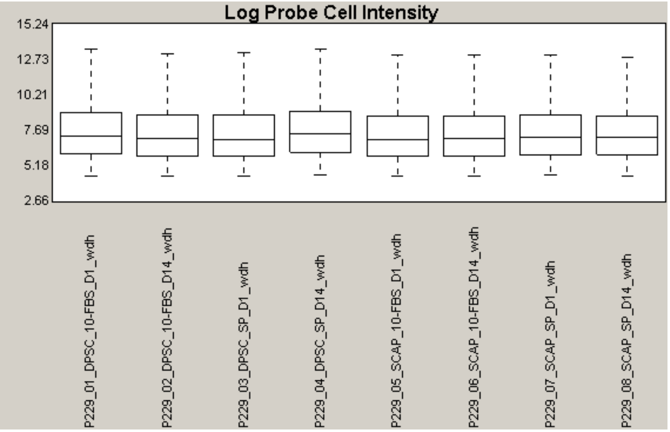

Relative Log Probe Cell Intensity - (4)

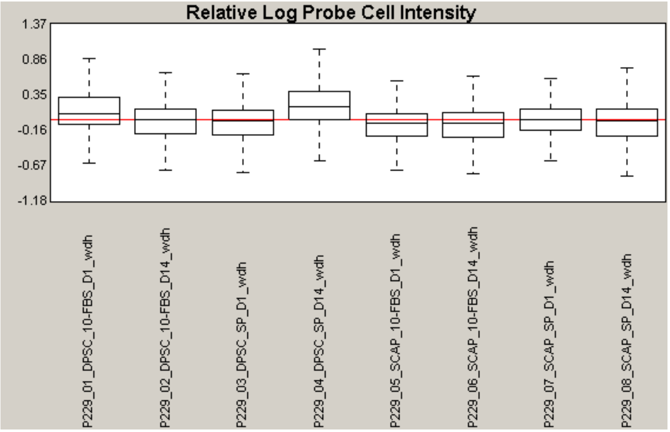

Log Expression Signal - SST-RMA-GENE-FULL - Group 1 - (5)

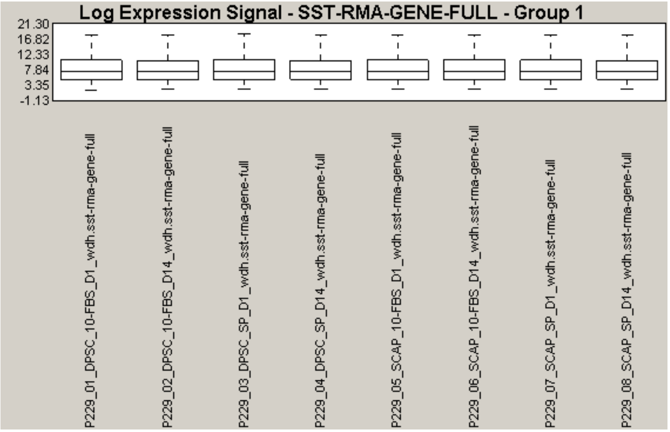

Relative Log Expression Signal - SST-RMA-GENE-FULL - Group 1 - (6)

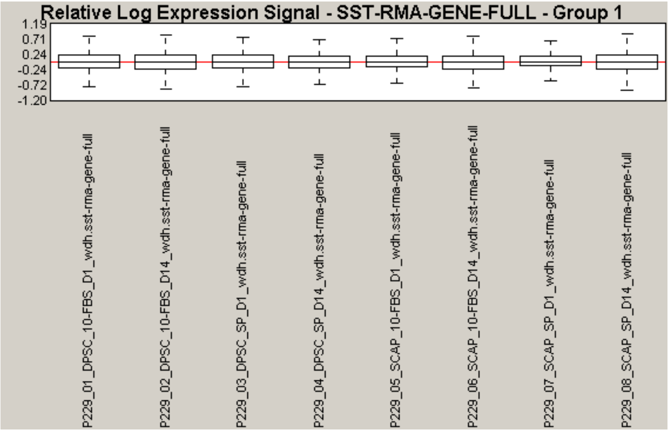

Pearson's Correlation (signal) - (7)

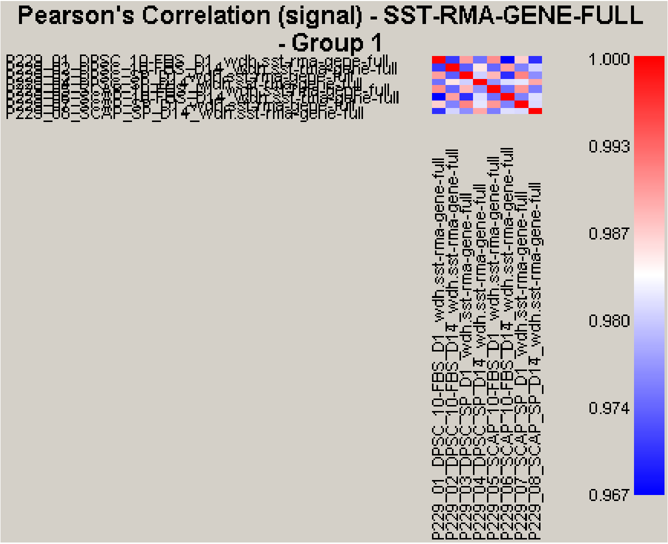

Spearman Rank Correlation (signal) - (8)

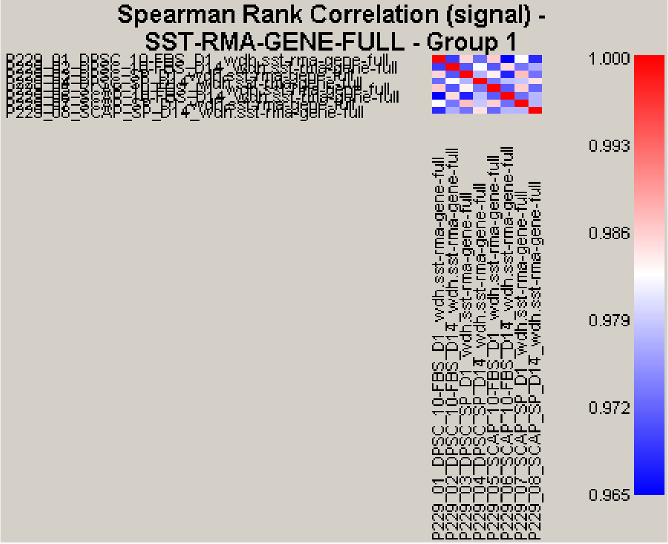

(1) =
(2) = File
(3) = Threshold Test
(4) = Scale
(5) = organism
(6) = user
(7) = KFB ID

(1)	(2)	(3)	(4)	(5)	(6)	(7)
	P229_01_DPSC_10-FBS_D1_wdh.CEL			human	Jutta	1
	P229_02_DPSC_10-FBS_D14_wdh.CEL			human	Jutta	2
	P229_03_DPSC_SP_D1_wdh.CEL			human	Jutta	3
	P229_04_DPSC_SP_D14_wdh.CEL			human	Jutta	4
	P229_05_SCAP_10-FBS_D1_wdh.CEL			human	Jutta	5
	P229_06_SCAP_10-FBS_D14_wdh.CEL			human	Jutta	6
	P229_07_SCAP_SP_D1_wdh.CEL			human	Jutta	7
	P229_08_SCAP_SP_D14_wdh.CEL			human	Jutta	8
	P229_01_DPSC_10-FBS_D1_wdh.sst-rma-gene-full.chp	Within Bounds	log2	human	Jutta	1
	P229_02_DPSC_10-FBS_D14_wdh.sst-rma-gene-full.chp	Within Bounds	log2	human	Jutta	2
	P229_03_DPSC_SP_D1_wdh.sst-rma-gene-full.chp	Within Bounds	log2	human	Jutta	3
	P229_04_DPSC_SP_D14_wdh.sst-rma-gene-full.chp	Within Bounds	log2	human	Jutta	4
	P229_05_SCAP_10-FBS_D1_wdh.sst-rma-gene-full.chp	Within Bounds	log2	human	Jutta	5
	P229_06_SCAP_10-FBS_D14_wdh.sst-rma-gene-full.chp	Within Bounds	log2	human	Jutta	6
	P229_07_SCAP_SP_D1_wdh.sst-rma-gene-full.chp	Within Bounds	log2	human	Jutta	7
	P229_08_SCAP_SP_D14_wdh.sst-rma-gene-full.chp	Outside Bounds	log2	human	Jutta	8

(1) = Threshold Test
(2) = apt-free-mem
(3) = apt-opt-probe-count
(4) = apt-opt-cc-expr-chp-out-dir
(5) = apt-opt-qc-groups-file
(6) = apt-opt-temp-dir
(7) = apt-opt-use-disk
(8) = apt-opt-diskCache
(9) = apt-opt-set-analysis-name
(10) = apt-opt-do-residuals
(11) = apt-opt-do-feature-effects
(12) = apt-opt-write-sketch
(13) = apt-opt-write-profile
(14) = bgrd_mean
(15) = pm_mean
(16) = all_probeset_probesets
(17) = all_probeset_atoms
(18) = all_probeset_mean
(19) = all_probeset_stdev
(20) = all_probeset_mad_residual_mean
(21) = all_probeset_mad_residual_stdev
(22) = all_probeset_rle_mean
(23) = all_probeset_rle_stdev
(24) = control->affx->bac_spike_probesets
(25) = control->affx->bac_spike_atoms
(26) = control->affx->bac_spike_mean
(27) = control->affx->bac_spike_stdev
(28) = control->affx->bac_spike_mad_residual_mean
(29) = control->affx->bac_spike_mad_residual_stdev
(30) = control->affx->bac_spike_rle_mean
(31) = control->affx->bac_spike_rle_stdev
(32) = control->affx->ercc->step_probesets
(33) = control->affx->ercc->step_atoms
(34) = control->affx->ercc->step_mean
(35) = control->affx->ercc->step_stdev
(36) = control->affx->ercc->step_mad_residual_mean
(37) = control->affx->ercc->step_mad_residual_stdev
(38) = control->affx->ercc->step_rle_mean
(39) = control->affx->ercc->step_rle_stdev
(40) = control->affx->polya_spike_probesets
(41) = control->affx->polya_spike_atoms
(42) = control->affx->polya_spike_mean
(43) = control->affx->polya_spike_stdev
(44) = control->affx->polya_spike_mad_residual_mean
(45) = control->affx->polya_spike_mad_residual_stdev
(46) = control->affx->polya_spike_rle_mean
(47) = control->affx->polya_spike_rle_stdev
(48) = control->bgp->antigenomic_probesets
(49) = control->bgp->antigenomic_atoms
(50) = control->bgp->antigenomic_mean
(51) = control->bgp->antigenomic_stdev
(52) = control->bgp->antigenomic_mad_residual_mean
(53) = control->bgp->antigenomic_mad_residual_stdev
(54) = control->bgp->antigenomic_rle_mean
(55) = control->bgp->antigenomic_rle_stdev
(56) = neg_control_probesets
(57) = neg_control_atoms
(58) = neg_control_mean
(59) = neg_control_stdev

(60) = neg_control_mad_residual_mean
(61) = neg_control_mad_residual_stdev
(62) = neg_control_rle_mean
(63) = neg_control_rle_stdev
(64) = pos_control_probesets
(65) = pos_control_atoms
(66) = pos_control_mean
(67) = pos_control_stdev
(68) = pos_control_mad_residual_mean
(69) = pos_control_mad_residual_stdev
(70) = pos_control_rle_mean
(71) = pos_control_rle_stdev
(72) = pos_vs_neg_auc
(73) = control->affx->polya_spike-AFFX-r2-Bs-dap-5_st
(74) = control->affx->polya_spike-AFFX-r2-Bs-lys-5_st
(75) = control->affx->polya_spike-AFFX-r2-Bs-phe-5_st
(76) = control->affx->polya_spike-AFFX-r2-Bs-thr-5_s_st
(77) = control->affx->bac_spike-AFFX-r2-Ec-bioB-5_at
(78) = control->affx->bac_spike-AFFX-r2-Ec-bioC-5_at
(79) = control->affx->bac_spike-AFFX-r2-Ec-bioD-5_at
(80) = control->affx->bac_spike-AFFX-r2-P1-cre-5_at
(81) = control->affx->ercc->step-ERCCmix1step11
(82) = control->affx->ercc->step-ERCCmix1step12
(83) = control->affx->ercc->step-ERCCmix1step13
(84) = control->affx->ercc->step-ERCCmix1step14
(85) = control->affx->ercc->step-ERCCmix1step15
(86) = control->affx->ercc->step-ERCCmix1step16
(87) = control->affx->ercc->step-ERCCmix1step17
(88) = control->affx->ercc->step-ERCCmix1step18
(89) = control->affx->ercc->step-ERCCmix1step19
(90) = control->affx->ercc->step-ERCCmix1step20
(91) = control->affx->ercc->step-ERCCmix2step14
(92) = control->affx->ercc->step-ERCCmix2step15
(93) = control->affx->ercc->step-ERCCmix2step16
(94) = control->affx->ercc->step-ERCCmix2step17
(95) = control->affx->ercc->step-ERCCmix2step18
(96) = control->affx->ercc->step-ERCCmix2step19
(97) = control->affx->ercc->step-ERCCmix2step20
(98) = control->affx->ercc->step-ERCCmix2step21
(99) = control->affx->ercc->step-ERCCmix2step22
(100) = control->affx->ercc->step-ERCCmix2step23
(101) = control->affx->ercc->step-ERCCmix2step24
(102) = control->affx->ercc->step-ERCCmix2step25
(103) = control->affx->ercc->step-ERCCmix2step26
(104) = control->affx->ercc->step-ERCCmix2step27
(105) = control->affx->ercc->step-ERCCmix2step28
(106) = control->affx->ercc->step-ERCCmix2step29
(107) = control->affx->ercc->step-ERCCmix2step30
(108) = control->affx->ercc->step-ERCCmix2step31
(109) = control->affx->ercc->step-ERCCmix2step32
(110) = control->affx->ercc->step-ERCCmix2step33

	(1)	(2)	(3)	(4)
P229_01_DPSC_10-FBS_D1_wdh.sst-rma-gene-full	Within Bounds	5446718668	300304	C:\Command_Console\Data\Default [Default]\P229\45246
P229_02_DPSC_10-FBS_D14_wdh.sst-rma-gene-full	Within Bounds	5446718668	300304	C:\Command_Console\Data\Default [Default]\P229\45246
P229_03_DPSC_SP_D1_wdh.sst-rma-gene-full	Within Bounds	5446718668	300304	C:\Command_Console\Data\Default [Default]\P229\45246
P229_04_DPSC_SP_D14_wdh.sst-rma-gene-full	Within Bounds	5446718668	300304	C:\Command_Console\Data\Default [Default]\P229\45246
P229_05_SCAP_10-FBS_D1_wdh.sst-rma-gene-full	Within Bounds	5446718668	300304	C:\Command_Console\Data\Default [Default]\P229\45246

	(1)	(2)	(3)	(4)
P229_06_SCAP_10-FBS_D14_wdh.sst-rma-gene-full	Within Bounds	5446718668	300304	C:\Command_Console\Data\Default [Default]\P229\45246
P229_07_SCAP_SP_D1_wdh.sst-rma-gene-full	Within Bounds	5446718668	300304	C:\Command_Console\Data\Default [Default]\P229\45246
P229_08_SCAP_SP_D14_wdh.sst-rma-gene-full	Outside Bounds	5446718668	300304	C:\Command_Console\Data\Default [Default]\P229\45246

	(5)	(6)	(7)	(8)
P229_01_DPSC_10-FBS_D1_wdh.sst-rma-gene-full	Clariom_S_Human.r1.qcc	C:/Command_Console/Data/Default [Default]/P229/temp	true	50
P229_02_DPSC_10-FBS_D14_wdh.sst-rma-gene-full	Clariom_S_Human.r1.qcc	C:/Command_Console/Data/Default [Default]/P229/temp	true	50
P229_03_DPSC_SP_D1_wdh.sst-rma-gene-full	Clariom_S_Human.r1.qcc	C:/Command_Console/Data/Default [Default]/P229/temp	true	50
P229_04_DPSC_SP_D14_wdh.sst-rma-gene-full	Clariom_S_Human.r1.qcc	C:/Command_Console/Data/Default [Default]/P229/temp	true	50
P229_05_SCAP_10-FBS_D1_wdh.sst-rma-gene-full	Clariom_S_Human.r1.qcc	C:/Command_Console/Data/Default [Default]/P229/temp	true	50
P229_06_SCAP_10-FBS_D14_wdh.sst-rma-gene-full	Clariom_S_Human.r1.qcc	C:/Command_Console/Data/Default [Default]/P229/temp	true	50
P229_07_SCAP_SP_D1_wdh.sst-rma-gene-full	Clariom_S_Human.r1.qcc	C:/Command_Console/Data/Default [Default]/P229/temp	true	50
P229_08_SCAP_SP_D14_wdh.sst-rma-gene-full	Clariom_S_Human.r1.qcc	C:/Command_Console/Data/Default [Default]/P229/temp	true	50

	(9)	(10)	(11)	(12)	(13)	(14)	(15)	(16)	(17)
P229_01_DPSC_10-FBS_D1_wdh.sst-rma-gene-full	sst-rma-gene-full	false	false	false	false	200.410553	648.489258	24351	247934
P229_02_DPSC_10-FBS_D14_wdh.sst-rma-gene-full	sst-rma-gene-full	false	false	false	false	210.976624	567.131348	24351	247934
P229_03_DPSC_SP_D1_wdh.sst-rma-gene-full	sst-rma-gene-full	false	false	false	false	191.245819	589.231812	24351	247934
P229_04_DPSC_SP_D14_wdh.sst-rma-gene-full	sst-rma-gene-full	false	false	false	false	245.080734	645.596008	24351	247934
P229_05_SCAP_10-FBS_D1_wdh.sst-rma-gene-full	sst-rma-gene-full	false	false	false	false	184.605087	552.167908	24351	247934
P229_06_SCAP_10-FBS_D14_wdh.sst-rma-gene-full	sst-rma-gene-full	false	false	false	false	197.550140	546.089600	24351	247934
P229_07_SCAP_SP_D1_wdh.sst-rma-gene-full	sst-rma-gene-full	false	false	false	false	189.283188	569.422302	24351	247934
P229_08_SCAP_SP_D14_wdh.sst-rma-gene-full	sst-rma-gene-full	false	false	false	false	184.763672	530.827332	24351	247934

	(18)	(19)	(20)	(21)	(22)	(23)	(24)	(25)	(26)
P229_01_DPSC_10-FBS_D1_wdh.sst-rma-gene-full	8.031298	3.455768	0.307696	0.218562	0.304438	0.376761	4	44	11.173335
P229_02_DPSC_10-FBS_D14_wdh.sst-rma-gene-full	8.022798	3.403405	0.316958	0.221485	0.319654	0.400282	4	44	11.391228
P229_03_DPSC_SP_D1_wdh.sst-rma-gene-full	8.028707	3.461118	0.308987	0.216633	0.279247	0.312451	4	44	11.265836
P229_04_DPSC_SP_D14_wdh.sst-rma-gene-full	8.023702	3.405574	0.302250	0.205678	0.271769	0.347179	4	44	11.244267
P229_05_SCAP_10-FBS_D1_wdh.sst-rma-gene-full	8.042243	3.432930	0.302759	0.219597	0.266312	0.326049	4	44	11.155105
P229_06_SCAP_10-FBS_D14_wdh.sst-rma-gene-full	8.019225	3.417125	0.322309	0.231246	0.327288	0.433581	4	44	11.206075
P229_07_SCAP_SP_D1_wdh.sst-rma-gene-full	8.034210	3.423068	0.291193	0.207575	0.234664	0.275061	4	44	11.137184
P229_08_SCAP_SP_D14_wdh.sst-rma-gene-full	8.034619	3.398777	0.354749	0.235592	0.314651	0.380287	4	44	11.124674

	(27)	(28)	(29)	(30)	(31)	(32)	(33)	(34)	(35)
P229_01_DPSC_10-FBS_D1_wdh.sst-rma-gene-full	1.903884	0.050436	0.014246	0.028754	0.022027	30	900	5.193991	0.939928
P229_02_DPSC_10-FBS_D14_wdh.sst-rma-gene-full	1.913336	0.065793	0.058971	0.192155	0.035932	30	900	5.216951	0.981433
P229_03_DPSC_SP_D1_wdh.sst-rma-gene-full	1.907436	0.062519	0.027834	0.066763	0.053856	30	900	5.236664	0.978909
P229_04_DPSC_SP_D14_wdh.sst-rma-gene-full	1.946018	0.050268	0.025917	0.051117	0.037157	30	900	5.244939	0.944343
P229_05_SCAP_10-FBS_D1_wdh.sst-rma-gene-full	1.929302	0.044042	0.025117	0.049891	0.036882	30	900	5.152750	0.953190
P229_06_SCAP_10-FBS_D14_wdh.sst-rma-gene-full	1.896236	0.064355	0.068030	0.021632	0.015194	30	900	5.178486	0.981376
P229_07_SCAP_SP_D1_wdh.sst-rma-gene-full	1.990965	0.077966	0.081828	0.061944	0.084177	30	900	5.197723	0.938013
P229_08_SCAP_SP_D14_wdh.sst-rma-gene-full	1.949455	0.075691	0.045882	0.074399	0.052344	30	900	5.099783	0.950731

	(36)	(37)	(38)	(39)	(40)	(41)	(42)	(43)	(44)
P229_01_DPSC_10-FBS_D1_wdh.sst-rma-gene-full	0.295146	0.081666	0.072758	0.080289	4	44	8.331974	1.584744	0.193849
P229_02_DPSC_10-FBS_D14_wdh.sst-rma-gene-full	0.274992	0.084764	0.077490	0.070005	4	44	8.444994	1.528540	0.165846
P229_03_DPSC_SP_D1_wdh.sst-rma-gene-full	0.278475	0.069814	0.092120	0.080027	4	44	8.516518	1.468247	0.135100
P229_04_DPSC_SP_D14_wdh.sst-rma-gene-full	0.256376	0.099537	0.096630	0.088745	4	44	8.336344	1.495912	0.153634
P229_05_SCAP_10-FBS_D1_wdh.sst-rma-gene-full	0.278504	0.100113	0.088638	0.084676	4	44	8.369872	1.478015	0.120418
P229_06_SCAP_10-FBS_D14_wdh.sst-rma-gene-full	0.266415	0.100696	0.065541	0.055193	4	44	8.414029	1.508685	0.126639
P229_07_SCAP_SP_D1_wdh.sst-rma-gene-full	0.258433	0.075719	0.063294	0.053960	4	44	8.241573	1.621609	0.202637
P229_08_SCAP_SP_D14_wdh.sst-rma-gene-full	0.296009	0.074485	0.095713	0.075793	4	44	8.016447	1.685454	0.164359

	(45)	(46)	(47)	(48)	(49)	(50)	(51)	(52)	(53)
P229_01_DPSC_10-FBS_D1_wdh.sst-rma-gene-full	0.209924	0.067802	0.086039	3	2901	3.168133	0.228697	0.315744	0.055383
P229_02_DPSC_10-FBS_D14_wdh.sst-rma-gene-full	0.062321	0.061899	0.086278	3	2901	3.196896	0.211191	0.314701	0.067334
P229_03_DPSC_SP_D1_wdh.sst-rma-gene-full	0.096013	0.133237	0.092825	3	2901	3.226232	0.235796	0.323231	0.040469

	(45)	(46)	(47)	(48)	(49)	(50)	(51)	(52)	(53)
P229_04_DPSC_SP_D14_wdh.sst-rma-gene-full	0.088773	0.047645	0.073436	3	2901	3.171983	0.211341	0.298695	0.044829
P229_05_SCAP_10-FBS_D1_wdh.sst-rma-gene-full	0.094208	0.013593	0.017432	3	2901	3.171741	0.230328	0.317160	0.089489
P229_06_SCAP_10-FBS_D14_wdh.sst-rma-gene-full	0.121423	0.030749	0.020545	3	2901	3.193437	0.208974	0.329000	0.059829
P229_07_SCAP_SP_D1_wdh.sst-rma-gene-full	0.172361	0.166848	0.181924	3	2901	3.160365	0.203269	0.301130	0.079834
P229_08_SCAP_SP_D14_wdh.sst-rma-gene-full	0.059750	0.366833	0.340939	3	2901	3.153503	0.204772	0.338485	0.078863

	(54)	(55)	(56)	(57)	(58)	(59)	(60)	(61)	(62)
P229_01_DPSC_10-FBS_D1_wdh.sst-rma-gene-full	0.008566	0.010118	612	2092	4.729986	1.624908	0.485694	0.476438	0.535350
P229_02_DPSC_10-FBS_D14_wdh.sst-rma-gene-full	0.020700	0.021009	612	2092	4.691423	1.629798	0.458437	0.487290	0.557171
P229_03_DPSC_SP_D1_wdh.sst-rma-gene-full	0.050036	0.025574	612	2092	4.786111	1.580941	0.471993	0.480781	0.534059
P229_04_DPSC_SP_D14_wdh.sst-rma-gene-full	0.009541	0.005175	612	2092	4.687297	1.669420	0.434028	0.434877	0.519017
P229_05_SCAP_10-FBS_D1_wdh.sst-rma-gene-full	0.008241	0.009039	612	2092	4.885017	1.676505	0.510906	0.523083	0.594448
P229_06_SCAP_10-FBS_D14_wdh.sst-rma-gene-full	0.017745	0.016061	612	2092	4.710873	1.606709	0.495771	0.501682	0.564019
P229_07_SCAP_SP_D1_wdh.sst-rma-gene-full	0.016337	0.021030	612	2092	4.823594	1.647326	0.473965	0.457948	0.523082
P229_08_SCAP_SP_D14_wdh.sst-rma-gene-full	0.022693	0.014250	612	2092	4.750488	1.692777	0.501078	0.489806	0.586332

	(63)	(64)	(65)	(66)	(67)	(68)	(69)	(70)	(71)
P229_01_DPSC_10-FBS_D1_wdh.sst-rma-gene-full	0.652804	2197	15572	12.519429	2.829505	0.134109	0.151935	0.237487	0.227441
P229_02_DPSC_10-FBS_D14_wdh.sst-rma-gene-full	0.580915	2197	15572	12.258124	2.906837	0.137283	0.165613	0.262394	0.258949
P229_03_DPSC_SP_D1_wdh.sst-rma-gene-full	0.584221	2197	15572	12.540343	2.828153	0.130068	0.147748	0.237571	0.244017
P229_04_DPSC_SP_D14_wdh.sst-rma-gene-full	0.635037	2197	15572	12.275087	2.905538	0.142068	0.150028	0.216698	0.248984
P229_05_SCAP_10-FBS_D1_wdh.sst-rma-gene-full	0.701456	2197	15572	12.501281	2.807130	0.128407	0.152874	0.214693	0.232326
P229_06_SCAP_10-FBS_D14_wdh.sst-rma-gene-full	0.644329	2197	15572	12.298950	2.892896	0.134689	0.155873	0.237531	0.243433
P229_07_SCAP_SP_D1_wdh.sst-rma-gene-full	0.585234	2197	15572	12.466064	2.834221	0.121446	0.135500	0.175136	0.191873
P229_08_SCAP_SP_D14_wdh.sst-rma-gene-full	0.682314	2197	15572	12.215147	2.894257	0.173874	0.210755	0.272097	0.289638

	(72)	(73)	(74)	(75)	(76)	(77)	(78)	(79)
P229_01_DPSC_10-FBS_D1_wdh.sst-rma-gene-full	0.978859	10.319584	6.488278	7.964993	8.555042	8.921227	10.468168	11.969208
P229_02_DPSC_10-FBS_D14_wdh.sst-rma-gene-full	0.975049	10.351634	6.684577	8.008379	8.735385	9.102810	10.735554	12.162575
P229_03_DPSC_SP_D1_wdh.sst-rma-gene-full	0.978428	10.342081	6.770583	8.272855	8.680552	8.968516	10.633508	12.042911
P229_04_DPSC_SP_D14_wdh.sst-rma-gene-full	0.974539	10.261160	6.684155	7.852579	8.547483	8.970869	10.484047	12.049295
P229_05_SCAP_10-FBS_D1_wdh.sst-rma-gene-full	0.977377	10.252546	6.681319	8.009119	8.536504	8.843312	10.507738	11.918852
P229_06_SCAP_10-FBS_D14_wdh.sst-rma-gene-full	0.975549	10.328379	6.685550	8.039364	8.602825	8.966262	10.530643	11.934026
P229_07_SCAP_SP_D1_wdh.sst-rma-gene-full	0.978364	10.195063	6.244506	8.059031	8.467692	8.761120	10.439510	11.954516
P229_08_SCAP_SP_D14_wdh.sst-rma-gene-full	0.974131	10.141340	6.018041	8.005484	7.900923	8.813199	10.402761	11.957144

	(80)	(81)	(82)	(83)	(84)	(85)	(86)	(87)
P229_01_DPSC_10-FBS_D1_wdh.sst-rma-gene-full	13.334736	7.912270	4.717139	4.828321	5.266117	5.033885	4.063106	4.678415
P229_02_DPSC_10-FBS_D14_wdh.sst-rma-gene-full	13.563974	7.991339	4.971649	4.890493	5.154758	4.831879	4.173756	4.902335
P229_03_DPSC_SP_D1_wdh.sst-rma-gene-full	13.418410	8.007588	4.933440	4.957421	5.232389	5.059353	4.177856	4.805133
P229_04_DPSC_SP_D14_wdh.sst-rma-gene-full	13.472858	7.793336	4.715837	5.040053	5.278286	5.022328	4.139285	4.667442
P229_05_SCAP_10-FBS_D1_wdh.sst-rma-gene-full	13.350517	7.891325	4.704627	4.806953	5.167249	4.981062	4.323564	4.610351
P229_06_SCAP_10-FBS_D14_wdh.sst-rma-gene-full	13.393368	7.904794	4.656056	5.077468	5.305281	4.986162	4.239289	4.589803
P229_07_SCAP_SP_D1_wdh.sst-rma-gene-full	13.393589	7.838210	4.748618	4.839834	5.260029	5.119345	4.265096	4.755816
P229_08_SCAP_SP_D14_wdh.sst-rma-gene-full	13.325592	7.818715	4.723442	4.640280	5.018881	4.868363	4.230798	4.682263

	(88)	(89)	(90)	(91)	(92)	(93)	(94)	(95)
P229_01_DPSC_10-FBS_D1_wdh.sst-rma-gene-full	4.423796	5.719356	5.253851	4.696534	5.315687	5.503456	5.344919	5.709029
P229_02_DPSC_10-FBS_D14_wdh.sst-rma-gene-full	4.314980	6.132867	5.457926	4.492991	5.299082	5.490861	5.373808	5.594839
P229_03_DPSC_SP_D1_wdh.sst-rma-gene-full	4.382736	5.733651	5.208847	4.546098	5.164858	5.349870	5.450436	5.699815
P229_04_DPSC_SP_D14_wdh.sst-rma-gene-full	4.429485	6.003716	5.393023	4.454493	5.368551	5.471405	5.581314	5.648735
P229_05_SCAP_10-FBS_D1_wdh.sst-rma-gene-full	4.208321	5.975446	5.228192	4.345177	5.336516	5.170847	5.459857	5.719188
P229_06_SCAP_10-FBS_D14_wdh.sst-rma-gene-full	4.454167	5.961914	5.223253	4.388998	5.129104	5.397487	5.376732	5.683897
P229_07_SCAP_SP_D1_wdh.sst-rma-gene-full	4.424839	5.887427	5.459271	4.520082	5.174328	5.230255	5.489815	5.548316
P229_08_SCAP_SP_D14_wdh.sst-rma-gene-full	4.375843	5.867615	5.216166	4.562976	5.224805	5.431121	5.341506	5.681136

	(96)	(97)	(98)	(99)	(100)	(101)	(102)	(103)
P229_01_DPSC_10-FBS_D1_wdh.sst-rma-gene-full	5.185305	5.782528	4.741985	4.367565	8.320807	4.466211	4.677923	5.385815

	(96)	(97)	(98)	(99)	(100)	(101)	(102)	(103)
P229_02_DPSC_10-FBS_D14_wdh.sst-rma-gene-full	5.143887	5.885349	4.878585	4.496886	8.428424	4.470758	4.849159	5.546087
P229_03_DPSC_SP_D1_wdh.sst-rma-gene-full	5.238819	5.909826	4.855405	4.483725	8.652571	4.712749	4.921020	5.639381
P229_04_DPSC_SP_D14_wdh.sst-rma-gene-full	5.196645	5.859459	4.929904	4.399532	8.286578	4.411617	4.995717	5.651444
P229_05_SCAP_10-FBS_D1_wdh.sst-rma-gene-full	5.269654	5.794528	4.745130	4.454545	8.278131	4.457500	4.783722	5.204990
P229_06_SCAP_10-FBS_D14_wdh.sst-rma-gene-full	5.182782	5.865764	4.885712	4.352956	8.375150	4.471730	4.674447	5.483583
P229_07_SCAP_SP_D1_wdh.sst-rma-gene-full	5.261582	5.743587	4.801604	4.412080	8.304475	4.533096	4.756375	5.569376
P229_08_SCAP_SP_D14_wdh.sst-rma-gene-full	4.943989	5.627464	4.748850	4.165470	8.192072	4.385978	4.681544	5.598600

	(104)	(105)	(106)	(107)	(108)	(109)	(110)
P229_01_DPSC_10-FBS_D1_wdh.sst-rma-gene-full	5.260991	5.471625	5.658463	4.206930	5.244722	4.170707	4.412269
P229_02_DPSC_10-FBS_D14_wdh.sst-rma-gene-full	4.981169	5.397827	5.652930	3.973805	5.389607	4.137996	4.202496
P229_03_DPSC_SP_D1_wdh.sst-rma-gene-full	4.984550	5.523964	5.482623	4.035535	5.384593	4.353450	4.212206
P229_04_DPSC_SP_D14_wdh.sst-rma-gene-full	4.870764	5.699566	5.993908	4.003875	5.218718	4.368299	4.454843
P229_05_SCAP_10-FBS_D1_wdh.sst-rma-gene-full	5.162698	5.337320	5.651463	4.181416	5.096297	4.145244	4.091177
P229_06_SCAP_10-FBS_D14_wdh.sst-rma-gene-full	4.810736	5.481543	5.772315	3.844901	5.460535	4.126072	4.191947
P229_07_SCAP_SP_D1_wdh.sst-rma-gene-full	4.936803	5.376875	5.797560	4.091868	5.432975	4.184791	4.167371
P229_08_SCAP_SP_D14_wdh.sst-rma-gene-full	4.800659	5.304696	5.518840	3.900036	5.230327	4.159046	4.051998
