# Supplementary material for: Molecular Biological Comparison of Dental Pulp- and Apical Papilla-Derived Stem Cells
Source: Int J Mol Sci. 2022 Feb 27;23(5):2615. doi: 10.3390/ijms23052615 (PMC8910327; doi:10.3390/ijms23052615)
Supplement: Supplementary file 1 [file ijms-23-02615-s001.zip › Supplementary File 3.pdf]

Supplementary File 3

Table S1 Viability, *p*-values of Kruskal-Wallis test followed by Dunn's multiple comparison test of different cell types and FBS concentrations against each other.

			DPSC	SCAP	
			10 % FBS	1 % FBS	10 % FBS
Day 1	DPSC	1 % FBS	0.0122	0.3492	0.1597
		10 % FBS		>0.9999	>0.9999
	SCAP	1 % FBS			>0.9999
Day 3	DPSC	1 % FBS	0.5194	>0.9999	>0.9999
		10 % FBS		0.4988	>0.9999
	SCAP	1 % FBS			>0.9999
Day 5	DPSC	1 % FBS	<0.0001	>0.9999	0.0002
		10 % FBS		<0.0001	>0.9999
	SCAP	1 % FBS			<0.0001
Day 7	DPSC	1 % FBS	0.0005	0.1657	0.0021
		10 % FBS		<0.0001	>0.9999
	SCAP	1 % FBS			<0.0001

Table S2 Proliferation, *p*-values of Kruskal-Wallis test followed by Dunn's multiple comparison test of different cell types and FBS concentrations against each other.

			DPSC	SCAP	
			10 % FBS	1 % FBS	10 % FBS
Day 1	DPSC	1 % FBS	>0.9999	>0.9999	>0.9999
		10 % FBS		>0.9999	>0.9999
	SCAP	1 % FBS			>0.9999
Day 3	DPSC	1 % FBS	>0.9999	>0.9999	>0.9999
		10 % FBS		>0.9999	>0.9999
	SCAP	1 % FBS			>0.9999
Day 5	DPSC	1 % FBS	0.0001	>0.9999	0.0009
		10 % FBS		<0.0001	>0.9999
	SCAP	1 % FBS			<0.0001
Day 7	DPSC	1 % FBS	<0.0001	0.4456	0.0030
		10 % FBS		<0.0001	>0.9999
	SCAP	1 % FBS			<0.0001

Table S3 Wound healing, *p*-values of Kruskal-Wallis test followed by Dunn's multiple comparison test of different cell types and established groups (αMEM with 1 % FBS, αMEM with 10 % FBS and αMEM with 10 % FBS and 20 μM Locostatin) against each other.

			DPSC		SCAP		
			10 % FBS	Locost.	1 % FBS	10 % FBS	Locost.
12h	DPSC	1 % FBS	0.0212	>0.9999	>0.9999	0.0082	>0.9999
		10 % FBS		0.1245	0.0023	>0.9999	<0.0001
		Locostatin			>0.9999	0.0470	0.0126
	SCAP	1 % FBS				0.0008	>0.9999
		10 % FBS					<0.0001
24h	DPSC	1 % FBS	<0.0001	>0.9999	>0.9999	0.0003	>0.9999
		10 % FBS		0.0002	<0.0001	>0.9999	<0.0001
		Locostatin			>0.9999	0.0070	0.1809
	SCAP	1 % FBS				0.0002	>0.9999
		10 % FBS					<0.0001
36h	DPSC	1 % FBS	<0.0001	>0.9999	>0.9999	<0.0001	>0.9999
		10 % FBS		<0.0001	<0.0001	>0.9999	<0.0001
		Locostatin			>0.9999	0.0006	0.3900
	SCAP	1 % FBS				0.0001	>0.9999
		10 % FBS					<0.0001
48h	DPSC	1 % FBS	<0.0001	>0.9999	>0.9999	<0.0001	>0.9999
		10 % FBS		<0.0001	<0.0001	>0.9999	<0.0001
		Locostatin			>0.9999	0.0010	0.1715
	SCAP	1 % FBS				0.0005	>0.9999
		10 % FBS					<0.0001
60h	DPSC	1 % FBS	<0.0001	>0.9999	>0.9999	<0.0001	>0.9999
		10 % FBS		<0.0001	<0.0001	>0.9999	<0.0001
		Locostatin			>0.9999	0.0005	0.5022
	SCAP	1 % FBS				0.0002	>0.9999
		10 % FBS					<0.0001
72h	DPSC	1 % FBS	<0.0001	>0.9999	>0.9999	<0.0001	>0.9999
		10 % FBS		<0.0001	<0.0001	>0.9999	<0.0001
		Locostatin			>0.9999	0.0002	>0.9999
	SCAP	1 % FBS				<0.0001	>0.9999
		10 % FBS					<0.0001

Table S4 Cytokines, *p*-values of Kruskal-Wallis test followed by Dunn's multiple comparison test of different cell types and culture media against each other.

TIMP			DPSC	SCAP	
			StemPro	10 % FBS	StemPro
Days 1 to 8	DPSC	10 % FBS	>0.9999	0.0026	<0.0001
		StemPro		0.1498	0.0056
	SCAP	10 % FBS			>0.9999
Days 9 to 15	DPSC	10 % FBS	0.5257	0.0038	0.5257
		StemPro		<0.0001	0.0038
	SCAP	10 % FBS			0.5257
Days 16 to 21	DPSC	10 % FBS	0.8101	0.0082	>0.9999
		StemPro		<0.0001	0.0331
	SCAP	10 % FBS			0.3279

OPG			DPSC	SCAP	
			StemPro	10 % FBS	StemPro
Days 1 to 8	DPSC	10 % FBS	0.8101	0.0026	<0.0001
		StemPro		0.2551	0.0056
	SCAP	10 % FBS			>0.9999
Days 9 to 15	DPSC	10 % FBS	0.8101	<0.0001	0.0056
		StemPro		0.0026	0.4173
	SCAP	10 % FBS			0.5257
Days 16 to 21	DPSC	10 % FBS	>0.9999	<0.0001	0.1965
		StemPro		0.0017	0.9911
	SCAP	10 % FBS			0.1498

IL8			DPSC	SCAP	
			StemPro	10 % FBS	StemPro
Days 1 to 8	DPSC	10 % FBS	0.5257	0.5257	0.0038
		StemPro		0.0038	0.5257
	SCAP	10 % FBS			<0.0001
Days 9 to 15	DPSC	10 % FBS	0.1130	>0.9999	0.0003
		StemPro		0.0331	0.5257
	SCAP	10 % FBS			<0.0001
Days 16 to 21	DPSC	10 % FBS	0.3258	>0.9999	0.0017
		StemPro		0.0080	0.5229
	SCAP	10 % FBS			<0.0001

IL6			DPSC	SCAP	
			StemPro	10 % FBS	StemPro
Days 1 to 8	DPSC	10 % FBS	0.0038	0.5257	<0.0001
		StemPro		0.5257	0.5257
	SCAP	10 % FBS			0.0038
Days 9 to 15	DPSC	10 % FBS	0.0026	0.5257	<0.0001
		StemPro		0.4173	0.8101
	SCAP	10 % FBS			0.0056
Days 16 to 21	DPSC	10 % FBS	0.0038	>0.9999	<0.0001
		StemPro		0.1965	>0.9999
	SCAP	10 % FBS			0.0038

VEGF			DPSC	SCAP	
			StemPro	10 % FBS	StemPro
Days 1 to 8	DPSC	10 % FBS	0.3279	0.0082	<0.0001
		StemPro		>0.9999	0.0082
	SCAP	10 % FBS			0.3279
Days 9 to 15	DPSC	10 % FBS	>0.9999	0.0118	<0.0001
		StemPro		0.2551	0.0011
	SCAP	10 % FBS			0.5257
Days 16 to 21	DPSC	10 % FBS	>0.9999	0.2551	0.0011
		StemPro		0.0118	<0.0001
	SCAP	10 % FBS			0.5257

TGF-β1			DPSC	SCAP	
			StemPro	10 % FBS	StemPro
Days 1 to 8	DPSC	10 % FBS	0.0624	0.0624	<0.0001
		StemPro		>0.9999	0.0624
	SCAP	10 % FBS			0.0624
Days 9 to 15	DPSC	10 % FBS	>0.9999	0.0038	0.0038
		StemPro		0.0038	0.0038
	SCAP	10 % FBS			>0.9999
Days 16 to 21	DPSC	10 % FBS	0.5257	0.1498	0.0844
		StemPro		0.0005	0.0002
	SCAP	10 % FBS			>0.9999

Table S5 Viability, *p*-values of Kruskal-Wallis test followed by Dunn's multiple comparison test of different time points against each other.

		DPSC			SCAP		
		Day 3	Day 5	Day 7	Day 3	Day 5	Day 7
1% FBS	Day 1	>0.9999	0.0157	<0.0001	0.4456	0.0027	<0.0001
	Day 3		0.3029	<0.0001		0.5039	<0.0001
	Day 5			0.0088			0.0375
10 % FBS	Day 1	0.4644	<0.0001	<0.0001	0.3607	<0.0001	<0.0001
	Day 3		0.0344	<0.0001		0.0498	<0.0001
	Day 5			0.0904			0.0858

Table S6 Proliferation, *p*-values of Kruskal-Wallis test followed by Dunn's multiple comparison test of different time points against each other.

		DPSC			SCAP		
		Day 3	Day 5	Day 7	Day 3	Day 5	Day 7
1% FBS	Day 1	0.0034	0.0002	<0.0001	<0.0001	0.0006	<0.0001
	Day 3		>0.9999	0.0751		>0.9999	>0.9999
	Day 5			0.4186			>0.9999
10 % FBS	Day 1	0.0172	<0.0001	<0.0001	0.0195	<0.0001	<0.0001
	Day 3		0.2709	0.0017		0.4365	0.0042
	Day 5			0.6148			0.6642

Table S7 Wound healing, *p*-values of Kruskal-Wallis test followed by Dunn's multiple comparison test of different time points against each other.

		12 h	24 h	36 h	48 h	60 h	72 h
DPSC 1% FBS	0 h	>0.9999	>0.9999	0.6343	0.0839	0.0474	0.0054
	12 h		>0.9999	>0.9999	>0.9999	>0.9999	0.5827
	24 h			>0.9999	>0.9999	>0.9999	>0.9999
	36 h				>0.9999	>0.9999	>0.9999
	48 h					>0.9999	>0.9999
	60 h						>0.9999
DPSC 10 % FBS	0 h	>0.9999	0.0106	<0.0001	<0.0001	<0.0001	<0.0001
	12 h		>0.9999	0.0060	<0.0001	<0.0001	<0.0001
	24 h			>0.9999	0.0284	0.0004	<0.0001
	36 h				>0.9999	0.2553	0.0094
	48 h					>0.9999	0.8345
	60 h						>0.9999
SCAP 1% FBS	0 h	>0.9999	>0.9999	0.1595	0.0218	0.0035	0.0004
	12 h		>0.9999	>0.9999	0.9555	0.2718	0.0559
	24 h			>0.9999	>0.9999	0.9555	0.2473
	36 h				>0.9999	>0.9999	>0.9999
	48 h					>0.9999	>0.9999
	60 h						>0.9999
SCAP 10 % FBS	0 h	>0.9999	0.0466	<0.0001	<0.0001	<0.0001	<0.0001
	12 h		>0.9999	0.0370	<0.0001	<0.0001	<0.0001
	24 h			>0.9999	0.0129	<0.0001	<0.0001
	36 h				>0.9999	0.0132	<0.0001
	48 h					>0.9999	0.0524
	60 h						>0.9999

Table S8 Cytokines, *p*-values of Kruskal-Wallis test followed by Dunn's multiple comparison test of different intervals against each other.

TIMP		DPSC		SCAP	
		Days 9 to 15	Days 16 to 21	Days 9 to 15	Days 16 to 21
10% FBS	Days 1 to 8	>0.9999	>0.9999	0.2665	0.2665
	Days 9 to 15		>0.9999		0.0020
StemPro	Days 1 to 8	0.0321	<0.0001	0.0478	<0.0001
	Days 9 to 15		0.2665		0.1414

OPG		DPSC		SCAP	
		Days 9 to 15	Days 16 to 21	Days 9 to 15	Days 16 to 21
10% FBS	Days 1 to 8	>0.9999	>0.9999	0.0699	>0.9999
	Days 9 to 15		>0.9999		0.0478
StemPro	Days 1 to 8	>0.9999	>0.9999	0.0212	0.0001
	Days 9 to 15		>0.9999		0.4686

IL8		DPSC		SCAP	
		Days 9 to 15	Days 16 to 21	Days 9 to 15	Days 16 to 21
10% FBS	Days 1 to 8	>0.9999	0.0054	>0.9999	0.0321
	Days 9 to 15		0.0478		0.2665
StemPro	Days 1 to 8	0.0699	<0.0001	0.0684	<0.0001
	Days 9 to 15		0.0699		0.0684

IL6		DPSC		SCAP	
		Days 9 to 15	Days 16 to 21	Days 9 to 15	Days 16 to 21
10% FBS	Days 1 to 8	>0.9999	0.1414	0.1414	0.7700
	Days 9 to 15		0.0699		0.0054
StemPro	Days 1 to 8	0.0033	0.0012	0.0002	0.0137
	Days 9 to 15		>0.9999		0.7700

VEGF		DPSC		SCAP	
		Days 9 to 15	Days 16 to 21	Days 9 to 15	Days 16 to 21
10% FBS	Days 1 to 8	>0.9999	>0.9999	>0.9999	0.4686
	Days 9 to 15		0.2665		0.1414
StemPro	Days 1 to 8	0.2665	<0.0001	0.0020	0.0020
	Days 9 to 15		0.0321		>0.9999

TGF-β1		DPSC		SCAP	
		Days 9 to 15	Days 16 to 21	Days 9 to 15	Days 16 to 21
10% FBS	Days 1 to 8	0.1414	0.0054	0.7700	0.7700
	Days 9 to 15		0.7700		0.0699
StemPro	Days 1 to 8	0.0699	<0.0001	0.0699	<0.0001
	Days 9 to 15		0.0699		0.0699

Table S9 Microarray validation by qRT-PCR, *p*-values of pairwise tests of different cell types against each other calculated by Mann-Whitney U-test adjusted for multiple comparisons by the Holm-Šídák method. All gene regulations at day 14 were significantly different from baseline ($p \leq 0.0236$).

StemPro	DPSC vs. SCAP
GPX3	0.0875
PDE1A	0.0236
SAA1, SAA2	0.5189
IGFBP2	0.0383
PAPPA	0.5189
ABCA6	0.0875
FAM107A	0.5189
LEPR	0.0875
PIP	0.0383
VWA5A	0.0383
ID3	0.0236
