# Supplementary figures and images for: Molecular Biological Comparison of Dental Pulp- and Apical Papilla-Derived Stem Cells
Source: Int J Mol Sci. 2022 Feb 27;23(5):2615. doi: 10.3390/ijms23052615 (PMC8910327; doi:10.3390/ijms23052615)

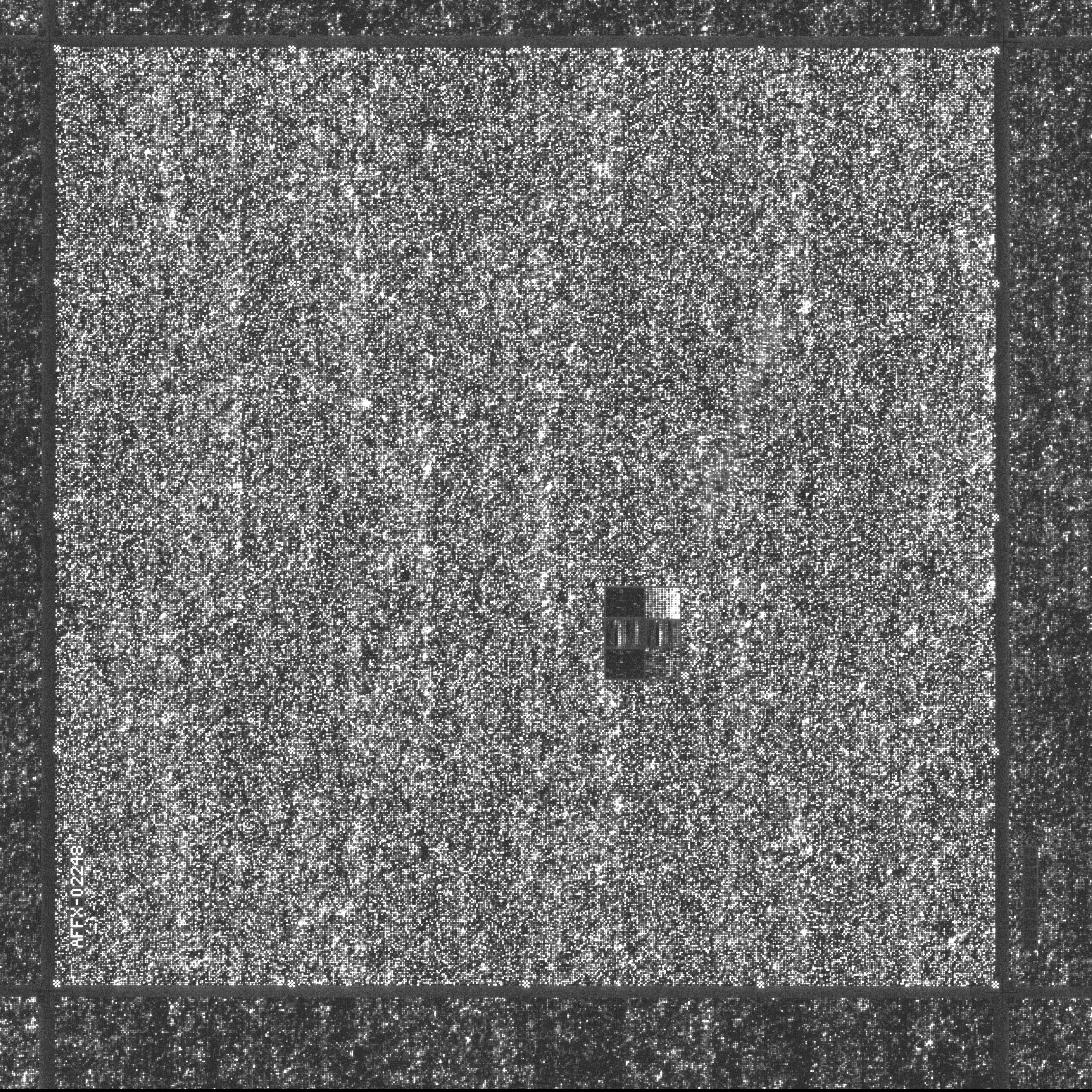

Supplement: Supplementary file 1 [file ijms-23-02615-s001.zip › Supplementary File 2/206/JPG files/P206_01_DPSC_10-FBS_D1.JPG]

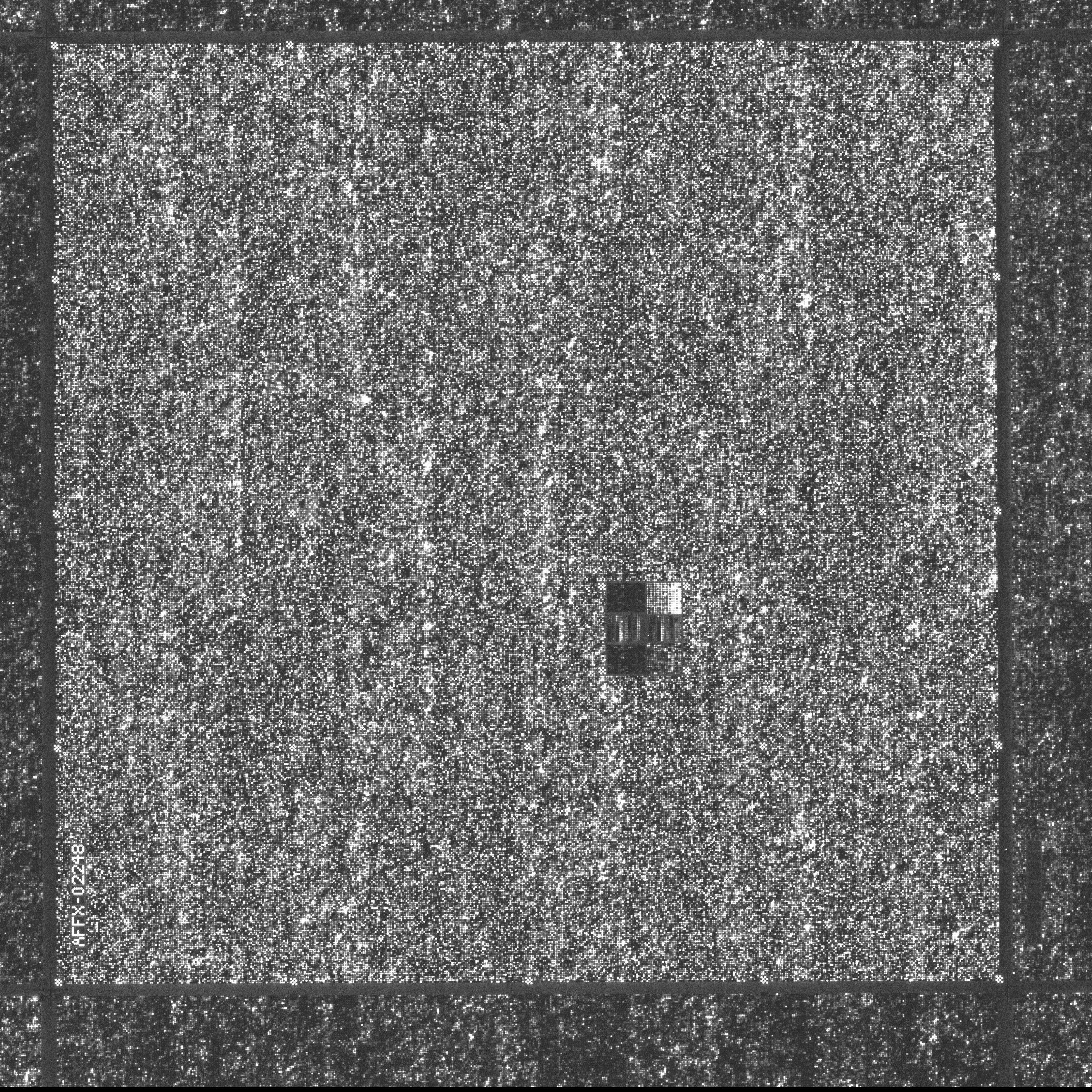

Supplement: Supplementary file 1 [file ijms-23-02615-s001.zip › Supplementary File 2/206/JPG files/P206_02_DPSC_10-FBS_D14.JPG]

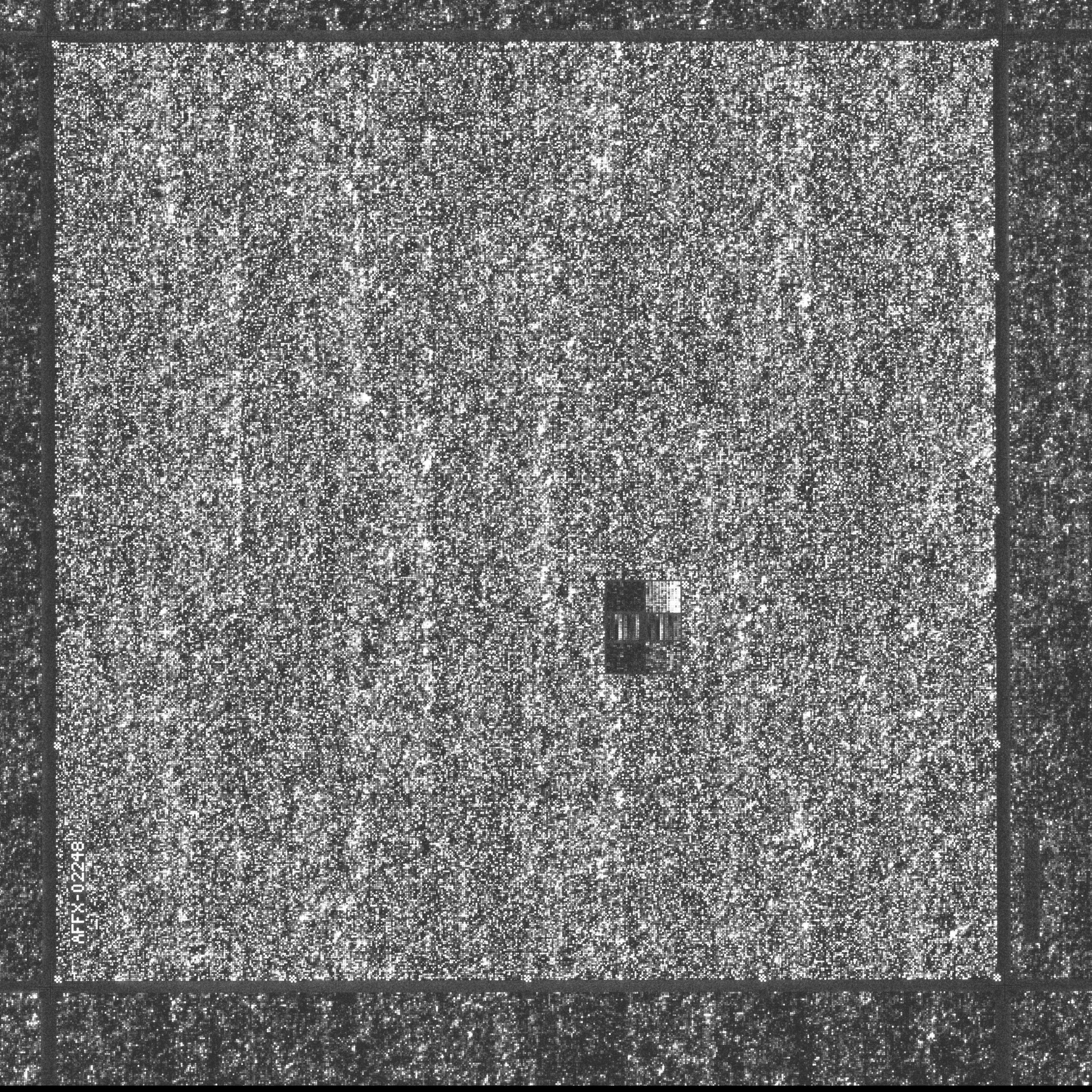

Supplement: Supplementary file 1 [file ijms-23-02615-s001.zip › Supplementary File 2/206/JPG files/P206_03_DPSC_SP_D1.JPG]

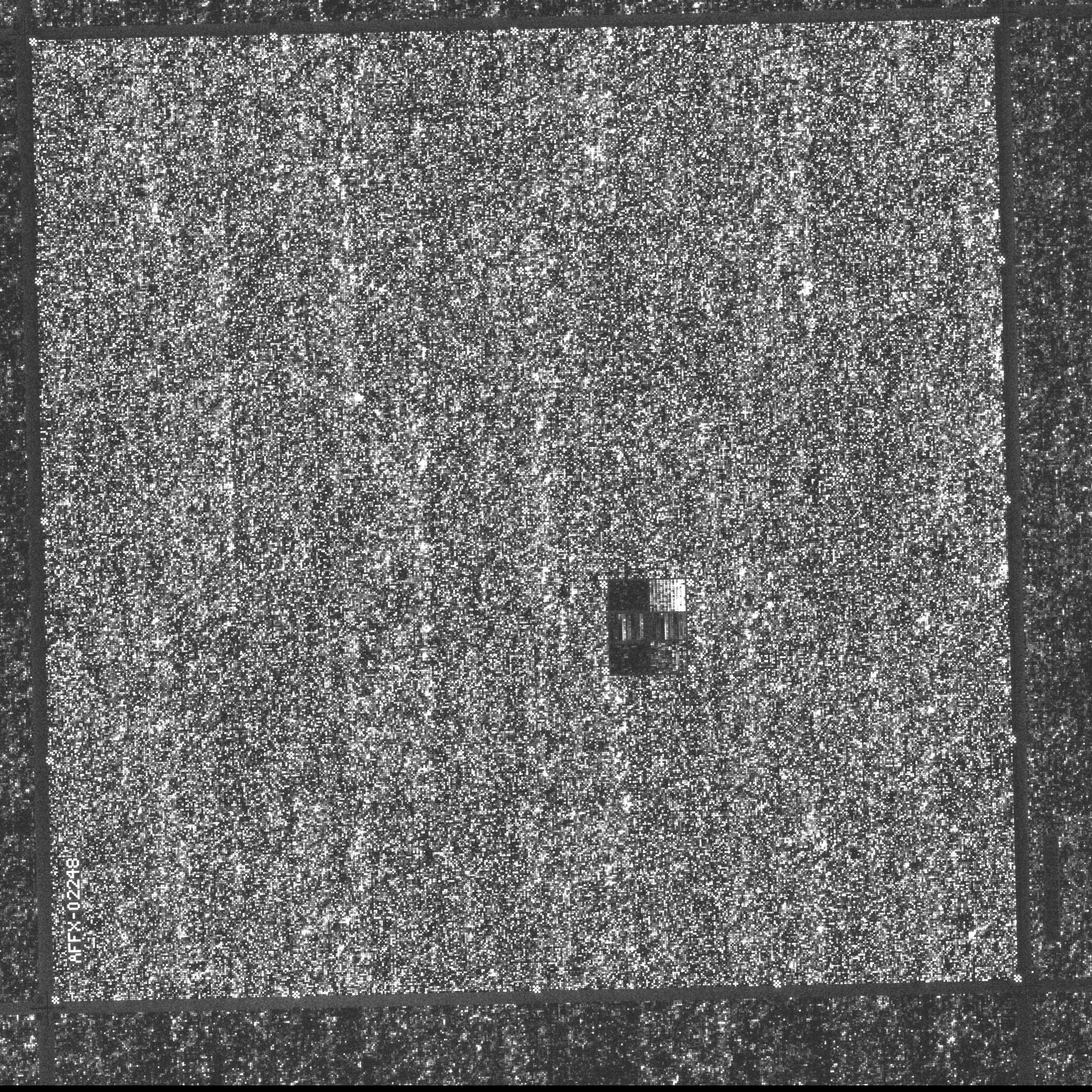

Supplement: Supplementary file 1 [file ijms-23-02615-s001.zip › Supplementary File 2/206/JPG files/P206_04_DPSC_SP_D14.JPG]

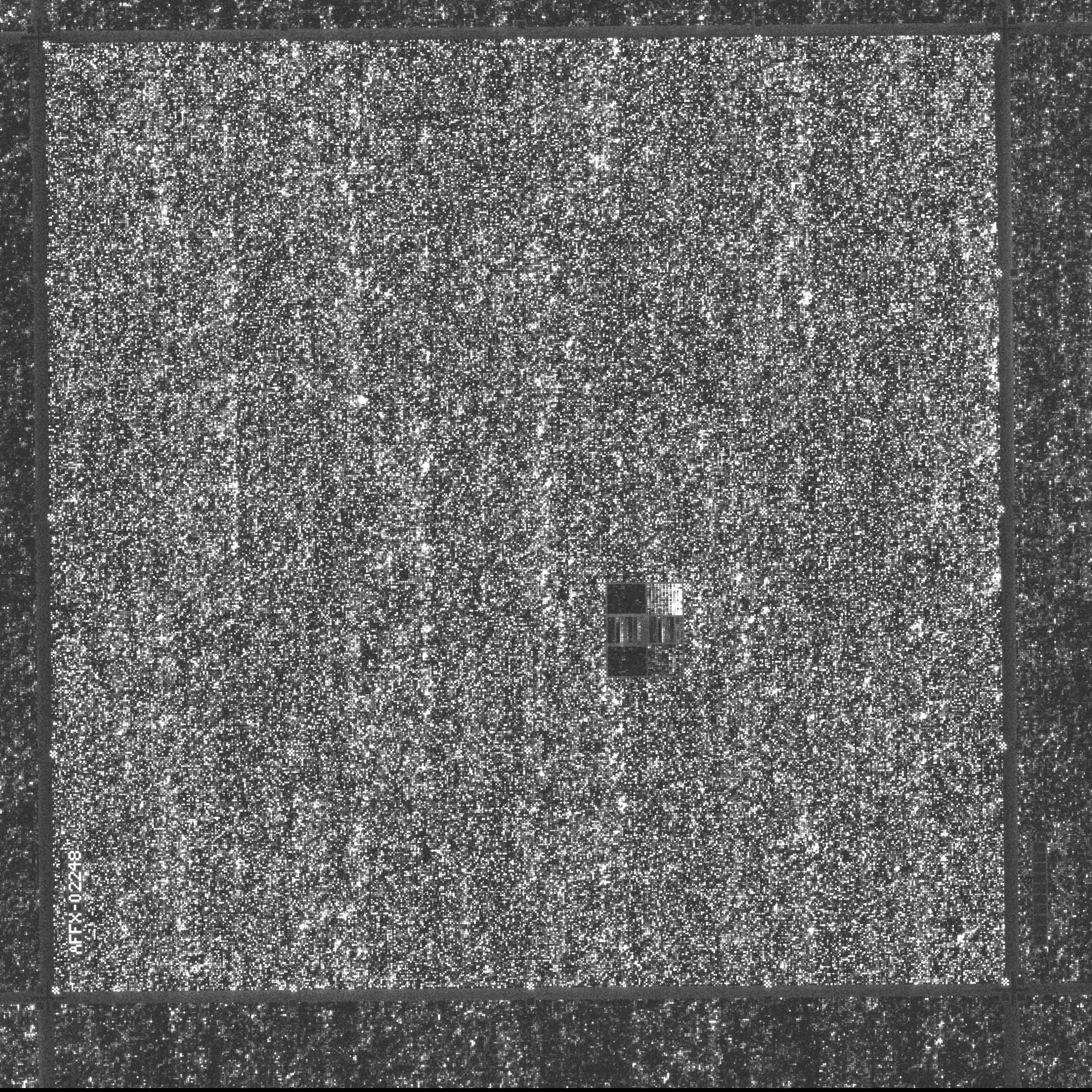

Supplement: Supplementary file 1 [file ijms-23-02615-s001.zip › Supplementary File 2/206/JPG files/P206_05_SCAP_10-FBS_D1.JPG]

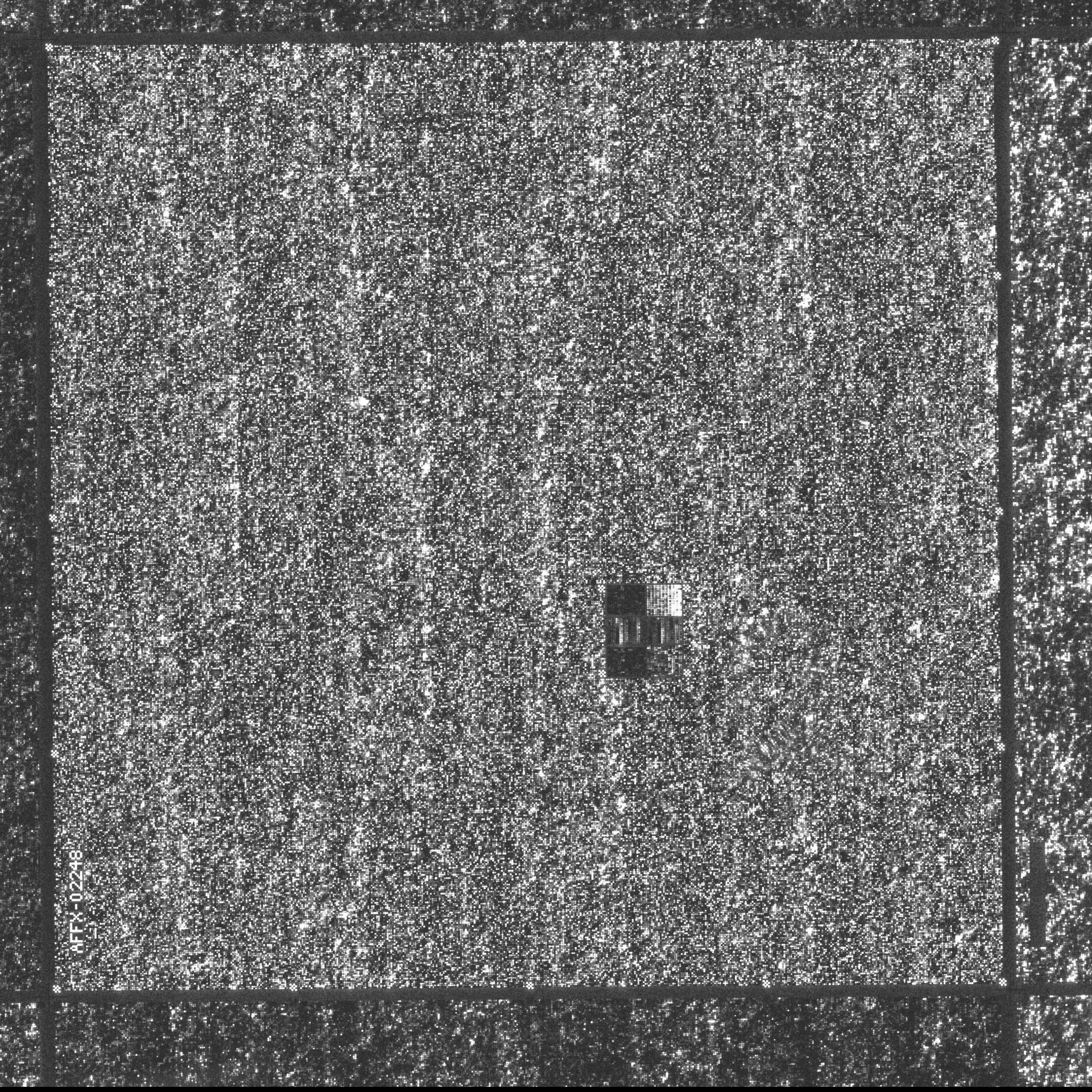

Supplement: Supplementary file 1 [file ijms-23-02615-s001.zip › Supplementary File 2/206/JPG files/P206_06_SCAP_10-FBS_D14.JPG]

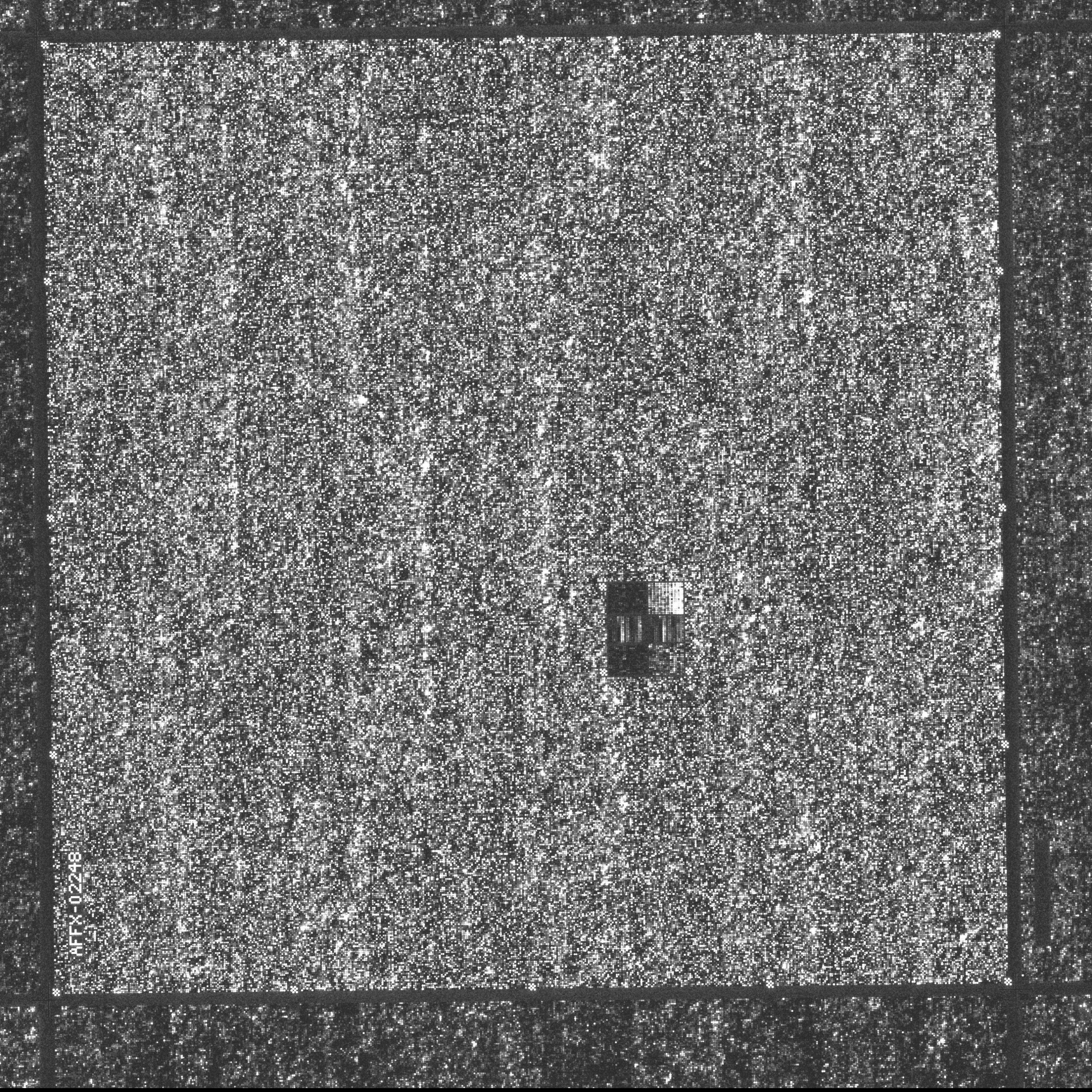

Supplement: Supplementary file 1 [file ijms-23-02615-s001.zip › Supplementary File 2/206/JPG files/P206_07_SCAP_SP_D1.JPG]

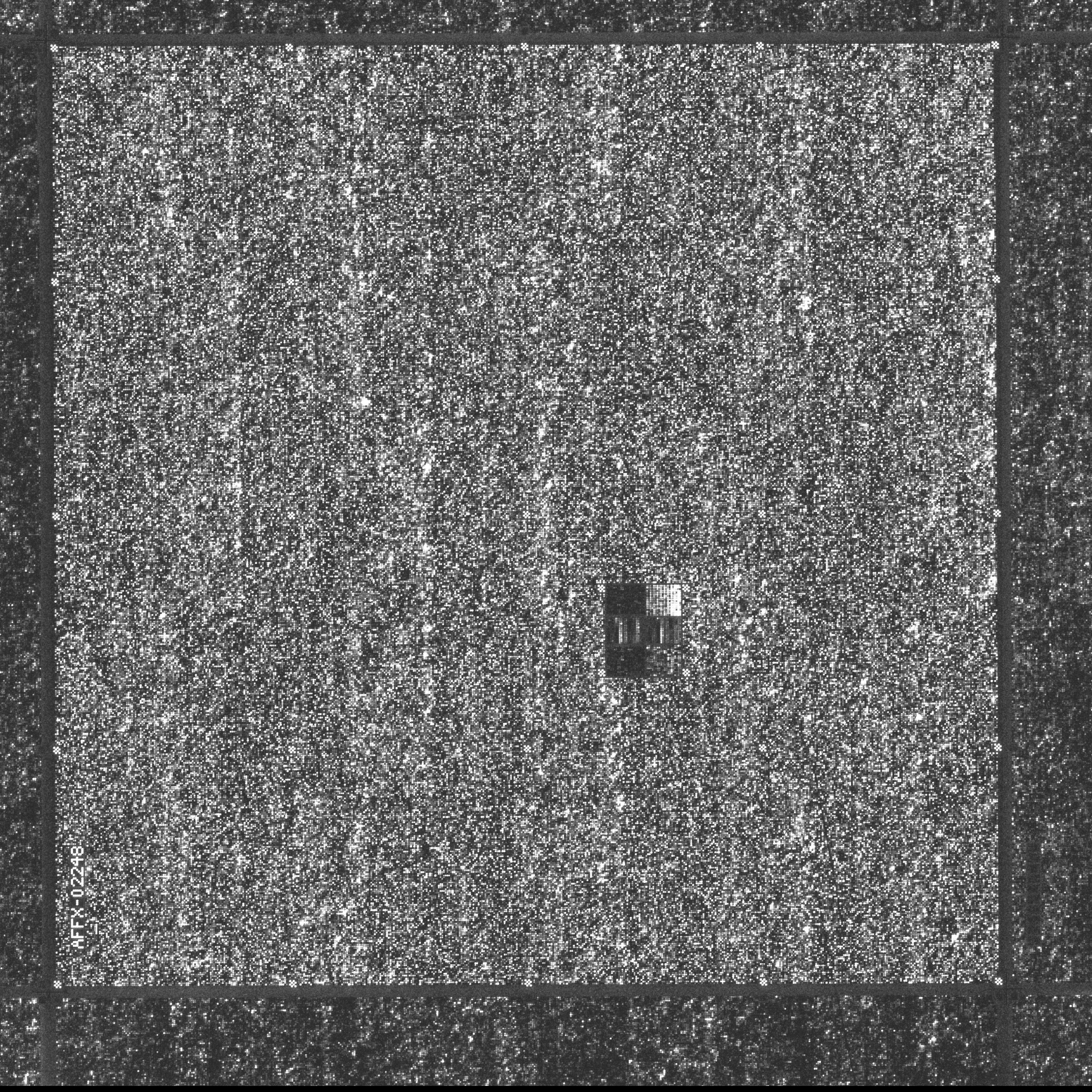

Supplement: Supplementary file 1 [file ijms-23-02615-s001.zip › Supplementary File 2/206/JPG files/P206_08_SCAP_SP_D14.JPG]

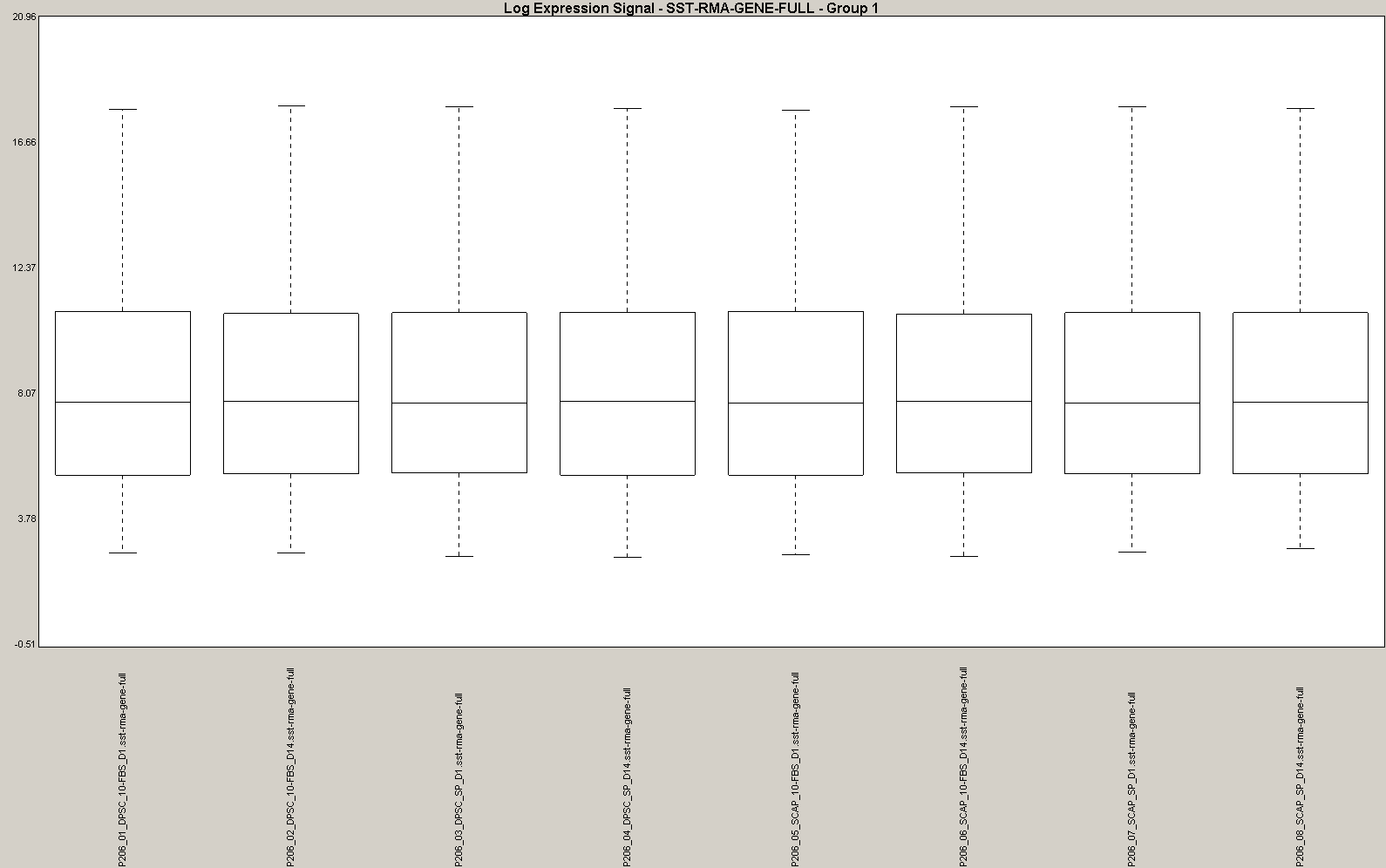

Supplement: Supplementary file 1 [file ijms-23-02615-s001.zip › Supplementary File 2/206/QC/P206_Log Expression Signal.PNG]

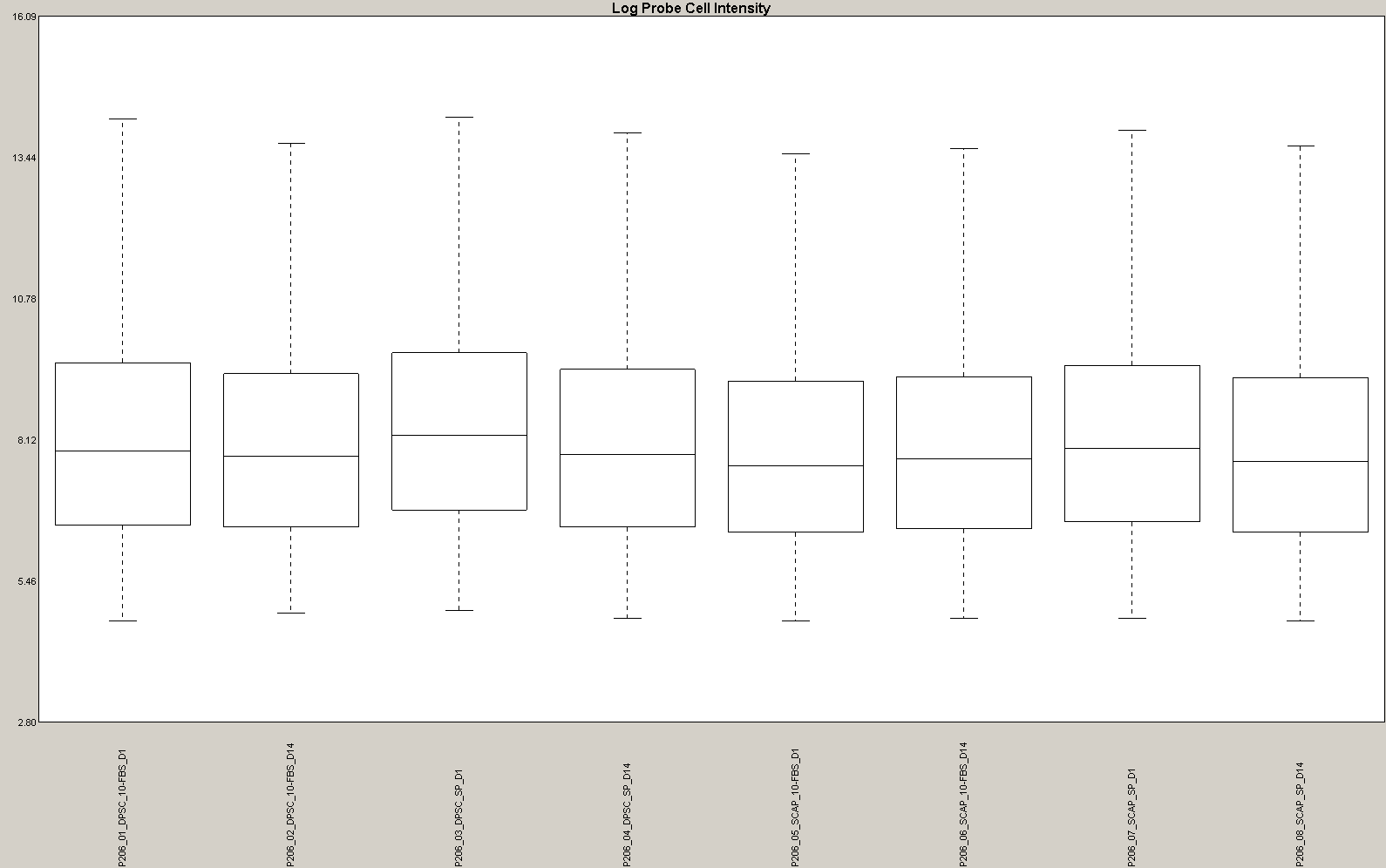

Supplement: Supplementary file 1 [file ijms-23-02615-s001.zip › Supplementary File 2/206/QC/P206_Log Probe Cell Intensity.PNG]

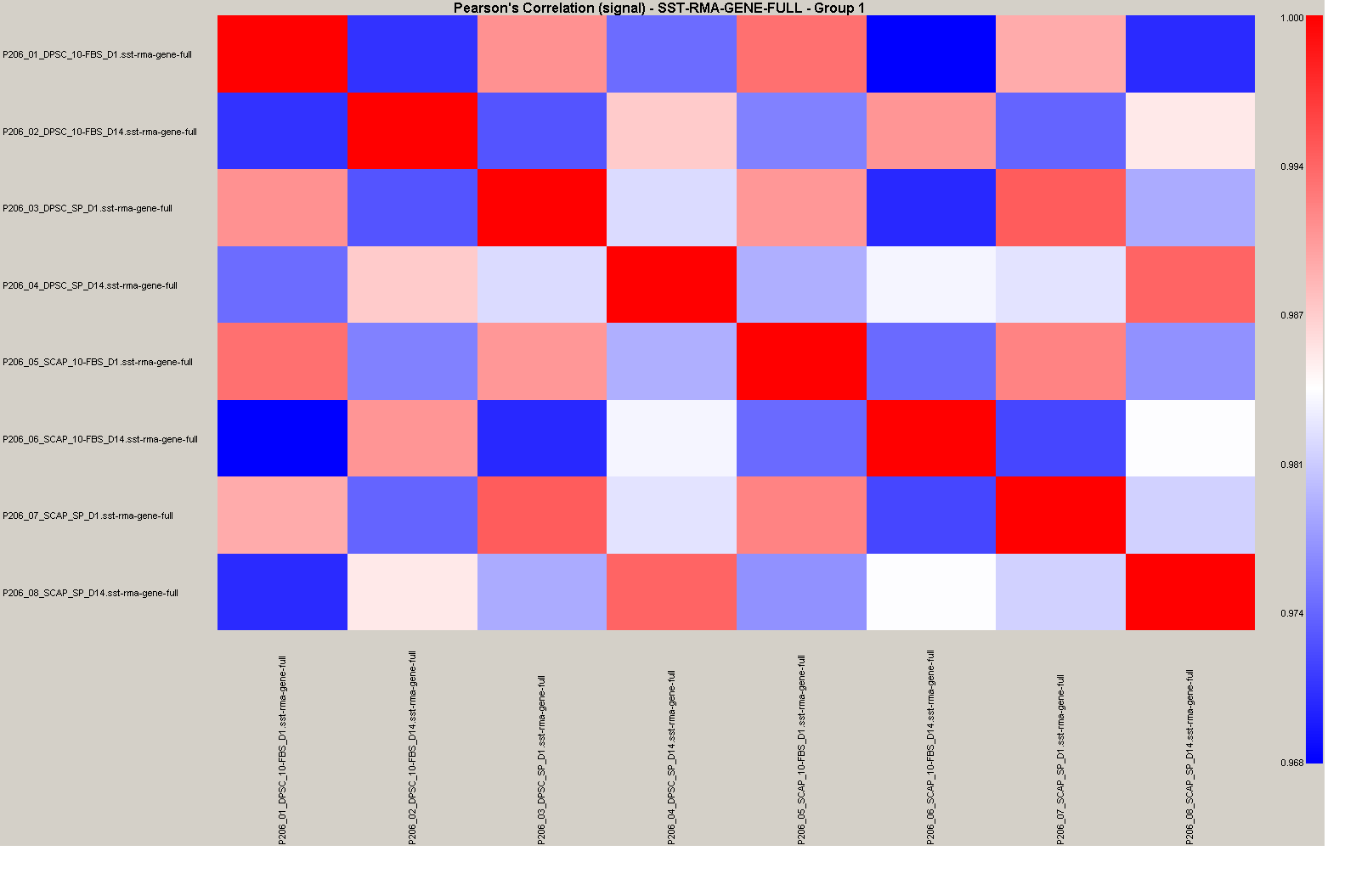

Supplement: Supplementary file 1 [file ijms-23-02615-s001.zip › Supplementary File 2/206/QC/P206_Pearsons Correlation.PNG]

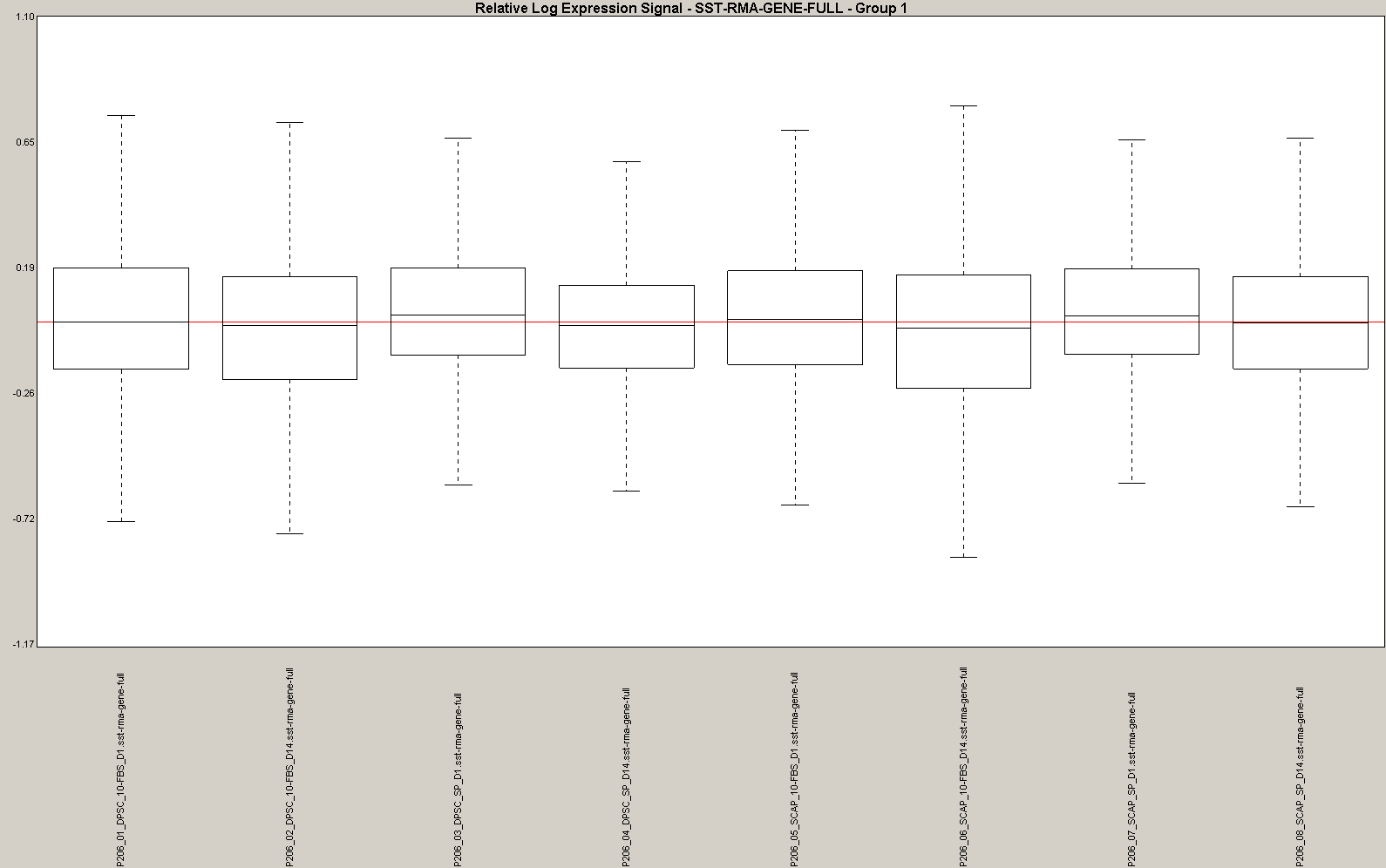

Supplement: Supplementary file 1 [file ijms-23-02615-s001.zip › Supplementary File 2/206/QC/P206_Relative Log Expression Signal.PNG]

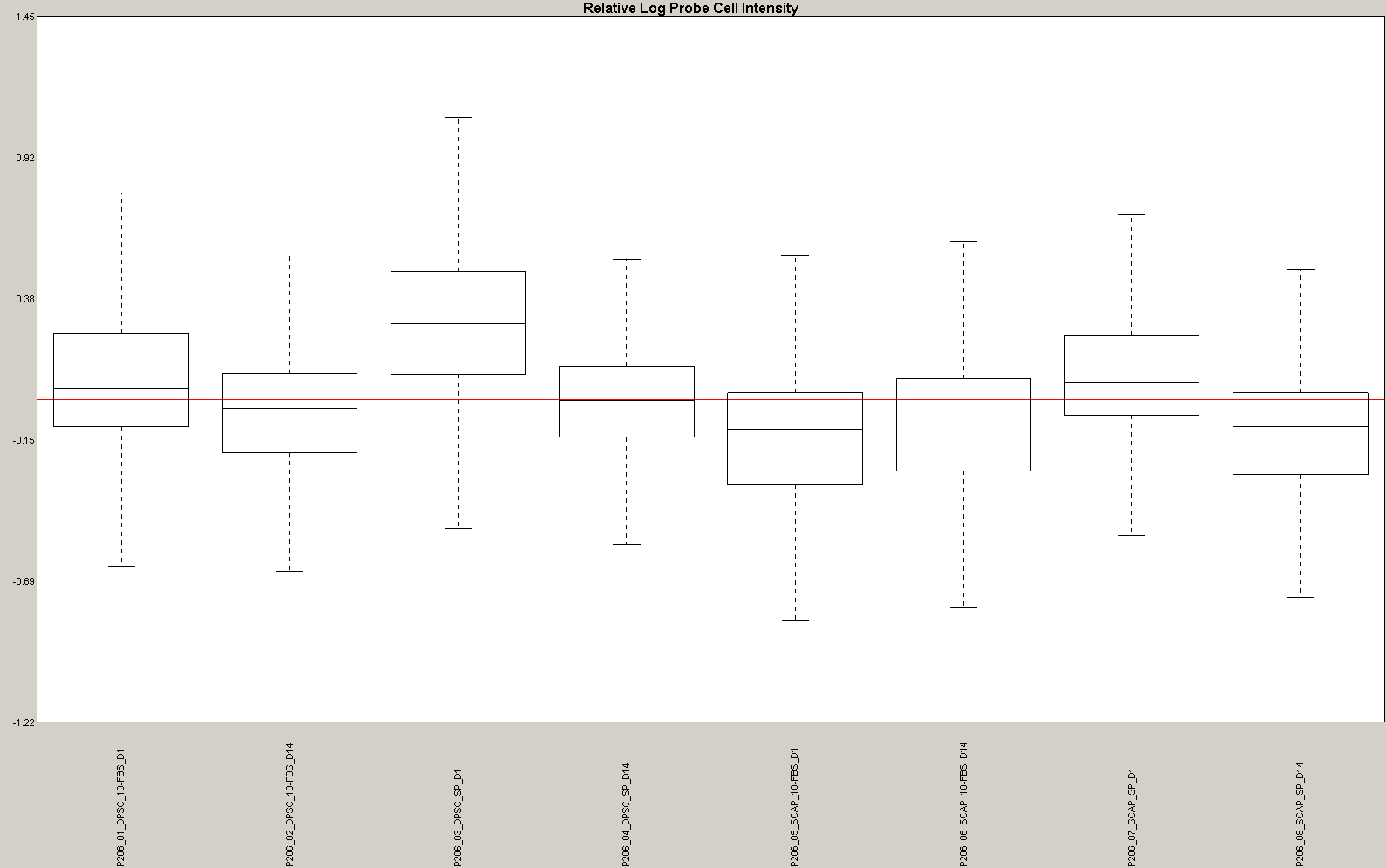

Supplement: Supplementary file 1 [file ijms-23-02615-s001.zip › Supplementary File 2/206/QC/P206_Relative Log Probe Cell Intensity.PNG]

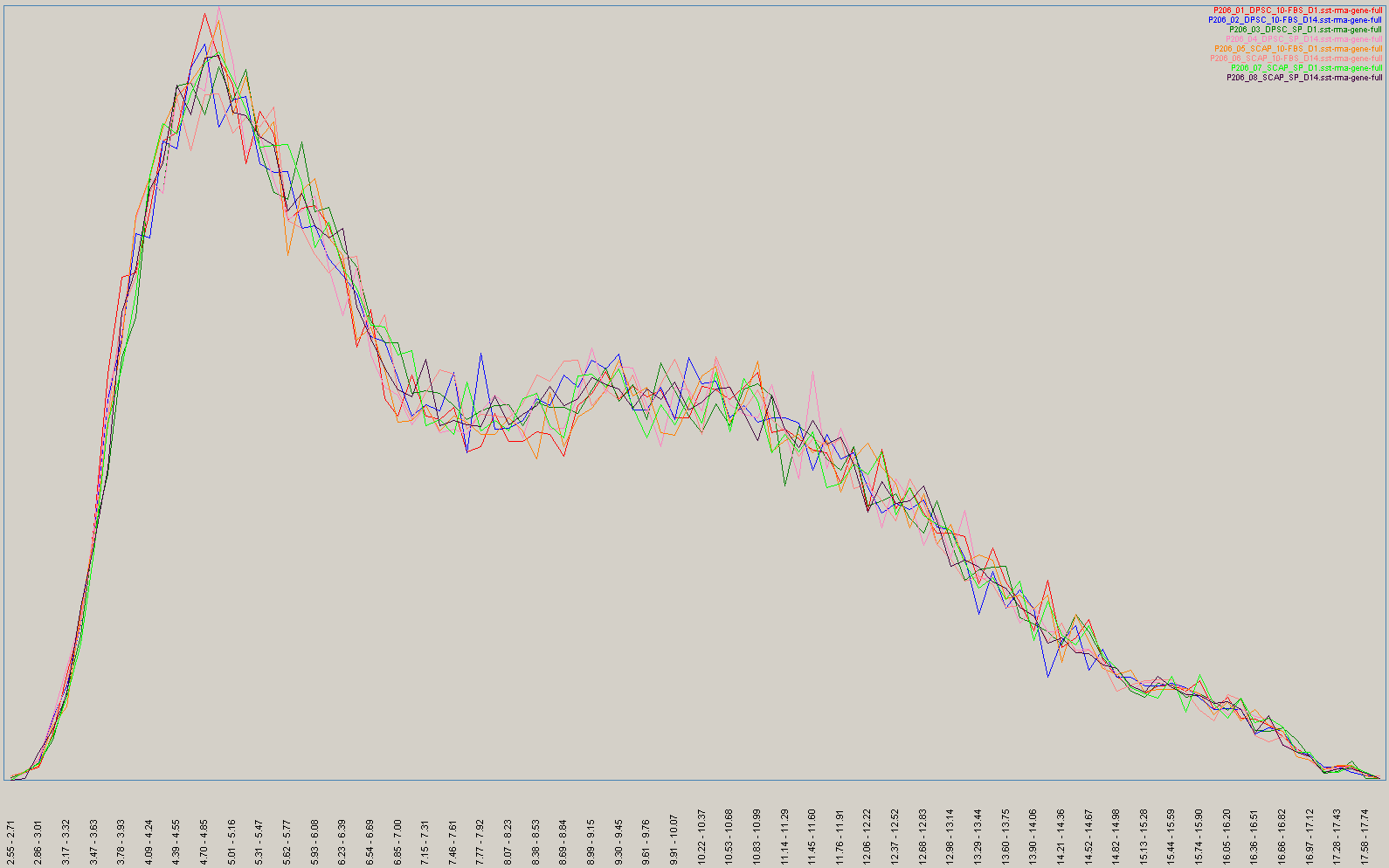

Supplement: Supplementary file 1 [file ijms-23-02615-s001.zip › Supplementary File 2/206/QC/P206_Signal Histogram.PNG]

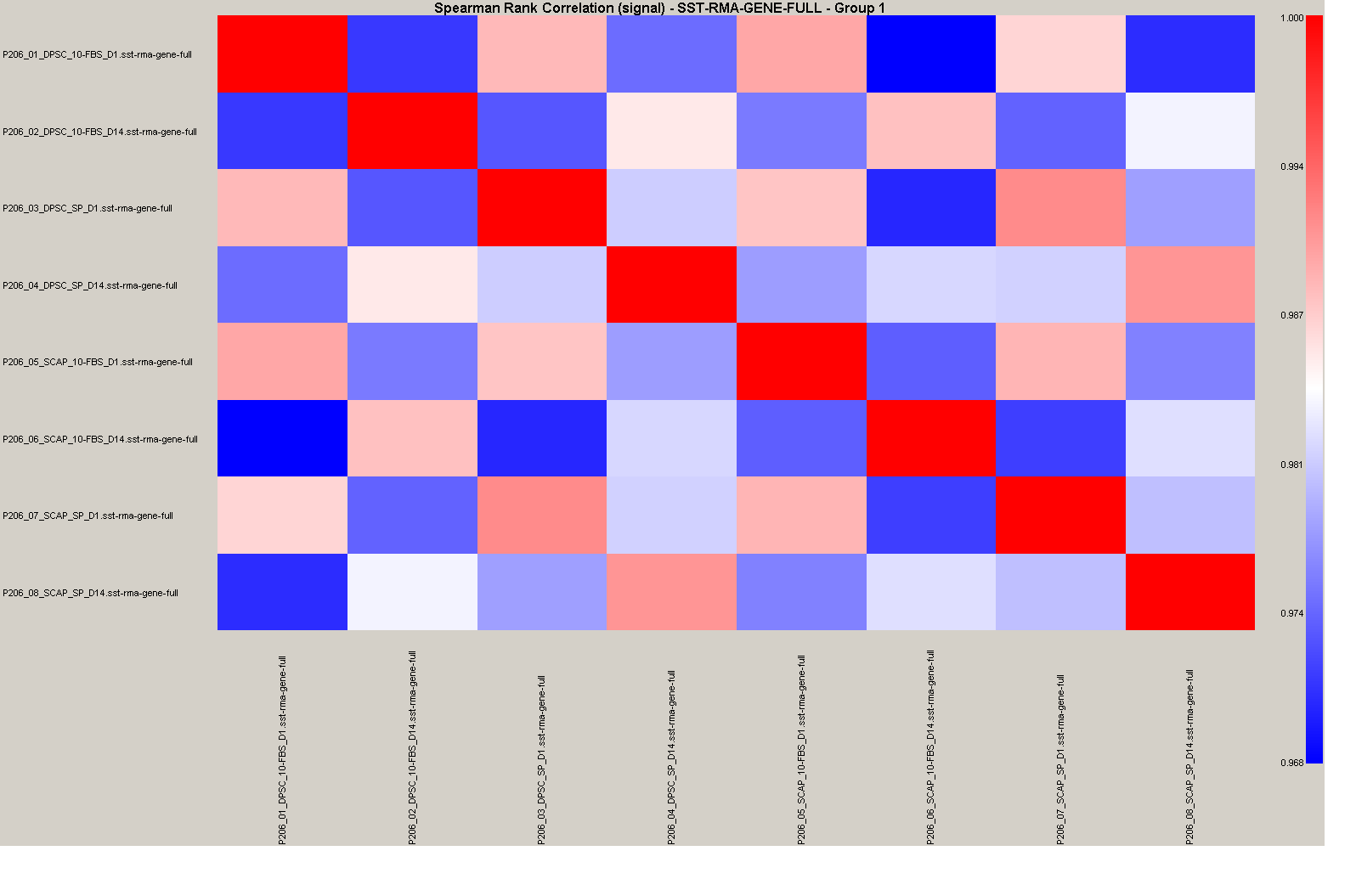

Supplement: Supplementary file 1 [file ijms-23-02615-s001.zip › Supplementary File 2/206/QC/P206_Spearman Rank Correlation.PNG]

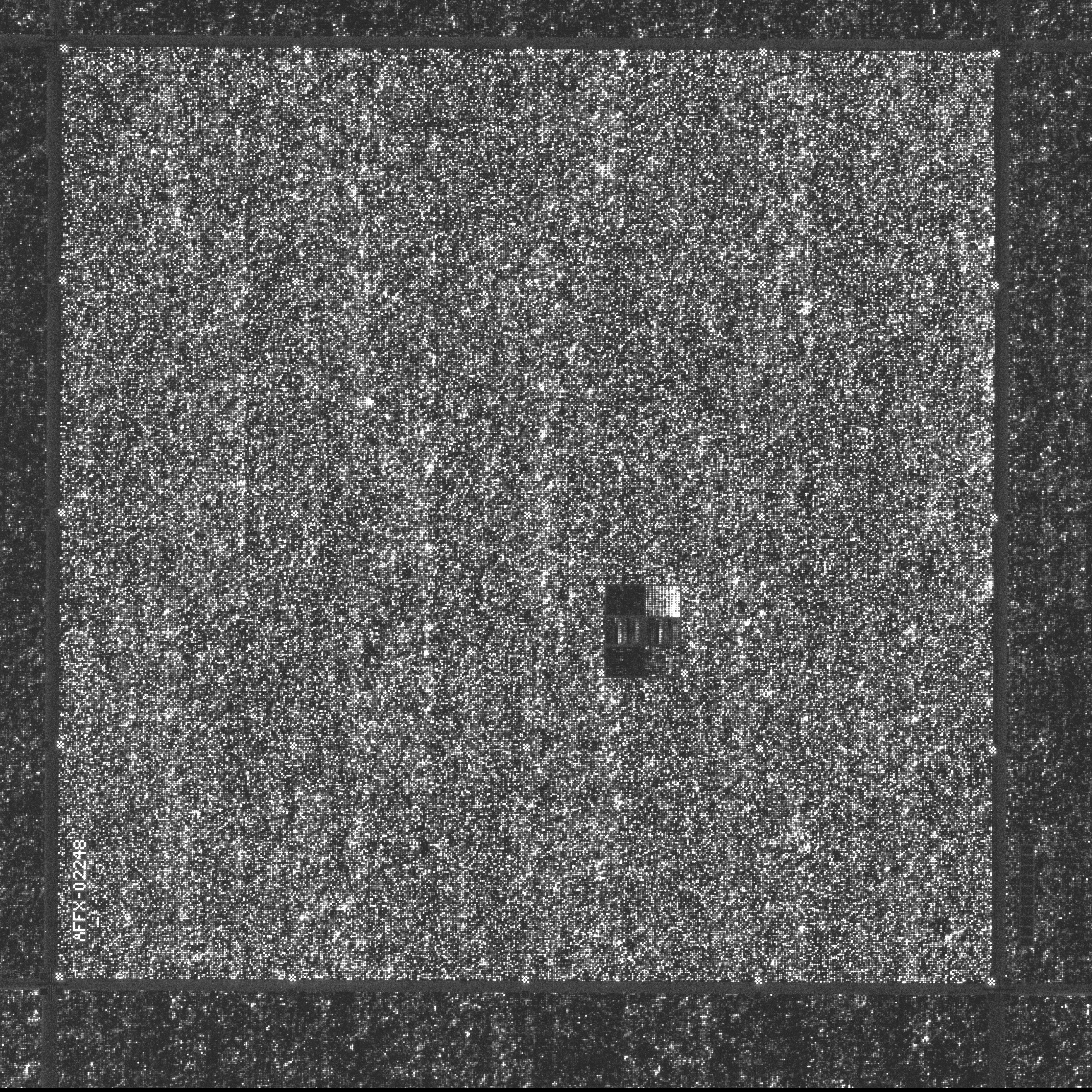

Supplement: Supplementary file 1 [file ijms-23-02615-s001.zip › Supplementary File 2/229/JPG files/P229_01_DPSC_10-FBS_D1_wdh.JPG]

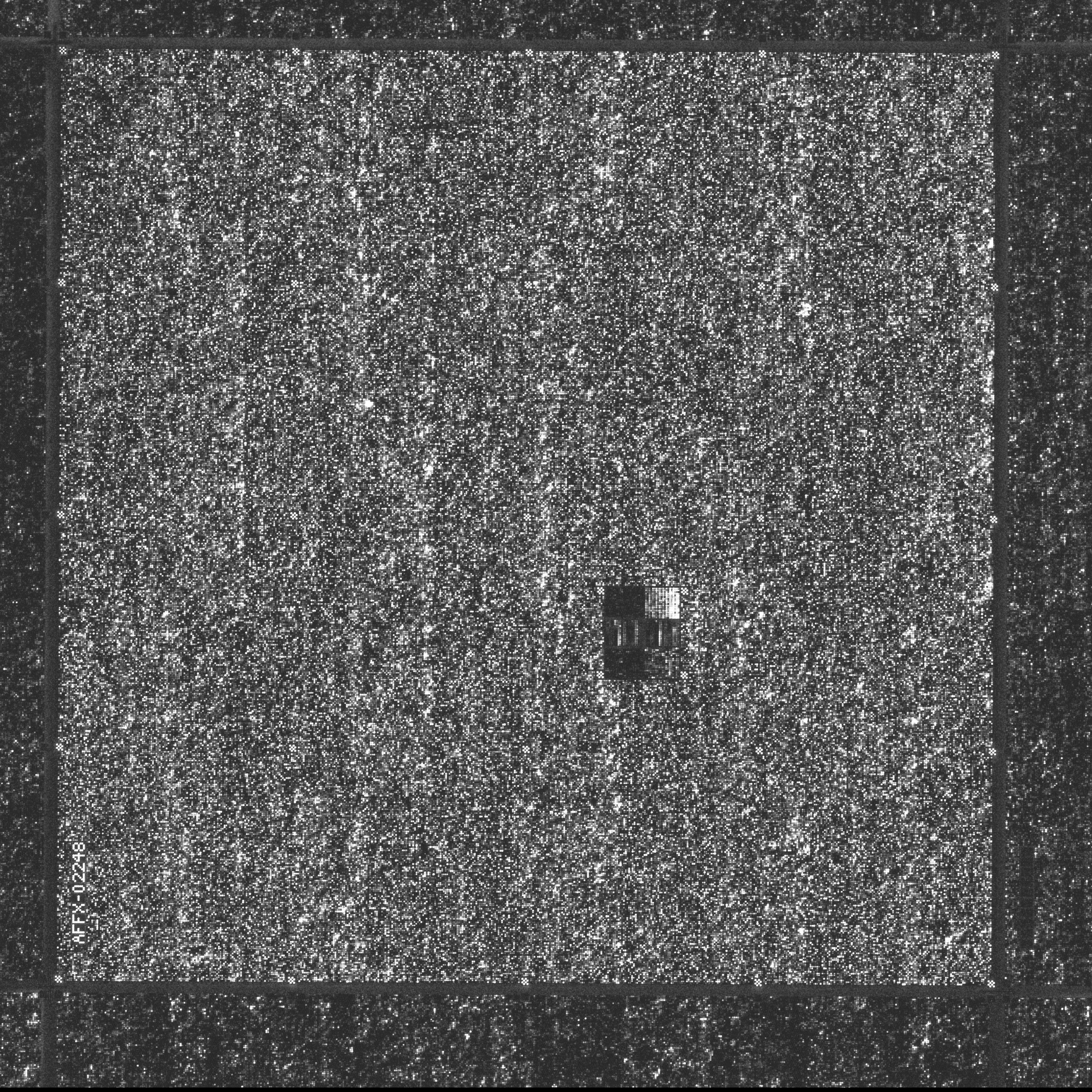

Supplement: Supplementary file 1 [file ijms-23-02615-s001.zip › Supplementary File 2/229/JPG files/P229_02_DPSC_10-FBS_D14_wdh.JPG]

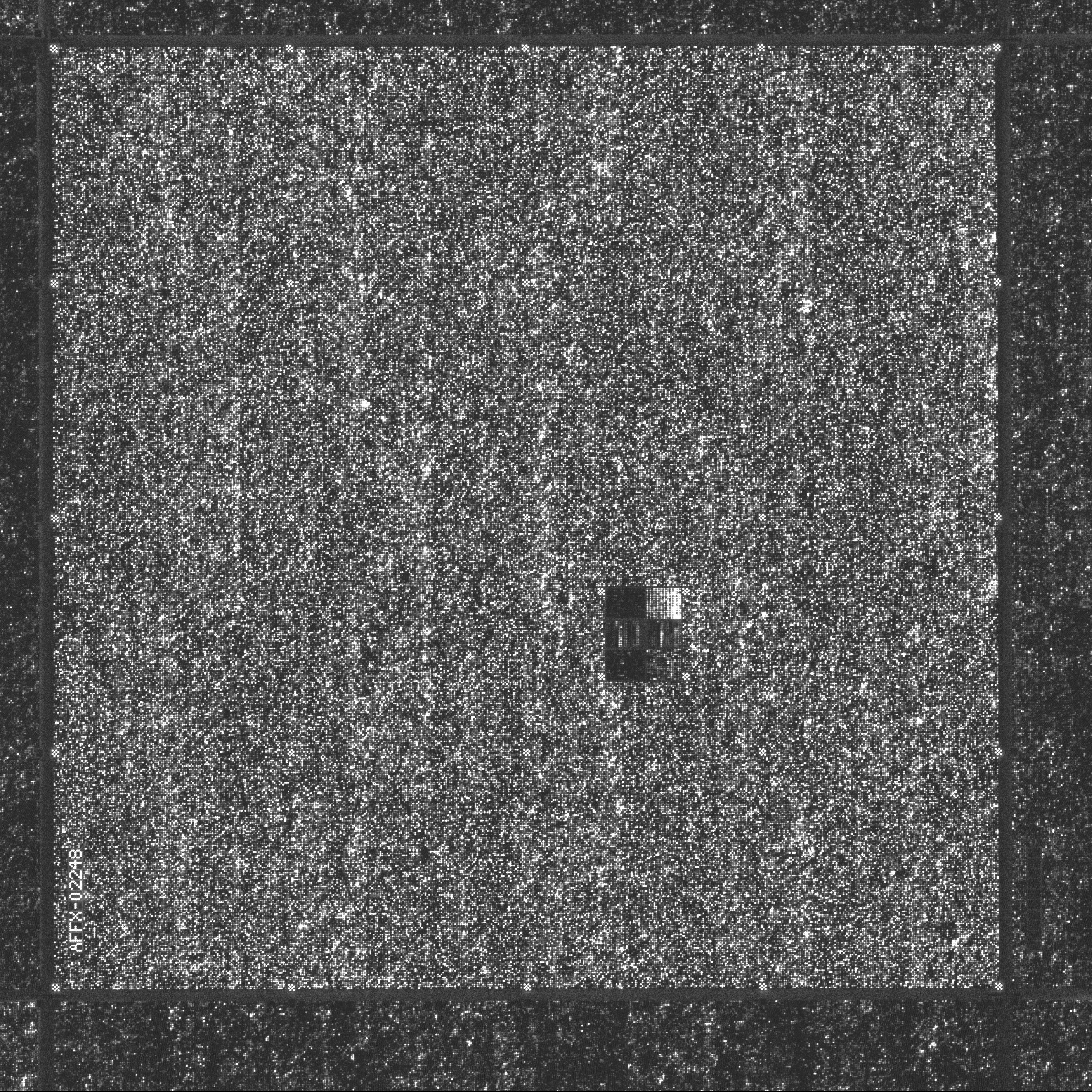

Supplement: Supplementary file 1 [file ijms-23-02615-s001.zip › Supplementary File 2/229/JPG files/P229_03_DPSC_SP_D1_wdh.JPG]

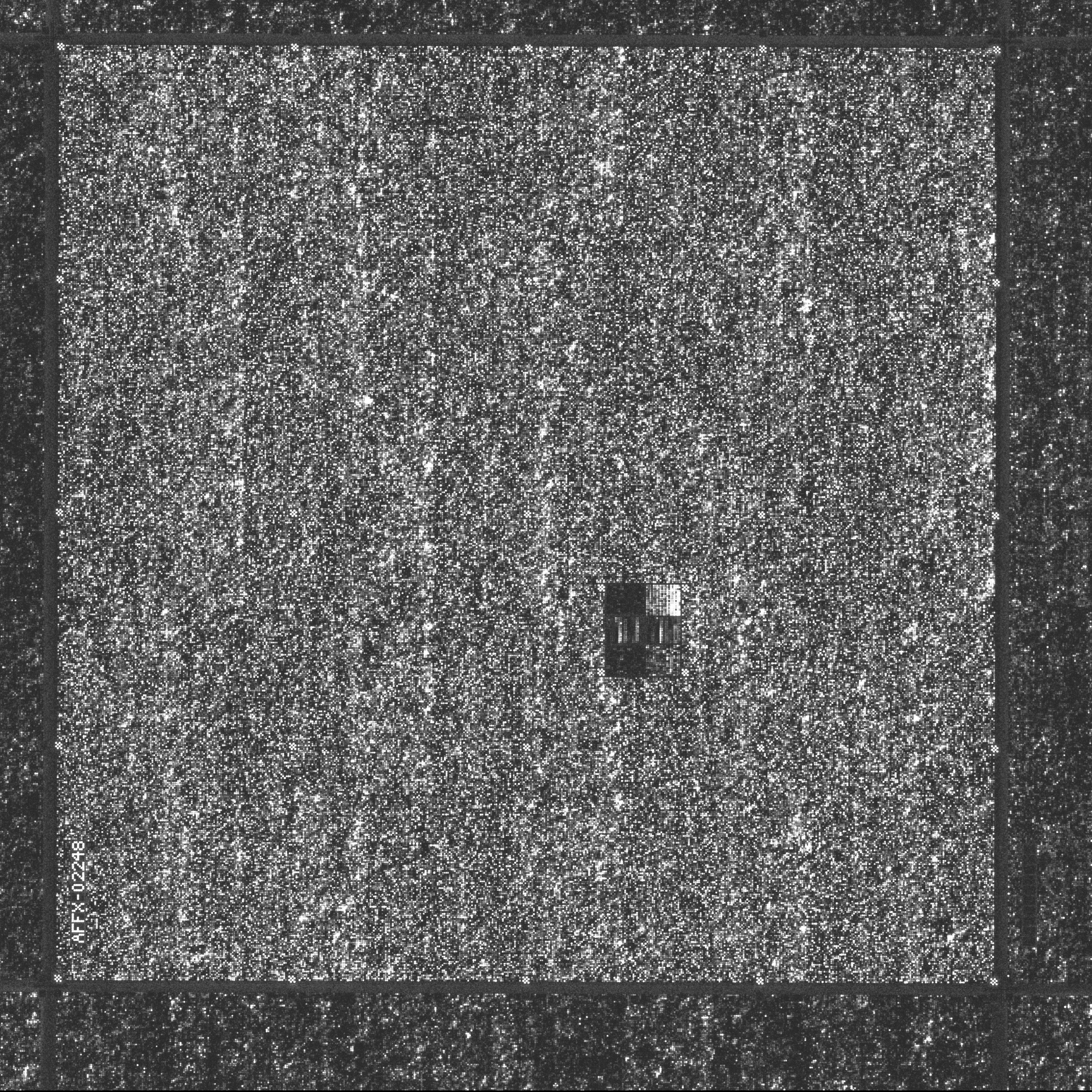

Supplement: Supplementary file 1 [file ijms-23-02615-s001.zip › Supplementary File 2/229/JPG files/P229_04_DPSC_SP_D14_wdh.JPG]

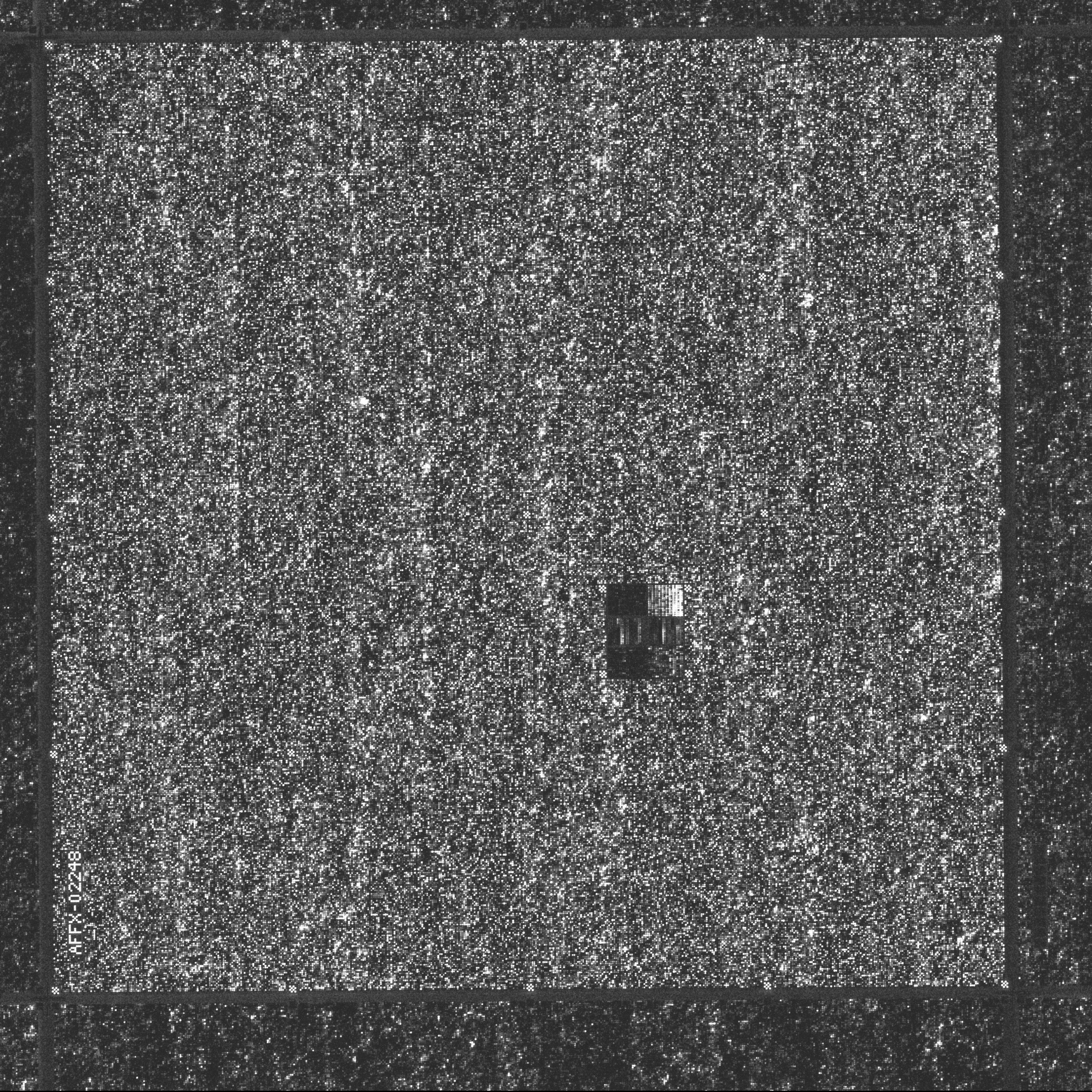

Supplement: Supplementary file 1 [file ijms-23-02615-s001.zip › Supplementary File 2/229/JPG files/P229_05_SCAP_10-FBS_D1_wdh.JPG]

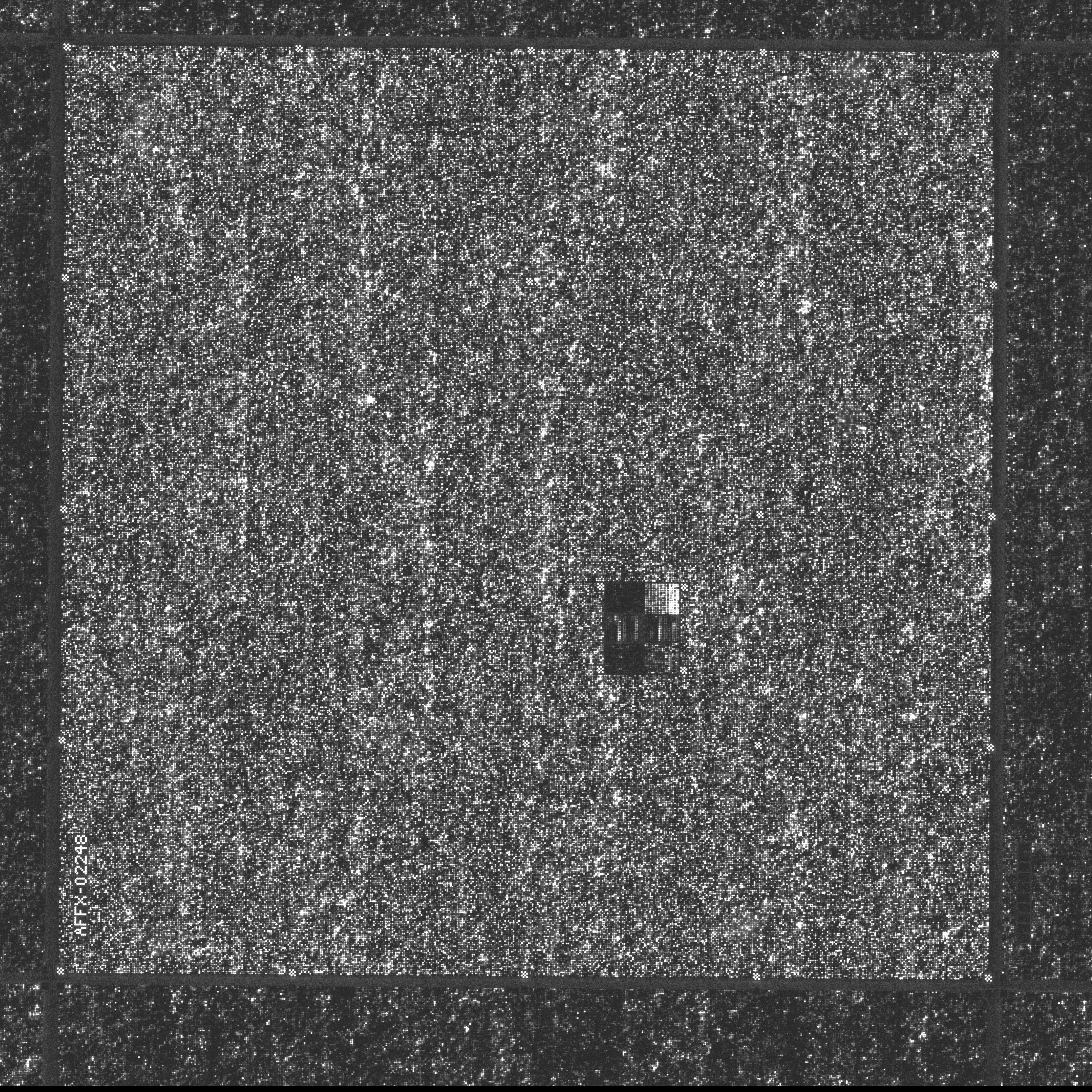

Supplement: Supplementary file 1 [file ijms-23-02615-s001.zip › Supplementary File 2/229/JPG files/P229_06_SCAP_10-FBS_D14_wdh.JPG]

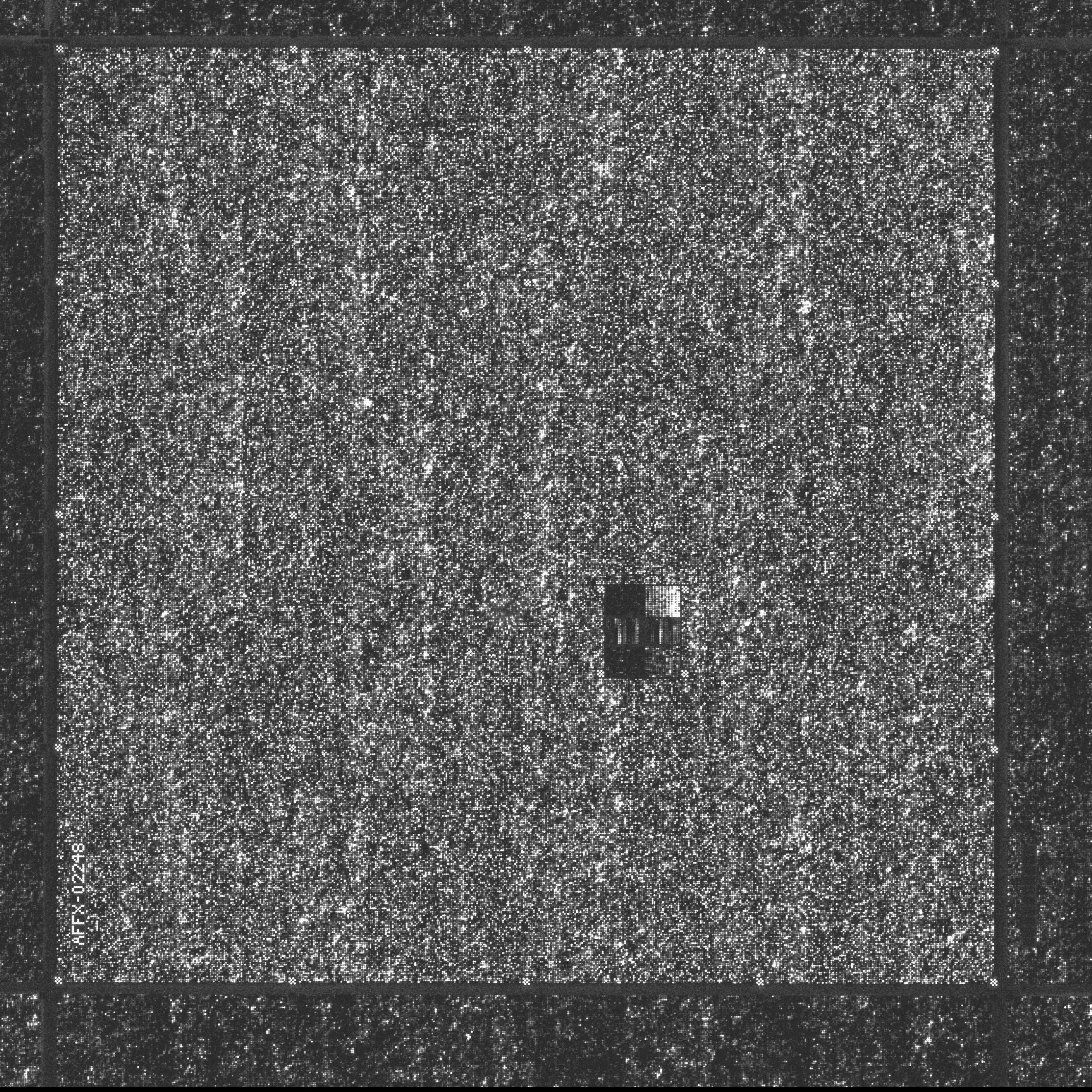

Supplement: Supplementary file 1 [file ijms-23-02615-s001.zip › Supplementary File 2/229/JPG files/P229_07_SCAP_SP_D1_wdh.JPG]

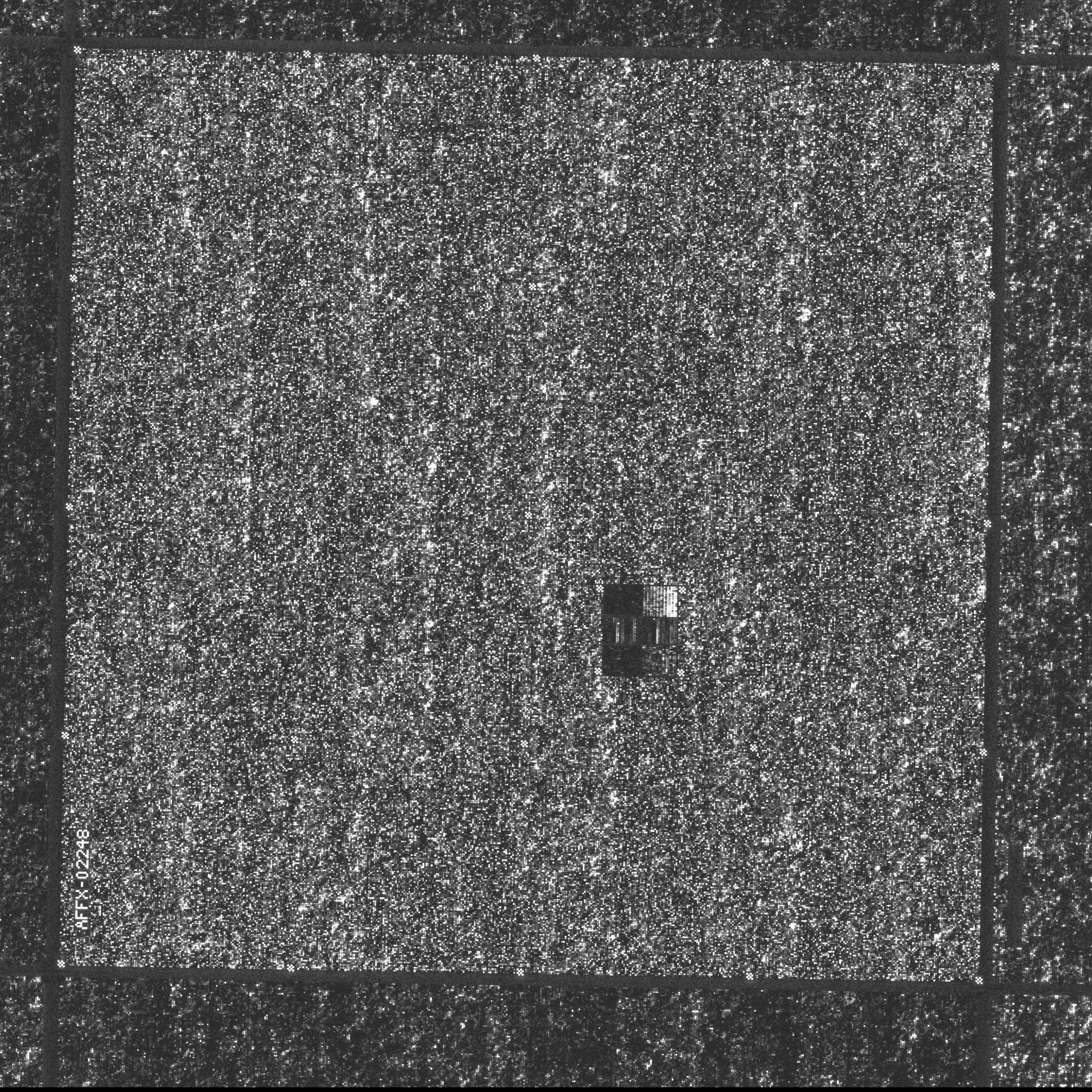

Supplement: Supplementary file 1 [file ijms-23-02615-s001.zip › Supplementary File 2/229/JPG files/P229_08_SCAP_SP_D14_wdh.JPG]

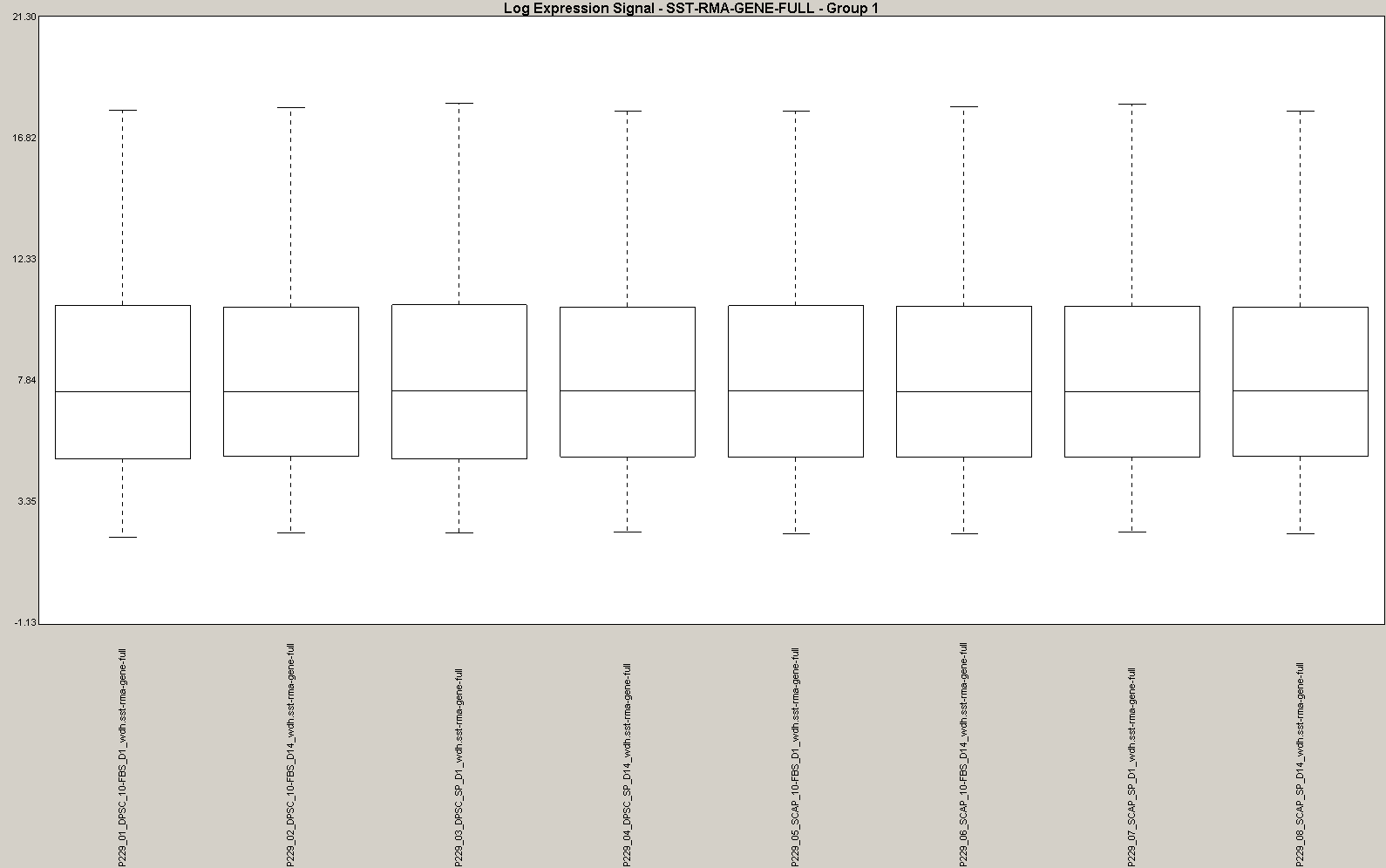

Supplement: Supplementary file 1 [file ijms-23-02615-s001.zip › Supplementary File 2/229/QC/P228_Log Expression Signal.PNG]

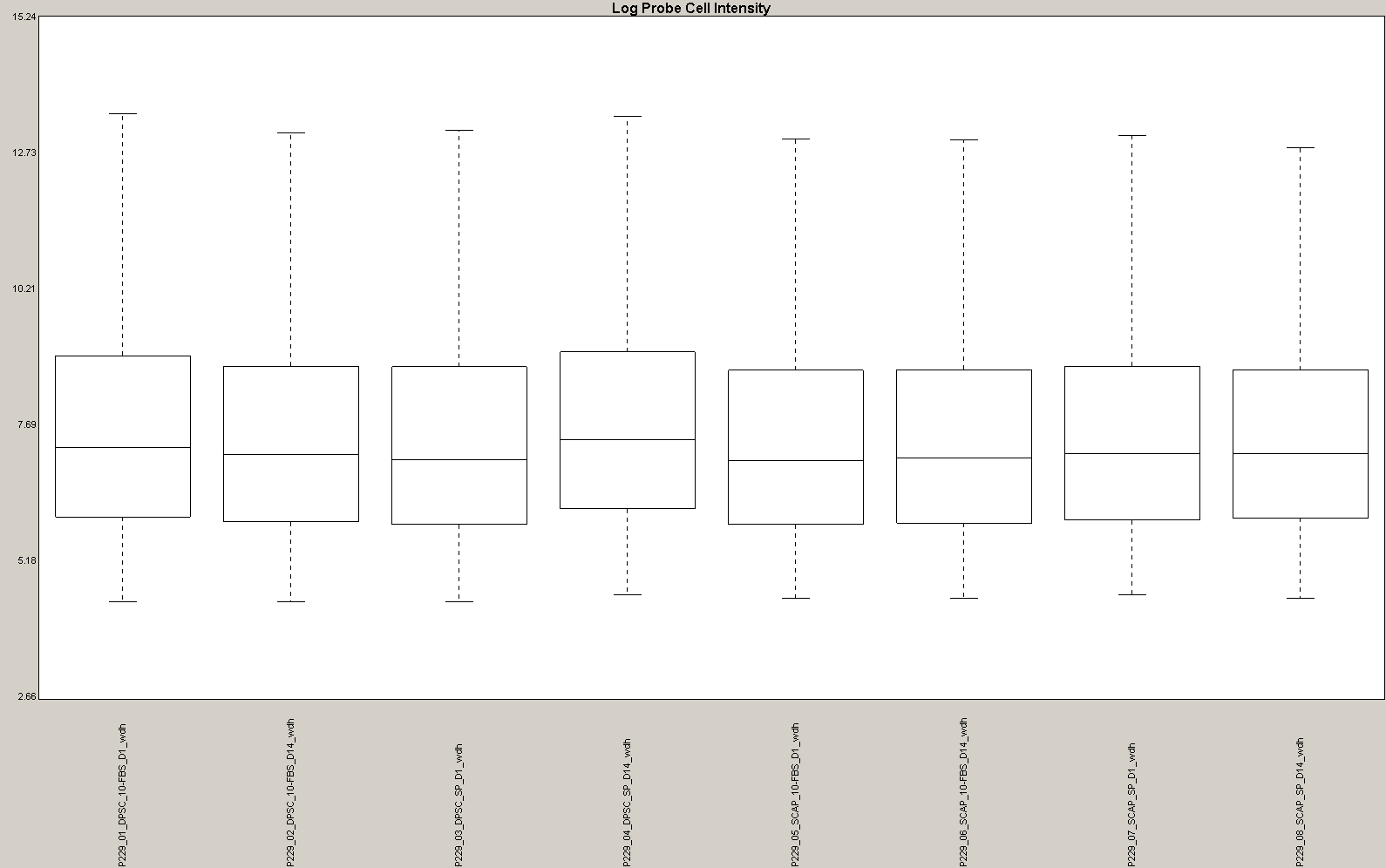

Supplement: Supplementary file 1 [file ijms-23-02615-s001.zip › Supplementary File 2/229/QC/P228_Log Probe Cell Intensity.PNG]

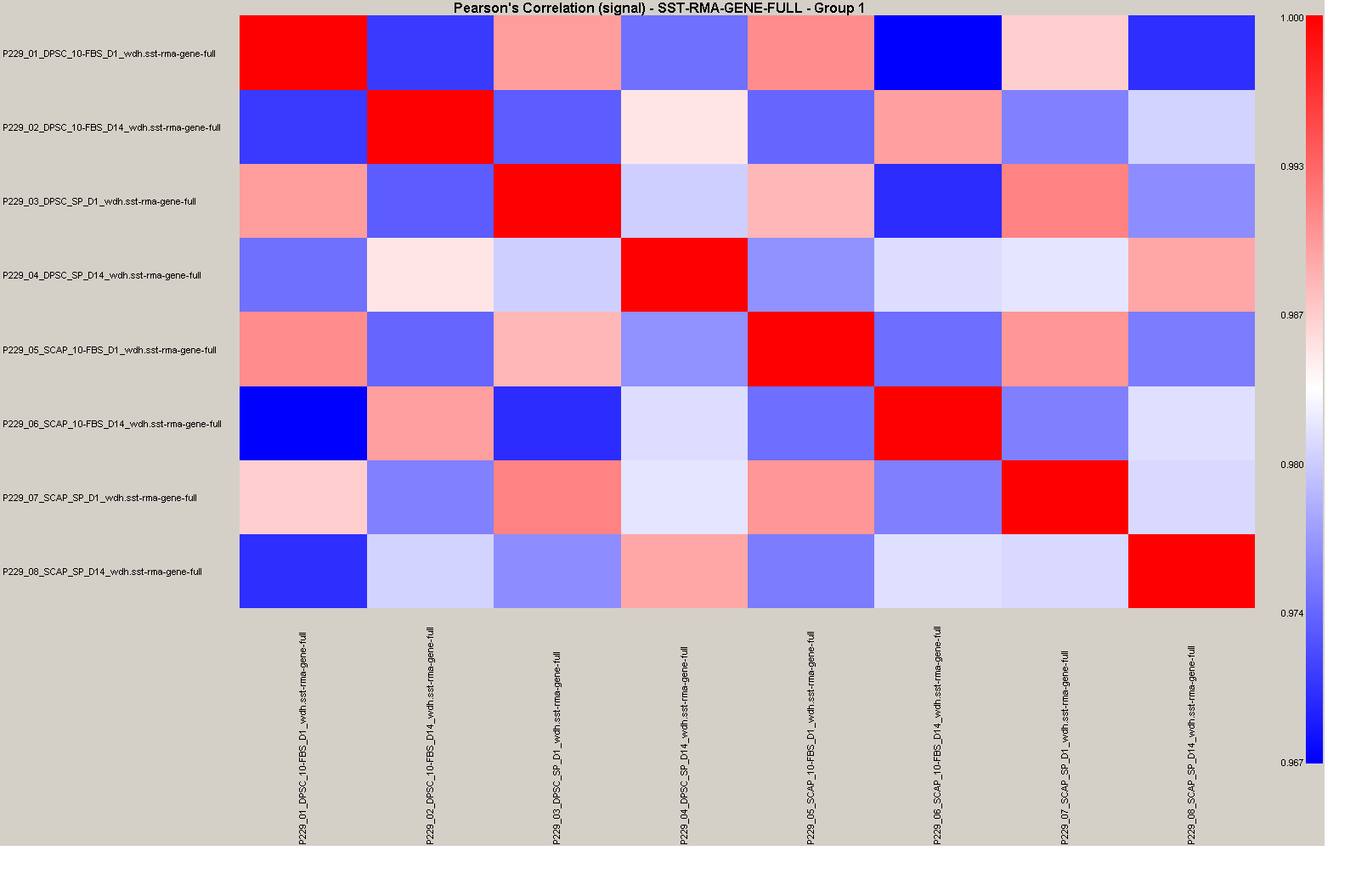

Supplement: Supplementary file 1 [file ijms-23-02615-s001.zip › Supplementary File 2/229/QC/P228_Pearsons Correlation.PNG]

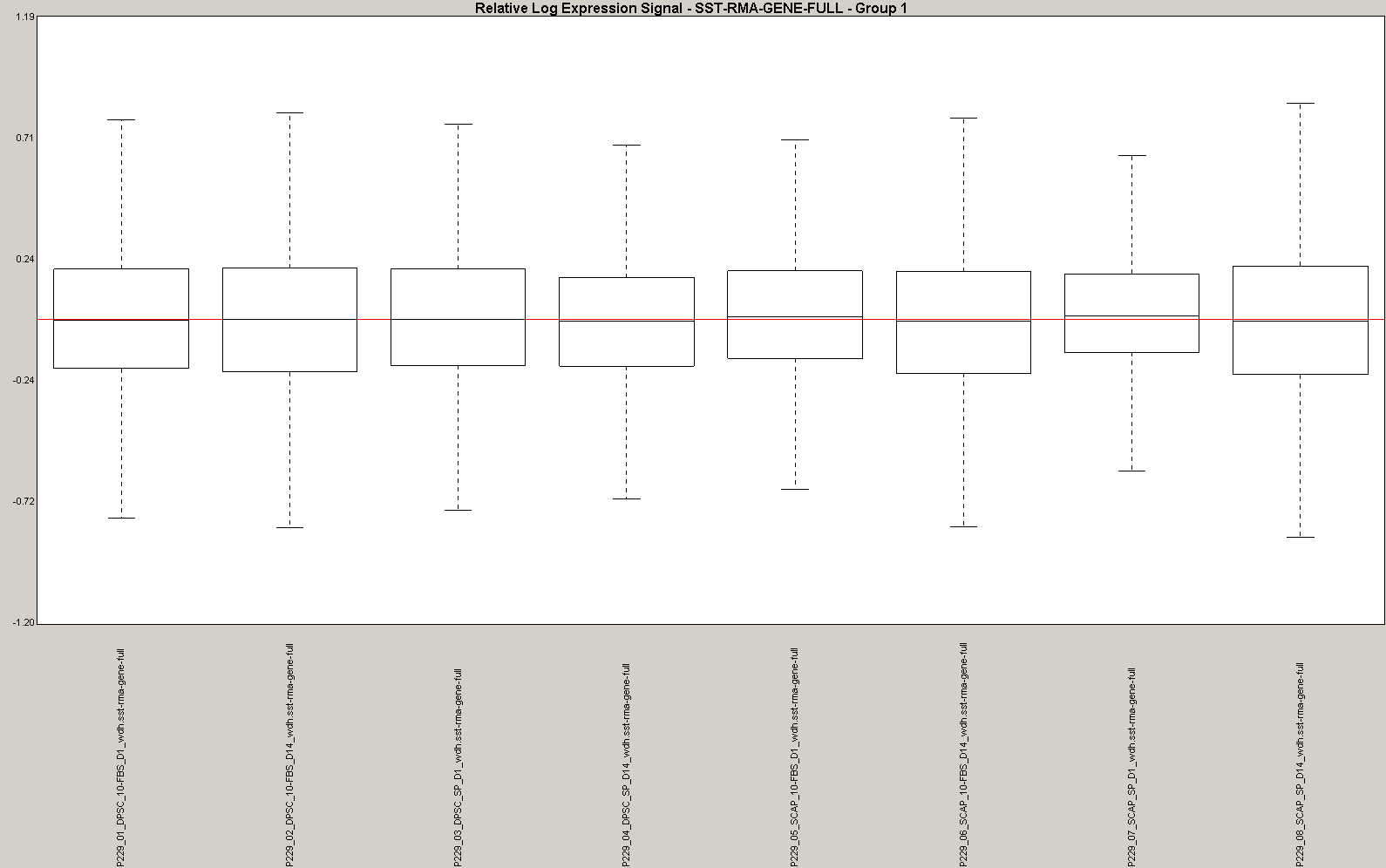

Supplement: Supplementary file 1 [file ijms-23-02615-s001.zip › Supplementary File 2/229/QC/P228_Relative Log Expression Signal.PNG]

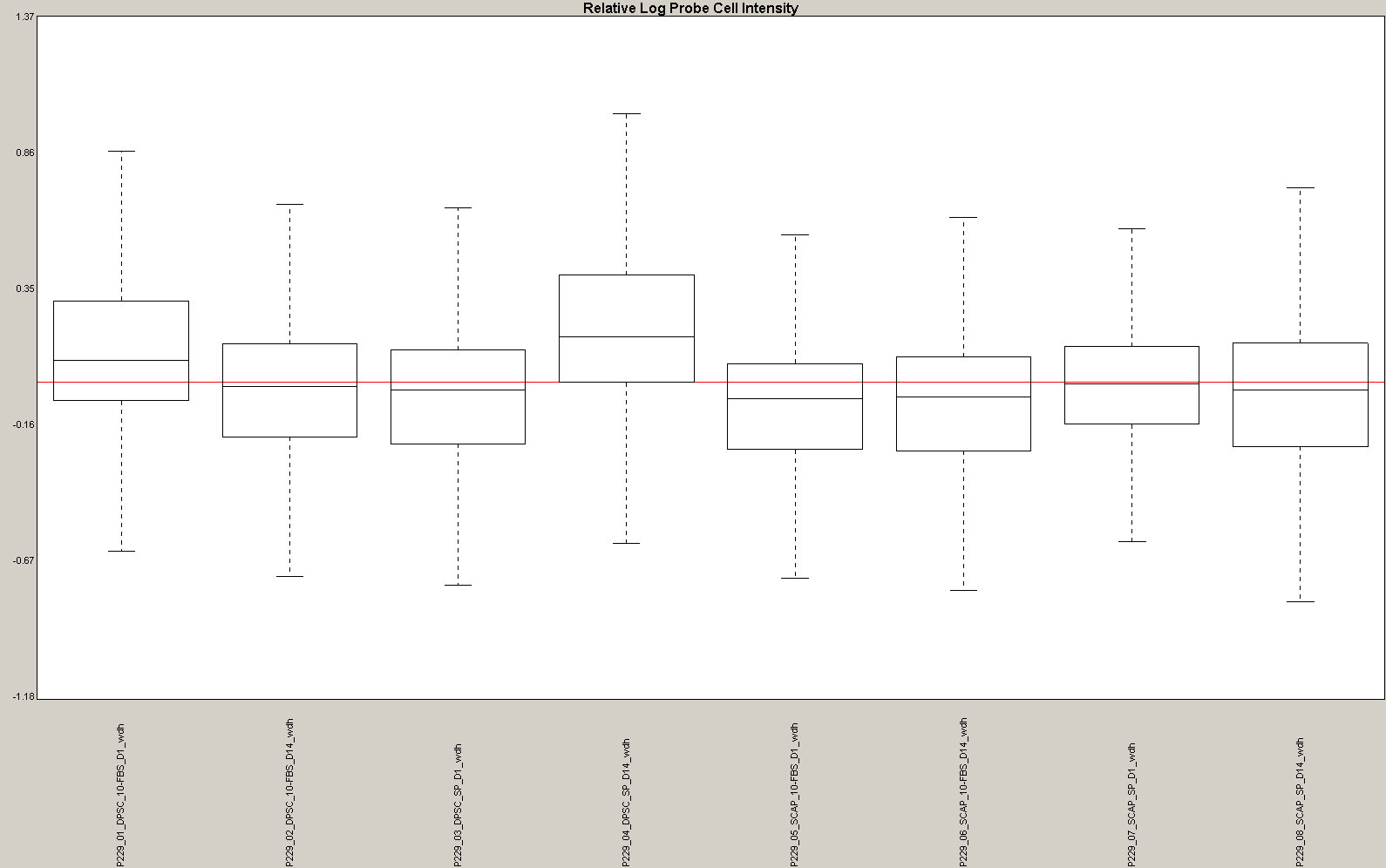

Supplement: Supplementary file 1 [file ijms-23-02615-s001.zip › Supplementary File 2/229/QC/P228_Relative Log Probe Cell Intensity.PNG]

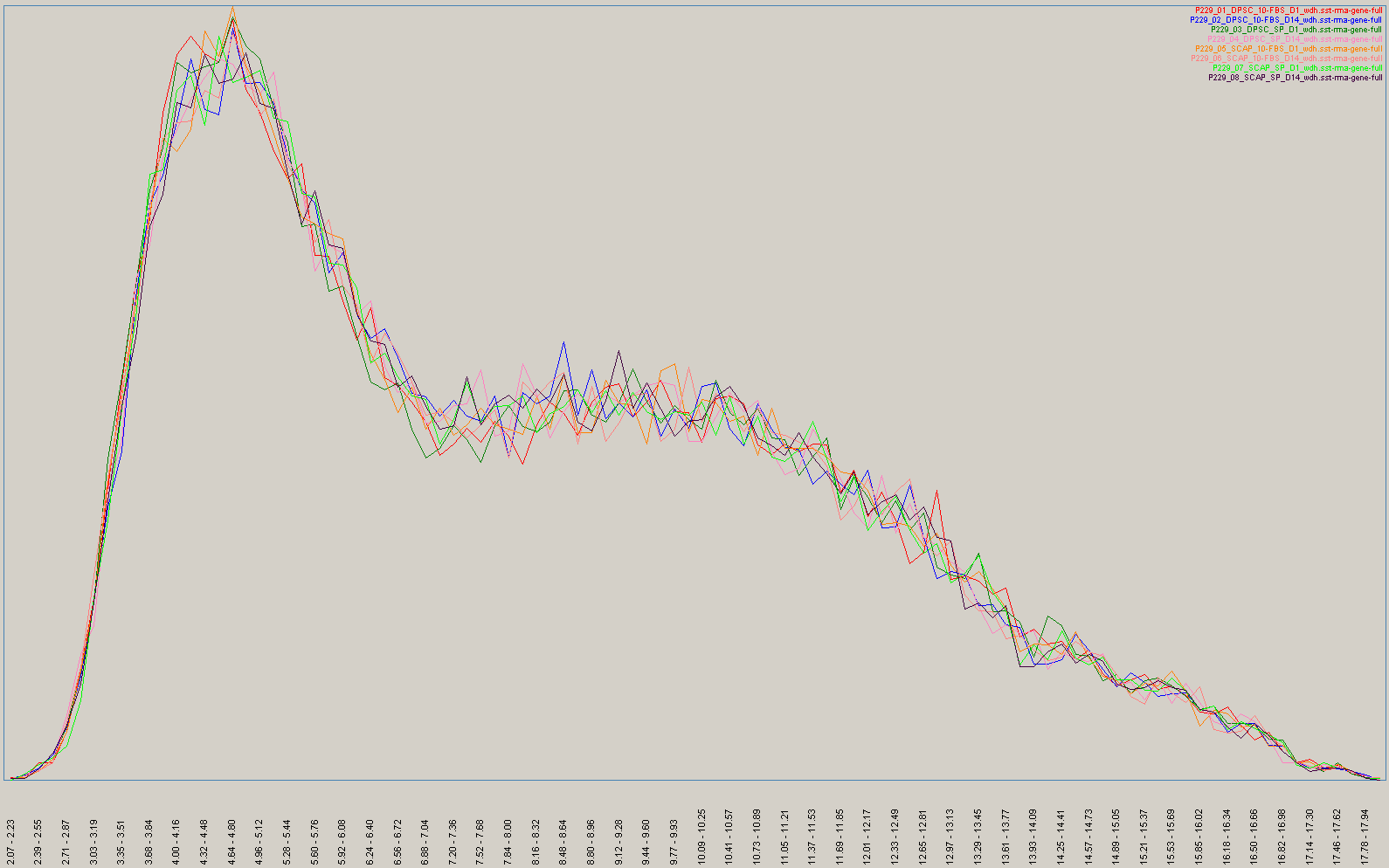

Supplement: Supplementary file 1 [file ijms-23-02615-s001.zip › Supplementary File 2/229/QC/P228_Signal Histogram.PNG]

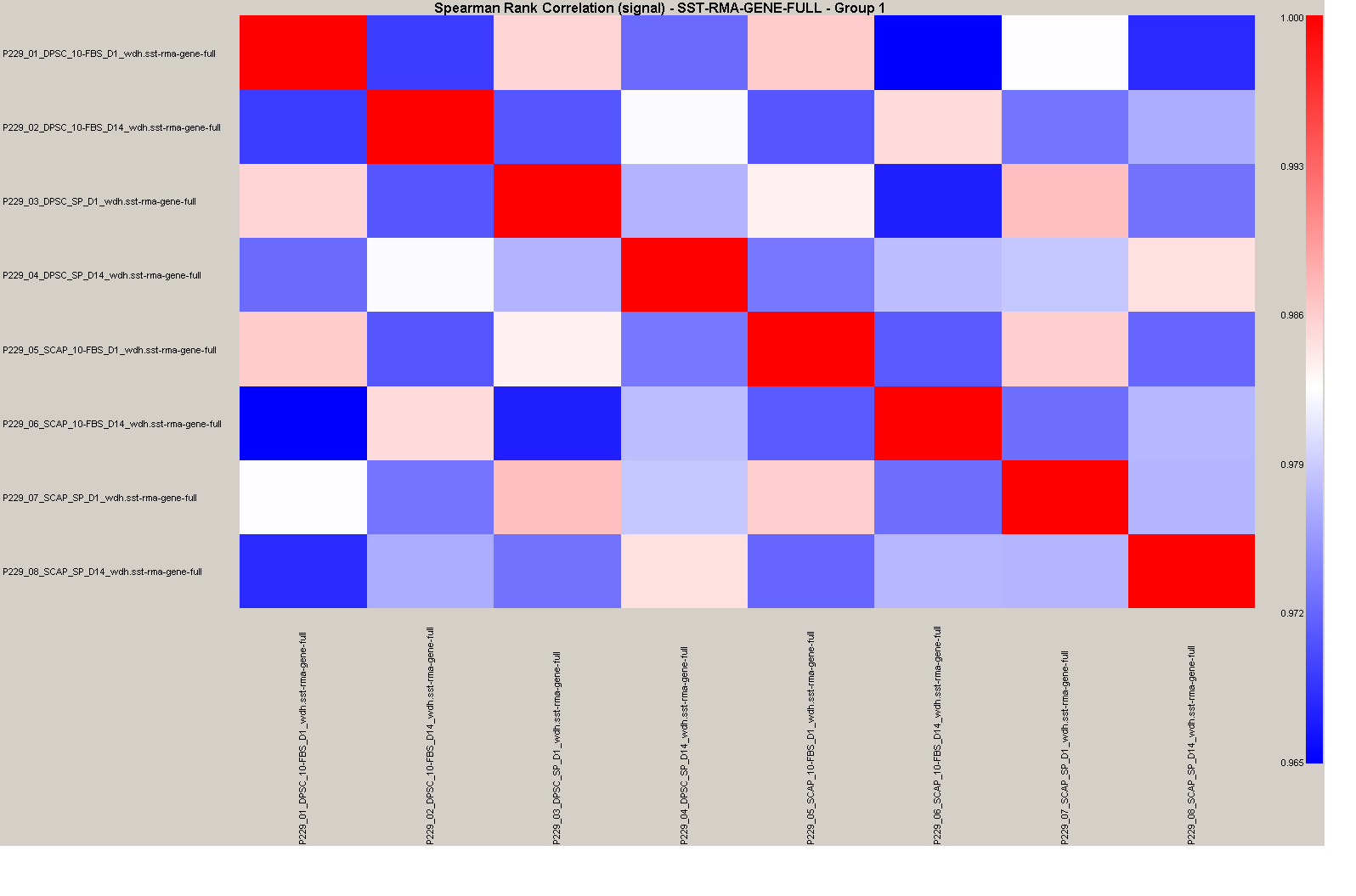

Supplement: Supplementary file 1 [file ijms-23-02615-s001.zip › Supplementary File 2/229/QC/P228_Spearman Rank Correlation.PNG]
